# Novel Decision Modeling for Manufacturing Sustainability under Single-Valued Neutrosophic Hesitant Fuzzy Rough Aggregation Information

**DOI:** 10.1155/2022/7924094

**Published:** 2022-11-28

**Authors:** Muhammad Kamran, Nadeem Salamat, Shahzaib Ashraf, Md. Ashraful Alam, Ismail Naci Cangul

**Affiliations:** ^1^Institute of Mathematics, Khwaja Fareed University of Engineering & Information Technology Rahim, Yar Khan 64200, Punjab, Pakistan; ^2^Department of Mathematics, Jahangirnagar University, Savar, Dhaka, Bangladesh; ^3^Department of Mathematics, Bursa Uludag University, Gorukle 16059, Turkey

## Abstract

We developed a multicriteria decision-making method based on the list of novel single-valued neutrosophic hesitant fuzzy rough (SV-NHFR) weighted averaging and geometric aggregation operators to address the uncertainty and achieve the sustainability of the manufacturing business. In addition, a case study on choosing the optimum elements for a sustainable manufacturing sector was carried out. The proposed decision support method is then compared to other relevant methodologies, and a validity test is performed to show the reliability and validity of the new methodology. Sustainability is one of the most important issues the world economy is facing today. Several industrial businesses have incurred large financial losses as a result of their ignorance of sustainability issues. Manufacturers in industrialized countries have done a decent job of making sure that their businesses are sustainable over the long run. Modern companies use a lot of modern technologies. These include blockchain, artificial intelligence (AI), the Internet of Things (IoT), big data analytics (BDA), and fuzzy logic (fuzziness). These modern technologies support the continuation of life, either directly or indirectly. Therefore, it is of utmost importance to concentrate on those elements that encourage the adoption of sustainability. The goal of this study is to provide a framework for using cutting-edge technology to increase the adoption of sustainability in manufacturing firms. Under the guidance of single-valued neutrosophic hesitant fuzzy rough (SV-NHFR) aggregate information, it was advised to place a strong emphasis on addressing sustainability, waste management, environmental protection, manufacturing cost savings, and chemicals and resources. The results suggest that the proposed technique can solve the inadequacy of the existing decision method by the SV-NHFR aggregation operators in terms of decision adaptability.

## 1. Introduction

Many researchers have used intuitionistic fuzzy sets (IFSs) to handle decision-making problems (DMPs) so far although the accuracy is not great enough at this point to handle the uncertainty. Smarandache [1] was the first to propose the neutrosophic set (NS), a philosophical discipline and mathematical tool for comprehending the origin, nature, and scope of neutralities. It is a spiritual practice that explores the origin, nature, and extent of neutralities, as well as their interactions with other ideational spectrums.

The NS generalizes the concepts of the classic set [2], fuzzy set, interval-valued fuzzy set, IFS, interval-valued IFS, paraconsistent set [3], dialetheist set, paradoxist set, and tautological set [4]. An NS is characterized by truth membership function *β*_*F*_(ϰ), indeterminacy membership function *α*_*F*_(ϰ) and falsity membership function *γ*_*F*_(ϰ), where *β*_*F*_(ϰ), *α*_*F*_(ϰ), and *γ*_*F*_(ϰ) are real standard or nonstandard elements from [0^−,^1]. Although an NS philosophically generalizes the notions of FS, IFS, and all the existing structures, it will be challenging to implement in real-world scientific and engineering situations.

This concept is critical in many contexts, such as information fusion, where data from several sensors is integrated. Recently, neutrosophic sets have primarily been used in engineering and other sectors to make decisions. Wang et al. [5] proposed a single-valued neutrosophic set (SV-NS), which can handle inaccurate, indeterminate, and incompatible data challenges. On the one hand, an SV-NS is an NS that allows us to convey ambiguity, imprecision, incompleteness, and inconsistency in the real world. It would be more suitable to employ uncertain information and inconsistent information matrix in decision-making [6–8]. The decision-making with the linguistic term with weakened hedge (LTWH) is very a useful tool [9]. SV-NSs, on the other hand, can be employed in scientific and technical applications since SV-NS theory is useful in modeling ambiguous, imprecise, and inconsistent data [10, 11]. The SV-NS is suitable for collecting imprecise, unclear, and inconsistent information in multicriteria decision-making analysis due to its ability to easily capture the ambiguous character of subjective judgments.

Many scholars paid close attention to SV-NS since it is a powerful universal systematic procedure. Ye [12] described the information energy and correlation of SV-NSs. The application of SV-NSs as a decision-making method was then explored by various authors [13]. The SV-NS sets are extremely useful for dealing with uncertainty challenges and improving accuracy in uncertainty challenges. The current research is inspired by this concept and concentrates on the SV-neutrosophic hesitant fuzzy rough aggregation context that is our new concept. The basic concept of SV-neutrosophic hesitant fuzzy rough sets is described in Ref. [14]. With the use of this concept, we can handle uncertainty challenges with accuracy without losing any information from the data. This contribution will be helpful for decision-makers to solve uncertainty challenges with great accuracy. There was a large gap in the literature that without defining the operators of the SV-neutrosophic hesitant fuzzy rough sets, we will not be able to solve each type of uncertainty challenges with this set as we solve a numerical example by using rough data sets and by fuzziness in it.

Our everyday lives have become increasingly concerned with environmental issues in recent years. Sustainable manufacturing is only one of several subcategories that fall under the larger umbrella of sustainable development. Throughout the manufacturing process, many environmental and social challenges also arise. Sustainable manufacturing practices may be used to overcome these obstacles in the production process. Ecologically friendly and resource-efficient manufacturing is the aim of sustainable manufacturing. Because these firms are financially sound, they are also safe for workers, communities, and consumers [15]. The three components of a sustainable manufacturing strategy are the selection of acceptable indicators for monitoring the sustainability of production, an assessment tool for identifying weak areas, and system improvements to strengthen the sustainable manufacturing process [16]. Sustainable manufacturing strategies are crucial for long-term success in the manufacturing industry for both large, small, and medium-sized firms (SMSF). Sustainability practices in manufacturing SMSF differ substantially from those in huge companies due to qualities such as customized management, a lack of finance, insufficient resources, increased flexibility, a lateral framework, a small number of customers, access to a limited market, and a lack of knowledge. Sustainable manufacturing in SMSF cannot be viewed as a scaled-down version of larger organizations based on these characteristics. The bulk of sustainable manufacturing strategies are built on indicators and assessment models that have been established and tested in large manufacturing companies [6, 17].

Indicators for technology assessment can be used in two ways: to evaluate a technology system's overall performance or to compare at least two technology systems. Rather than creating a generic collection of indicators suitable for all applications, Dewulf and Van Langenhove [18] recommended utilizing a “fit for purpose” method to apply indicators. Indicators are classified as descriptive, performance, or efficiency indicators and can be quantitative or qualitative [19]. When choosing acceptable indicators, a UN report [20] establishes a number of guidelines. In summary, they should be straightforward and instructive, and approaches should be simple and devoid of a huge number of subsets. Changes in the environment and accompanying human activities should be reflected in indicators. They should be precise, should be unambiguous, and should provide a comparison point. Environmental indicators include greenhouse gas emissions, energy consumption, resource renewability, emission toxicity, material reuse, waste material recoverability, and efficiency. Some of the recommended economic matrixes for industrial ovens are net sales, operational production costs, gross margin, and overhead costs [21].

Finally, societal factors are typically linked to toxicity and safety [22]. It is also critical to examine the indicator set using a multicriteria analysis method appropriate for that particular application in addition to establishing a relevant set of sustainability indicators. Tokos et al. [23], for example, were able to establish a framework for assessing integrated sustainability performance in processing sectors. Multicriteria analysis is a decision-making tool that gathers data on a variety of criteria, or indicators, to see how several objectives might be met most effectively. It allows for the evaluation of indicators with different units beside one another. Fuzzy set theory is a well-established topic within multicriteria analysis that offers a solution to problems that standard multicriteria analysis had previously been unable to solve. It is concerned with estimation rather than exact argumentation [24], enabling uncertainty to be logically addressed by assigning an acceptability grade to quantitative and qualitative data. Fuzzy indicator sets, which include both qualitative and quantitative data, have recently been shown to be a tool for assessing sustainability indicators [25] by allowing objective decision-making of indicators that are often subjective. In the case of qualitative indicators, uncertainty can be caused by imprecise measurements, average or outdated data, proxies and incomplete data, approximations in modeling, normalization and weighting [26], assessment and linguistic descriptors by experts, and their assigned values. When utilizing traditional multicriteria analysis to solve problems, uncertainty in the assessment of sustainable development presents complications. Fuzzy theory, on the other hand, is based on multivalued logic and deals with events that have no clear meaning, allowing fuzziness to characterize the degree to which an event occurs (and soft thresholds) [24].

The study established a mechanism for systematically assessing and analyzing social sustainability goals. The study's scientific worth is the establishment of a theoretical model for evaluating social sustainability projects, computation of the fuzzy social sustainability index, and the identification of weaker features. A typical crisp methodology was used to confirm the fuzzy technique's results. Fuzzy sets are assigned a degree of membership rather than being in or out. Fuzzy approaches are effective for appraising complex or ill-defined problems, making them ideal for sustainability indicators. The uncertainty of fuzzy indicators is attributed to generality, ambiguity, or vagueness rather than error or randomness.

### 1.1. Motivation

By acknowledging the global market, manufacturers can easily extend trade abroad and even operate their businesses in low-cost nations. Moreover, they have taken the initiative to reduce industrial emissions into the atmosphere in the current scenario of climate change and damage to the human ecological environment. Intriguingly, the government has also taken steps for the protection of the environment and laws for their implication and regulation, which force producers to design eco-friendly products. Sustainable production of goods can be defined as minimal environmental impact, social security of employment, and the welfare of the community and consumers during the whole span of the product's life.

Keeping in view the mentioned factors, manufacturing engineers should be more accountable and aware of environmental, economic, and social concerns because manufacturing emissions are a genius problem. Hence, a comprehensive assessment of the available alternatives, multicriteria decision-making (MCDM), is a strategy for tackling the real issues of the world that is the best solution. MCDM-based approaches are gaining popularity due to their wide use in various fields, including medicine, architecture, economics, and a lot more scientific and technological fields.

The MCDM technique has become complicated because of the complexity and uncertainty in data, making it difficult for decision-makers to get the best outcomes. Consequently, SV-NSs provide a better approach to handling such issues. Therefore, relying on the sensitivity of the problem, the simple techniques of SV-NSs are no more useful to get accuracy. So to seek better results, SV-neutrosophic hesitant fuzzy rough sets (SV-NHFRSs) have been discussed, and the analysis depends upon SV-NHFR weighted averaging, and weighted geometric operators.

The goal of the study and the task are mentioned in the following part.

The purpose of this research is to increase the sustainability of manufacturing work cells by using multicriteria decision-making. Two activities have been identified to help achieve this goal:

- Define and quantify matrix, determine and implement an appropriate weighting mechanism, and determine and execute a suitable ranking system as part of a decision-making approach
- Demonstrate the process by identifying and describing a representative work cell and utilizing the integrated sustainability assessment method

Some consequential endowment of the current study are as follows:

1. Firstly, we recall the concept of SV-neutrosophic hesitant fuzzy rough sets from literature
2. We proposed novel fundamental operational laws for SV-NHFRSs
3. Design a decision-making strategy that employs proposed aggregation operators to aggregate uncertain data for decision-making difficulties in the part of best option for manufacturing industry sustainability that is based on the Internet of things (IoT)

The remainder of this study is structured as follows: Section 1 presents some basic concepts of SV-FSs, HFSs, and rough set theory briefly. Basic notations and concepts are described in Section 2. A novel notion of SV-neutrosophic hesitant fuzzy sets (SV-NHFSs) are presented in Sections 3 and 4, respectively.  Section 5 presents a list of algebraic SV-hesitant fuzzy aggregation operators for combining uncertain data in decision-making. The validity and reliability tests are presented in Section 6 to ensure that the suggested approach is effective. This manuscript comes to a close with Section 7.

## 2. Preliminaries

In this constituent, we study the elementary concepts for hesitant fuzzy sets (HFS), neutrosophic sets (NS), single-valued neutrosophic sets (SV-NS), SV-neutrosophic hesitant fuzzy set (SV-NHFS), rough sets (RSs), SV-neutrosophic RS (SV-NRS), and SV-neutrosophic hesitant fuzzy RSs (SV-NHFRSs).

**Definition 1 .**

Let *ℕ* be a fixed set. The representation of HFS which is explained in Ref. [27] and is mathematically denoted as

(1) $$\begin{array}{c} \nabla =\left\{\langle \Upsilon ,\Delta_{l_{\nabla }}\left(\Upsilon \right)\rangle \left|\Upsilon \right.\in N\right\}\text{,} \end{array}$$

where Δ_*ℓ*_∇__(Υ) is a set of values in [0, 1], which indicate the grade of membership of the element Υ ∈ *ℕ* in ∇.

**Definition 2 .**

Assume *ℕ* is a set and Υ ∈ *ℕ*. A neutrosophic set [1], ϰ in *ℕ* is denoted as membership Δ_ϰ_(Υ), an indeterminacy Λ_ϰ_(Υ), and a falsity membership ∇_ϰ_(Υ) values. Δ_ϰ_(Υ), Λ_ϰ_(Υ), and ∇_ϰ_(Υ) are real standard and nonstandard subset of ]0^−,^1^+^[ and

(2) $$\begin{array}{c} \Delta_{\varkappa }\left(\Upsilon \right),\Lambda_{\varkappa }\left(\Upsilon \right),\nabla_{\varkappa }\left(\Upsilon \right):N⟶]0^{-,}1^{+}[\text{.} \end{array}$$

The representation of neutrosophic set (NS) ϰ is mathematically defined as

(3) $$\begin{array}{c} \varkappa =\left\{\langle \left.\Upsilon ,\Delta_{\varkappa }\left(\Upsilon \right),\Lambda_{\varkappa }\left(\Upsilon \right),\nabla_{\varkappa }\left(\Upsilon \right)\right)\rangle \left|\Upsilon \right.\in N\right\}\text{,} \end{array}$$

where

(4) $$\begin{array}{c} 0^{-}<\Delta_{\varkappa }\left(\Upsilon \right)+\Lambda_{\varkappa }\left(\Upsilon \right)+\nabla_{\varkappa }\left(\Upsilon \right)\leq 3^{+}\text{.} \end{array}$$

**Definition 3 .**

Let *ℕ* be a set and Υ ∈ *ℕ*. A single valued neutrosophic set (SV-NS) [5], *A* in *ℕ* is characterized by truth-membership function Δ_*A*_(Υ), an indeterminacy-membership function Λ_*A*_(Υ) and a falsity-membership function ∇_*A*_(Υ). Δ_*A*_(Υ), Λ_*A*_(Υ) and ∇_*A*_(Υ) are real standard and nonstandard subsets of [0,1] and then

(5) $$\begin{array}{c} \Delta_{A}\left(\Upsilon \right),\Lambda_{A}\left(\Upsilon \right),\nabla_{A}\left(\Upsilon \right):N⟶\left[0,1\right]\text{.} \end{array}$$

The representation of SV-NS *A* is mathematically defined as

(6) $$\begin{array}{c} A=\left\{\langle \left.\Upsilon ,\Delta_{A}\left(\Upsilon \right),\Lambda_{A}\left(\Upsilon \right),\nabla_{A}\left(\Upsilon \right)\right)\rangle \left|\Upsilon \right.\in N\right\}\text{,} \end{array}$$

where

(7) $$\begin{array}{c} 0<\Delta_{A}\left(\Upsilon \right)+\Lambda_{A}\left(\Upsilon \right)+\nabla_{A}\left(\Upsilon \right)\leq 3\text{.} \end{array}$$

**Definition 4 .**

Suppose Ω be a fixed set. The representation of SV-NHFS [28], then ∇ is mathematically defined as

(8) $$\begin{array}{c} \nabla =\left\{\langle \Upsilon ,\Delta_{l_{\nabla }}\left(\Upsilon \right),\Lambda_{l_{\nabla }}\left(\Upsilon \right),\nabla_{l_{\nabla }}\left(\Upsilon \right)\rangle \left|\Upsilon \right.\in \Omega \right\}\text{,} \end{array}$$

where Δ_*ℓ*_∇__(Υ), Λ_*ℓ*_∇__(Υ), ∇_*ℓ*_∇__(Υ)⟶[0,1], indicates the hesitant grade of membership, indeterminacy, and falsity of the element Υ ∈ Ω to the set ∇.

**Definition 5 .**

For a fixed set *ℑ*, the *SV* − *NHFS*Ω (see [29]) is represented mathematically as follows:

(9) $$\begin{array}{c} \Omega =\left\{\langle \Upsilon ,\Delta_{l_{\Omega }}\left(\Upsilon \right),\Lambda_{l_{\Omega }}\left(\Upsilon \right),\nabla_{l_{\Omega }}\left(\Upsilon \right)\rangle \left|\Upsilon \right.\in I\right\}\text{,} \end{array}$$

where Δ_*ℓ*_Ω__(Υ), Λ_*ℓ*_Ω__(Υ) and ∇_*ℓ*_Ω__(Υ) are in the range [0,1] and show the membership, indeterminacy, and nonmembership values sequentially. It has the following characteristics:

(10) $$\begin{array}{c} \forall \Upsilon \in I,\forall \mu_{\Omega }\left(\Upsilon \right)\in \Delta_{l_{\Omega }}\left(\Upsilon \right),\forall \lambda_{\Omega }\left(\Upsilon \right)\in \nabla_{l_{\Omega }}\left(\Upsilon \right), \end{array}$$

and

(11) $$\begin{array}{c} \forall \nu_{\Omega }\left(\Upsilon \right)\in \nabla_{l_{\Omega }}\left(\Upsilon \right)with\left(\max\;\left(\Delta_{l_{\Omega }}\left(\Upsilon \right)\right)\right)+\left(\min\;\left(\Lambda_{l_{\Omega }}\left(\Upsilon \right)\right)\right)+\left(\min\;\left(\nabla_{l_{\Omega }}\left(\Upsilon \right)\right)\right)\leq 3, \end{array}$$

and

(12) $$\begin{array}{c} \left(\min\;\left(\Delta_{l_{\Omega }}\left(\Upsilon \right)\right)\right)+\left(\min\;\left(\Lambda_{l_{\Omega }}\left(\Upsilon \right)\right)\right)+\left(\max\;\left(\nabla_{l_{\Omega }}\left(\Upsilon \right)\right)\right)\leq 3. \end{array}$$

For simplicity, we will use a pair Ω = (Δ_*ℓ*_Ω__, Λ_*ℓ*_Ω__, ∇_*ℓ*_Ω__) to mean −*NHFS*.

**Definition 6 .**

Assume Ω is a universal set and ∅ is mapping on Ω. A set valued relation (see the relation in [30]) is defined as

(13) $$\begin{array}{c} \emptyset^{*}:\Omega ⟶M\left(\Omega \right)by\;\emptyset^{*}\left(\chi \right)=\left\{a\in \Omega \left|\chi ,a\right.\in \emptyset \right\}\text{,} \end{array}$$

for Ξ ∈ Ω, where ∅^*∗*^(Ξ) is called a beneficiary neighborhood of Ξ with relation ∅. A pair (Ω, ∅) is called (crisp) surmise space. For any set, *𝕜*⊆Ω, the lower and upper (*L* ∼ *H*) surmise of *𝕜* to surmise space (Ω, ∅) is defined as

(14) $$\begin{array}{c} \underline{\emptyset }\left(k\right)=\left\{\Xi \in \Omega |\emptyset^{*}\left(\Xi \right)\subseteq k\right\}\text{,}\\ \bar{\emptyset }\left(k\right)=\left\{\Xi \in \Omega |\emptyset^{*}\left(\Xi \right)\cap k\neq \phi \right\}\text{.} \end{array}$$

The pair $\left(\underline{\emptyset }\left(𝕜\right),\bar{\emptyset }\left(𝕜\right)\right)$ is called the fuzzy rough set and both $\underline{\emptyset }\left(𝕜\right),\bar{\emptyset }\left(𝕜\right):M\left(\Omega \right)⟶M\left(\Omega \right)$ are *L* ∼ *H* surmise operators.

**Definition 7 .**

Let universal set Ω and let í∈ SV-NHFRS (Ω × Ω) be SV − NF relation [31], then

(i) is reflexive if
  (15) $$\begin{array}{c} \Delta \left(\Upsilon ,\Upsilon \right)=1,\Delta \left(\Upsilon ,\Upsilon \right)=1\;an\;d\nabla \left(\Upsilon ,\Upsilon \right)=1,\forall \Upsilon \in \Omega \text{.} \end{array}$$
(ii) is symmetric if
  (16) $$\begin{array}{c} \forall \left(\Upsilon ,r\right)\in \left(\Omega \times \Omega \right),\Delta_{\text{í}}\left(\Upsilon ,r\right)=\Delta_{\text{í}}\left(r,\Upsilon \right),\Lambda_{\text{í}}\left(\Upsilon ,r\right)=\Lambda_{\text{í}}\left(r,\Upsilon \right)an\;d\;\nabla_{\text{í}}\left(\Upsilon ,r\right)=\nabla_{\text{í}}\left(r,\Upsilon \right)\text{.} \end{array}$$
(iii) is transitive if ∀(Υ, *δ*) ∈ (Ω × Ω),
  (17) $$\begin{array}{c} \Delta \left(r,\delta \right)\geq \vee_{\Upsilon \in \Omega }\left[\Delta \left(r,\Upsilon \right)\wedge \Delta_{\text{í}}\left(\Upsilon ,\delta \right)\right]\text{,}\\ \Delta \left(r,\delta \right)=\wedge_{\Upsilon \in \Omega }\left[\Delta \left(r,\Upsilon \right)\vee \Lambda_{\text{í}}\left(\Upsilon ,\delta \right)\right]\text{,} \end{array}$$
and
  (18) $$\begin{array}{c} \nabla \left(r,\delta \right)=\wedge_{\Upsilon \in \Omega }\left[\nabla \left(r,\Upsilon \right)\vee \nabla \left(\Upsilon ,\delta \right)\right]\text{.} \end{array}$$

**Definition 8 .**

Let universal set Ω and let í∈ SV-NHFRS (Ω × Ω) be SV-NF relation [32]. The pair (Ω) represents a V-NF approximation space. Let *ℓ* be any subset of SV-NS (Ω), i.e., *ℓ*⊆ SV-NS (Ω). Then on the basis of SV-NF approximation space (Ω), then the *L* ∼ *H* surmises of *ℓ* are represented as (*ℓ*) and (*ℓ*) that is given as follows:

(19) $$\begin{array}{c} \left(l\right)=\left\{\langle \Upsilon ,\Delta_{\left(l\right)}\left(\Upsilon \right),\Lambda_{\left(l\right)}\left(\Upsilon \right),\nabla_{\left(l\right)}\left(\Upsilon \right)\rangle \left|\Upsilon \right.\in \Omega \right\}\text{,}\\ \left(l\right)=\left\{\langle \Upsilon ,\Delta_{\underline{\text{í}}\left(l\right)}\left(\Upsilon \right),\Lambda_{\left(l\right)}\left(\Upsilon \right),\nabla_{\left(l\right)}\left(\Upsilon \right)\rangle \left|\Upsilon \right.\in \Omega \right\}\text{,} \end{array}$$

where

(20) $$\begin{array}{c} \Delta_{\bar{\text{í}}\left(l\right)}\left(\Upsilon \right)=\vee_{\delta \in \Omega }\left[\Delta_{ℶ}\left(\Upsilon ,\delta \right)\vee \Delta_{ℶ}\left(\delta \right)\right]\text{,}\\ \Lambda_{\bar{\text{í}}\left(l\right)}\left(\Upsilon \right)=\wedge_{\delta \in \Omega }\left[\Lambda_{ℶ}\left(\Upsilon ,\delta \right)\wedge \Lambda_{ℶ}\left(\delta \right)\right]\text{,}\\ \nabla_{\bar{\text{í}}\left(l\right)}\left(\Upsilon \right)=\wedge_{\delta \in \Omega }\left[\nabla_{ℶ}\left(\Upsilon ,\delta \right)\wedge \nabla_{ℶ}\left(\delta \right)\right]\text{,}\\ \Lambda_{\bar{\text{í}}\left(l\right)}\left(\Upsilon \right)=\wedge_{\delta \in \Omega }\left[\Lambda_{ℶ}\left(\Upsilon ,\delta \right)\wedge \Lambda_{ℶ}\left(\delta \right)\right]\text{,}\\ \nabla_{\bar{\text{í}}\left(l\right)}\left(\Upsilon \right)=\vee_{\delta \in \Omega }\left[\nabla_{ℶ}\left(\Upsilon ,\delta \right)\vee \nabla_{ℶ}\left(\delta \right)\right]\text{,} \end{array}$$

such that

(21) $$\begin{array}{c} 0<\Delta_{\left(l\right)}\left(\Upsilon \right)+\Lambda_{\left(l\right)}\left(\Upsilon \right)+\nabla_{\left(l\right)}\left(\Upsilon \right)\leq 3\text{,} \end{array}$$

and

(22) $$\begin{array}{c} 0<\Delta_{\left(l\right)}\left(\Upsilon \right)+\Lambda_{\left(l\right)}\left(\Upsilon \right)+\nabla_{\left(l\right)}\left(\Upsilon \right)\leq 3\text{.} \end{array}$$

As (*ℓ*) and (*ℓ*) are SV − NFSs, so (*ℓ*), (*ℓ*):SV-NFS (Ω)⟶ SV-NFS (Ω) are *L* ∼ *H* surmise operators. So the pair

(23) $$\begin{array}{c} \left(l\right)=\left(\left(l\right),\left(l\right)\right)=\left\{\Upsilon ,\langle \left(\Delta_{\left(l\right)}\left(\Upsilon \right),\Lambda_{\left(l\right)}\left(\Upsilon \right),\nabla_{\left(l\right)}\left(\Upsilon \right)\right)),\left(\Delta_{\left(l\right)}\left(\Upsilon \right),\Lambda_{\left(l\right)}\left(\Upsilon \right),\nabla_{\left(l\right)}\left(\Upsilon \right)\right)\left|\Upsilon \right.\in \Omega \right\}, \end{array}$$

is called the SV-NF rough set. For simplicity, it can be denoted as $\left(\ell \right)=\left(\left(\ell \right),\left(\ell \right)\right)=\left(\left(\underline{\Delta },\underline{\Lambda },\underline{\nabla }\right),\left(\bar{\Delta },\bar{\Lambda },\bar{\nabla }\right)\right)$ and are known as the SV-NF rough number (SV-NFRN).

**Example 1 .**

Suppose £ = {Ξ_1_, Ξ_2_, Ξ_3_, Ξ_4_} is an arbitrary set and (£, ∅) is the SV − NHF approximation space with ∅∈SV − NHFRS(£×£) which is the SV − NHFR mapping, as given in Table 1. Now a decision professional presents the optimum normal decision object *𝕜* which is a *SV* − *NHFS*.

and

(24) $$\begin{array}{c} k=\left\{\begin{array}{c} \langle \Xi_{1},\left\{0.2,0.1,0.4\right\},\left\{0.5,0.2,0.7\right\},\left\{0.3,0.4,0.9\right\}\rangle \text{,}\\ \langle \Xi_{2},\left\{0.1,0.3,0.3\right\},\left\{0.1,0.5,0.8\right\},\left\{0.3,0.2,0.5\right\}\rangle \text{,}\\ \langle \Xi_{3},\left\{0.5,0.4,0.8\right\},\left\{0.1,0.2,0.7\right\},\left\{0.2,0.4,0.5\right\}\rangle \text{,}\\ \langle \Xi_{4},\left\{0.6,0.8,0.9\right\},\left\{0.2,0.6,0.7\right\},\left\{0.3,0.5,0.3\right\}\rangle \end{array}\right\}\text{.} \end{array}$$

Then, it follows that

(25) $$\begin{array}{c} \Delta_{\left(l\right)}\left(\Xi_{1}\right)=\vee_{\delta \in \Omega }\left[\Delta \left(\Xi ,\delta \right)\vee \Delta \left(\delta \right)\right]=\left\{\begin{array}{c} \left\{0.2\vee 0.2,0.5\vee 0.1,0.4\vee 0.4\right\}\vee \\ \left\{0\vee 0.1,0.1\vee 0.3,0.5\vee 0.3\right\}\vee \\ \left\{0.1\vee 0.5,0.6\vee 0.4,0.7\vee 0.8\right\}\vee \\ \left\{0.3\vee 0.6,0.4\vee 0.8,0.5\vee 0.9\right\} \end{array}\right\}\\ =\left\{\begin{array}{c} \left\{0.2,0.5,0.4\right\}\vee \left\{0.1,0.3,0.5\right\}\vee \\ \left\{0.5,0.6,0.8\right\}\vee \left\{0.6,0.8,0.9\right\} \end{array}\right\}\\ =\left\{0.6,0.8,0.9\right\}. \end{array}$$

In a similar way, we obtain the other values:

(26) $$\begin{array}{c} \Delta_{\left(l\right)}\left(\Xi_{2}\right)=\left\{0.6,0.8,0.9\right\}\text{,}\\ \Delta_{\left(l\right)}\left(\Xi_{3}\right)=\left\{0.7,0.8,0.9\right\}\text{,}\\ \Delta_{\left(l\right)}\left(\Xi_{4}\right)=\left\{0.6,0.8,0.9\right\}\text{.} \end{array}$$

Similarly,

(27) $$\begin{array}{c} \Lambda_{\left(l\right)}\left(\Upsilon \right)=\wedge_{\delta \in \Omega }\left[\Lambda \left(\Upsilon ,\delta \right)\wedge \Lambda \left(\delta \right)\right]=\left\{\begin{array}{c} \left\{0.7\wedge 0.5,0.6\wedge 0.2,0.7\wedge 0.7\right\}\wedge \\ \left\{0.8\wedge 0.1,0.9\wedge 0.5,0.4\wedge 0.8\right\}\wedge \\ \left\{0.7\wedge 0.1,0.3\wedge 0.2,0\wedge 0.7\right\}\wedge \\ \left\{0.8\wedge 0.2,0\wedge 0.6,0\wedge 0.7\right\} \end{array}\right\}=\left\{\begin{array}{c} \left\{0.5,0.2,0.7\right\}\wedge \left\{0.1,0.5,0.4\right\}\wedge \\ \left\{0.1,0.2,0\right\}\wedge \left\{0.2,0,0\right\} \end{array}\right\}=\left\{0.1,0,0\right\}\text{.} \end{array}$$

In a similar way, we obtain the other values:

(28) $$\begin{array}{c} \Lambda_{\left(l\right)}\left(\Xi_{2}\right)=\left\{0.2,0,0\right\}\text{,}\\ \Lambda_{\left(l\right)}\left(\Xi_{3}\right)=\left\{0.1,0.2,0\right\}\text{,}\\ \Lambda_{\left(l\right)}\left(\Xi_{4}\right)=\left\{0.1,0.2,0\right\}\text{.} \end{array}$$

Similarly,

(29) $$\begin{array}{c} \nabla_{\bar{\text{í}}\left(l\right)}\left(\Upsilon \right)=\wedge_{\delta \in \Omega }\left[\nabla_{\text{í}}\left(\Upsilon ,\delta \right)\wedge \nabla_{\text{í}}\left(\delta \right)\right]\text{,}\\ =\left\{\begin{array}{c} \left\{0.7\wedge 0.3,0.3\wedge 0.4,0.1\wedge 0.9\right\}\wedge \\ \left\{0.3\wedge 0.3,0.2\wedge 0.2,0.9\wedge 0.5\right\}\wedge \\ \left\{0.5\wedge 0.2,0.8\wedge 0.4,0.2\wedge 0.5\right\}\wedge \\ \left\{0.4\wedge 0.3,0.7\wedge 0.5,0.3\wedge 0.3\right\} \end{array}\right\}\text{,}\\ =\left\{\begin{array}{c} \left\{0.3,0.3,0.1\right\}\wedge \left\{0.3,0.2,0.5\right\}\wedge \\ \left\{0.2,0.4,0.2\right\}\wedge \left\{0.3,0.5,0.3\right\} \end{array}\right\}\text{,}\\ =\left\{0.2,0.2,0.1\right\}\text{.} \end{array}$$

In a similar way, we obtain the other values:

(30) $$\begin{array}{c} \nabla_{\bar{\text{í}}\left(l\right)}\left(\Xi_{2}\right)=\left\{0.2,0.1,0\right\}\text{,}\\ \nabla_{\bar{\text{í}}\left(l\right)}\left(\Xi_{3}\right)=\left\{0.2,0.2,0.1\right\}\text{,}\\ \nabla_{\bar{\text{í}}\left(l\right)}\left(\Xi_{4}\right)=\left\{0.2,0.2,0\right\}\text{.} \end{array}$$

For lower approximation,

(31) $$\begin{array}{c} \Delta_{\underline{\text{í}}\left(l\right)}\left(\Xi_{1}\right)=\wedge_{\delta \in \Omega }\left[\Delta_{\text{í}}\left(\Upsilon ,\delta \right)\wedge \Delta_{\text{í}}\left(\delta \right)\right]\text{,}\\ =\left\{\begin{array}{c} \left\{0.2\wedge 0.2,0.5\wedge 0.1,0.4\wedge 0.4\right\}\wedge \\ \left\{0\wedge 0.1,0.1\wedge 0.3,0.5\wedge 0.3\right\}\wedge \\ \left\{0.1\wedge 0.5,0.6\wedge 0.4,0.7\wedge 0.8\right\}\wedge \\ \left\{0.3\wedge 0.6,0.4\wedge 0.8,0.5\wedge 0.9\right\} \end{array}\right\}\text{,}\\ =\left\{\begin{array}{c} \left\{0.2,0.1,0.4\right\}\wedge \left\{0,0.1,0.3\right\}\wedge \\ \left\{0.1,0.4,0.7\right\}\wedge \left\{0.3,0.4,0.5\right\} \end{array}\right\}\text{,}\\ =\left\{0,0.1,0.3\right\}\text{.} \end{array}$$

In a similar way, we obtain the other values:

(32) $$\begin{array}{c} \Delta_{\underline{\text{í}}\left(l\right)}\left(\Xi_{2}\right)=\left\{0.1,0.1,0\right\}\text{,}\\ \Delta_{\underline{\text{í}}\left(l\right)}\left(\Xi_{3}\right)=\left\{0.1,0.1,0\right\}\text{,}\\ \Delta_{\underline{\text{í}}\left(l\right)}\left(\Xi_{4}\right)=\left\{0.1,0.1,0\right\}\text{.}\\ \Lambda_{\underline{\text{í}}\left(l\right.}\left(\Upsilon \right)=\wedge_{\delta \in \Omega }\left[\Lambda_{\text{í}}\left(\Upsilon ,\delta \right)\wedge \Lambda_{\text{í}}\left(\delta \right)\right]\text{,}\\ =\left\{\begin{array}{c} \left\{0.7\wedge 0.5,0.6\wedge 0.2,0.7\wedge 0.7\right\}\wedge \\ \left\{0.8\wedge 0.1,0.9\wedge 0.5,0.4\wedge 0.8\right\}\wedge \\ \left\{0.7\wedge 0.1,0.3\wedge 0.2,0\wedge 0.7\right\}\wedge \\ \left\{0.8\wedge 0.2,0\wedge 0.6,0\wedge 0.7\right\} \end{array}\right\}\text{,}\\ =\left\{\begin{array}{c} \left\{0.5,0.2,0.7\right\}\wedge \left\{0.1,0.5,0.4\right\}\wedge \\ \left\{0.1,0.2,0\right\}\wedge \left\{0.2,0,0\right\} \end{array}\right\}\text{,}\\ =\left\{0.1,0,0\right\}\text{.} \end{array}$$

In a similar way, we obtain the other values:

(33) $$\begin{array}{c} \begin{array}{c} \Lambda_{\underline{\text{í}}\left(l\right.}\left(\Xi_{2}\right)=\left\{0.2,0,0\right\}\text{,}\\ \Lambda_{\underline{\text{í}}\left(l\right.}\left(\Xi_{3}\right)=\left\{0.1,0.2,0\right\}\text{,}\\ \Lambda_{\underline{\text{í}}\left(l\right.}\left(\Xi_{4}\right)=\left\{0.1,0.2,0\right\}\text{.}\\ \nabla_{\underline{\text{í}}\left(l\right)}\left(\Xi_{1}\right)=\vee_{\delta \in \Omega }\left[\nabla_{\text{í}}\left(\Xi ,\delta \right)\vee \nabla_{\text{í}}\left(\delta \right)\right]\text{,}\\ =\left\{\begin{array}{c} \left\{0.2\vee 0.2,0.5\vee 0.1,0.4\vee 0.4\right\}\vee \\ \left\{0\vee 0.1,0.1\vee 0.3,0.5\vee 0.3\right\}\vee \\ \left\{0.1\vee 0.5,0.6\vee 0.4,0.7\vee 0.8\right\}\vee \\ \left\{0.3\vee 0.6,0.4\vee 0.8,0.5\vee 0.9\right\} \end{array}\right\}\text{,}\\ =\left\{\begin{array}{c} \left\{0.2,0.5,0.4\right\}\vee \left\{0.1,0.3,0.5\right\}\vee \\ \left\{0.5,0.6,0.8\right\}\vee \left\{0.6,0.8,0.9\right\} \end{array}\right\}\text{,}\\ =\left\{0.6,0.8,0.9\right\}\text{.} \end{array}\text{.} \end{array}$$

In a similar way, we obtain the other values:

(34) $$\begin{array}{c} \nabla_{\underline{\text{í}}\left(l\right)}\left(\Xi_{2}\right)=\left\{0.6,0.8,0.9\right\}\text{,}\\ \nabla_{\underline{\text{í}}\left(l\right)}\left(\Xi_{3}\right)=\left\{0.7,0.8,0.9\right\}\text{,}\\ \nabla_{\underline{\text{í}}\left(l\right)}\left(\Xi_{4}\right)=\left\{0.6,0.8,0.9\right\}\text{.} \end{array}$$

Thus, *L* ∼ *H* SV-NHFR sets are as follows:

(35) $$\begin{array}{c} \bar{\text{í}}\left(l\right)=\left\{\begin{array}{c} \langle \Xi_{1},\left(0.6,0.8,0.9\right),\left(0.1,0,0\right),\left(0.2,0.2,0.1\right)\rangle \text{,}\\ \langle \Xi_{2},\left(0.6,0.8,0.9\right),\left(0.2,0,0\right),\left(0.2,0.1,0\right)\rangle \text{,}\\ \langle \Xi_{3},\left(0.7,0.8,0.9\right),\left(0.1,0.2,0\right),\left(0.2,0.2,0.1\right)\rangle \text{,}\\ \langle \Xi_{4},\left(0.6,0.8,0.9\right)\left(0.1,0.2,0\right),\left(0.6,0.8,0.9\right)\rangle \text{.} \end{array}\right\} \end{array}$$

(36) $$\begin{array}{c} \underline{\text{í}}\left(l\right)=\left\{\begin{array}{c} \langle \Xi_{1},\left(0,0.1,0.3\right),\left(0.1,0,0\right),\left(0.6,0.8,0.9\right)\rangle \text{,}\\ \langle \Xi_{2},\left(0.1,0.1,0\right),\left(0.2,0,0\right),\left(0.6,0.8,0.9\right)\rangle \text{,}\\ \langle \Xi_{3},\left(0.1,0.1,0\right),\left(0.1,0.2,0\right),\left(0.7,0.8,0.9\right)\rangle \text{,}\\ \langle \Xi_{4},\left(0.1,0.1,0\right),\left(0.1,0.2,0\right),\left(0.6,0.8,0.9\right)\rangle \text{,} \end{array}\right\} \end{array}$$

where

(37) $$\begin{array}{c} \text{í}\left(l\right)=\left(\bar{\text{í}}\left(l\right),\underline{\text{í}}\left(l\right)\right)\text{,}\\ =\left\{\begin{array}{c} \left\{\langle \Xi_{1}),\left(0.6,0.8,0.9\right),\left(0.1,0,0\right),\left(0.2,0.2,0.1\right)\text{,}\right.\\ \left(0,0.1,0.3\right),\left(0.1,0,0\right),\left(0.6,0.8,0.9\right)\left((\rangle \right\}\text{,}\\ \left\{\langle \Xi_{2}),\left(0.6,0.8,0.9\right),\left(0.2,0,0\right),\left(0.2,0.1,0\right)\text{,}\right.\\ \left(0.1,0.1,0\right),\left(0.2,0,0\right),\left(0.6,0.8,0.9\right)\left((\rangle \right\}\text{,}\\ \left\{\langle \Xi_{3}),\left(0.7,0.8,0.9\right),\left(0.1,0.2,0\right),\left(0.2,0.2,0.1\right)\text{,}\right.\\ \left(0.1,0.1,0\right),\left(0.1,0.2,0\right),\left(0.7,0.8,0.9\right)\left((\rangle \right\}\text{,}\\ \left\{\langle \Xi_{4}),\left(0.6,0.8,0.9\right)\left(0.1,0.2,0\right),\left(0.6,0.8,0.9\right)\text{,}\right.\\ \left(\left(0.1,0.1,0\right),\left(0.1,0.2,0\right),\left(0.6,0.8,0.9\right)(\rangle \right\} \end{array}\right\}\text{.} \end{array}$$

**Definition 9 (see [33]).**

Let *ℝ*_1_ = (Δ_*ℓ*_*ℝ*_1___, Λ_*ℓ*_*ℝ*_1___, ∇_*ℓ*_*ℝ*_1___) and *ℝ*_2_ = (Δ_*ℓ*_*ℝ*_2___, Λ_*ℓ*_*ℝ*_2___, ∇_*ℓ*_*ℝ*_2___) be two SV-NHFNs.

The following are the basic appropriate measures actions:

(38) $$\begin{array}{c} R_{1}\cup R_{2}=\left\{\underset{\underset{\mu_{2}\in \Delta_{l_{R_{2}}}}{\mu_{1}\in \Delta_{l_{R_{1}}}}}{\cup }\max\;\left(\mu_{1},\mu_{2}\right),\underset{\underset{\nu_{2}\in \Lambda_{l_{R_{2}}}}{\nu_{1}\in \Lambda_{l_{R_{1}}}}}{\cup }\min\;\left(\nu_{1},\nu_{2}\right),\underset{\underset{\lambda_{2}\in \nabla_{l_{R_{2}}}}{\lambda_{1}\in \nabla_{l_{R_{1}}}}}{\cup }\min\;\left(\lambda_{1},\lambda_{2}\right)\right\}\text{,}\\ R_{1}\cap R_{2}=\left\{\underset{\underset{\mu_{2}\in \Delta_{l_{R_{2}}}}{\mu_{1}\in \Delta_{l_{R_{1}}}}}{\cup }\min\;\left(\mu_{1},\mu_{2}\right),\underset{\underset{\nu_{2}\in \Lambda_{l_{R_{2}}}}{\nu_{1}\in \Lambda_{l_{R_{1}}}}}{\cup }\max\;\left(\nu_{1},\nu_{2}\right),\underset{\underset{\lambda_{2}\in \nabla_{l_{R_{2}}}}{\lambda_{1}\in \nabla_{l_{R_{1}}}}}{\cup }\max\;\left(\lambda_{1},\lambda_{2}\right)\right\}\text{,}\\ R_{1}^{c}=\left\{\nabla_{l_{R_{1}}},\Lambda_{l_{R_{1}}},\Delta_{l_{R_{1}}}\right\}\text{.} \end{array}$$

**Definition 10 (see [30]).**

Assume a universal set Ω with ∅⊆Ω × Ω be a (crisp) mapping. Then

1. ∅ is reflexive if (Ξ, Ξ) ∈ ∅, for every Ξ ∈ Ω
2. ∅ is symmetric if ∀Ξ, *a* ∈ Ω, (Ξ, *a*) ∈ ∅, then (*a*, Ξ) ∈ ∅
3. ∅ is transitive if ∀Ξ, *a*, *b* ∈ Ω, (Ξ, *a*) ∈ Ω and (*a*, *b*) ∈ ∅⟶(Ξ, *b*) ∈ ∅

## 3. Construction of Single-Valued Neutrosophic Hesitant Fuzzy Rough Sets

In this dissertation, we introduce the concept of SV-NF rough set (SV-NHFRS), which is a hybrid rough set structure.

We also introduce the SV-NHFRS's scoring and accuracy features, as well as its basic operational regulations.

**Definition 11 .**

Assume universal set Ω and let í ∈ SV − NHFRS(Ω × Ω) be SV − NHF relation. The pair (Ω, í) represents a SV − NHF approximation space. Let *ℓ* be any subset of SV − NHS(Ω), i.e., *ℓ*⊆SV − NHS(Ω). Then on the basis of SV − NHF approximation space (Ω, í), the *L* ∼ *H* approximations of *ℓ* are represented as $\bar{\text{í}}\left(\ell \right)$ and $\underline{\text{í}}\left(\ell \right)$ and given as follows:

(39) $$\begin{array}{c} \bar{\text{í}}\left(l\right)=\left\{\langle \Upsilon ,\Delta_{\bar{\text{í}}\left(l\right)}\left(\Upsilon \right),\Lambda_{\bar{\text{í}}\left(l\right)}\left(\Upsilon \right),\nabla_{\bar{\text{í}}\left(l\right)}\left(\Upsilon \right)\rangle \left|\Upsilon \right.\in \Omega \right\}\text{,}\\ \underline{\text{í}}\left(l\right)=\left\{\langle \Upsilon ,\Delta_{\underline{\text{í}}\left(l\right)}\left(\Upsilon \right),\Lambda_{\underline{\text{í}}\left(l\right)}\left(\Upsilon \right),\nabla_{\underline{\text{í}}\left(l\right)}\left(\Upsilon \right)\rangle \left|\Upsilon \right.\in \Omega \right\}\text{,} \end{array}$$

where

(40) $$\begin{array}{c} \Delta_{\bar{\text{í}}\left(l\right)}\left(\Upsilon \right)=\vee_{\delta \in \Omega }\left[\Delta_{\text{í}}\left(\Upsilon ,\delta \right)\vee \Delta_{\text{í}}\left(\delta \right)\right]\text{,}\\ \Lambda_{\bar{\text{í}}\left(l\right)}\left(\Upsilon \right)=\wedge_{\delta \in \Omega }\left[\Lambda_{\text{í}}\left(\Upsilon ,\delta \right)\wedge \Lambda_{\text{í}}\left(\delta \right)\right]\text{,}\\ \nabla_{\bar{\text{í}}\left(l\right)}\left(\Upsilon \right)=\wedge_{\delta \in \Omega }\left[\nabla_{\text{í}}\left(\Upsilon ,\delta \right)\wedge \nabla_{\text{í}}\left(\delta \right)\right]\text{,}\\ \Delta_{\underline{\text{í}}\left(l\right)}\left(\Upsilon \right)=\wedge_{\delta \in \Omega }\left[\Delta_{\text{í}}\left(\Upsilon ,\delta \right)\wedge \Delta_{\text{í}}\left(\delta \right)\right]\text{,}\\ \Lambda_{\underline{\text{í}}\left(l\right)}\left(\Upsilon \right)=\wedge_{\delta \in \Omega }\left[\Lambda_{\text{í}}\left(\Upsilon ,\delta \right)\wedge \Lambda_{\text{í}}\left(\delta \right)\right]\text{,}\\ \nabla_{\underline{\text{í}}\left(l\right)}\left(\Upsilon \right)=\vee_{\delta \in \Omega }\left[\nabla_{\text{í}}\left(\Upsilon ,\delta \right)\vee \nabla_{\text{í}}\left(\delta \right)\right]\text{,} \end{array}$$

such that

(41) $$\begin{array}{c} 0<\Delta_{\bar{\text{í}}\left(l\right)}\left(\Upsilon \right)+\Lambda_{\bar{\text{í}}\left(l\right)}\left(\Upsilon \right)+\nabla_{\bar{\text{í}}\left(l\right)}\left(\Upsilon \right)\leq 3\text{,} \end{array}$$

and

(42) $$\begin{array}{c} 0<\Delta_{\underline{\text{í}}\left(l\right)}\left(\Upsilon \right)+\Lambda_{\underline{\text{í}}\left(l\right)}\left(\Upsilon \right)+\nabla_{\underline{\text{í}}\left(l\right)}\left(\Upsilon \right)\leq 3\text{.} \end{array}$$

As $\underline{\text{í}}\left(\ell \right)$ and $\bar{\text{í}}\left(\ell \right)$ are SV − NFSs, so $\bar{\text{í}}\left(\ell \right),\underline{\text{í}}\left(\ell \right):SV-NFS\left(\Omega \right)⟶SV-NFS\left(\Omega \right)$ are *L* ∼ *H* approximation operators. So the pair

(43) $$\begin{array}{c} \text{í}\left(l\right)=\left(\underline{\text{í}}\left(l\right),\bar{\text{í}}\left(l\right)\right)\text{,}\\ =\left\{\Upsilon ,\langle \left(\Delta_{\underline{\text{í}}\left(l\right)}\left(\Upsilon \right),\Lambda_{\underline{\text{í}}\left(l\right)}\left(\Upsilon \right),\nabla_{\underline{\text{í}}\left(l\right)}\left(\Upsilon \right)\right)),\left(\Delta_{\bar{\text{í}}\left(l\right)}\left(\Upsilon \right),\Lambda_{\bar{\text{í}}\left(l\right)}\left(\Upsilon \right),\nabla_{\bar{\text{í}}\left(l\right)}\left(\Upsilon \right)\right)\left|\Upsilon \right.\in \Omega \right\}, \end{array}$$

is called the SV − NHF rough set. For simplicity, it can be denoted as

(44) $$\begin{array}{c} \text{í}\left(l\right)=\left(\left(l\right),\left(l\right)\right)\text{,}\\ =\left\{\left({\underline{\Delta }}_{l\left(\Upsilon \right)},{\underline{\Lambda }}_{l\left(\Upsilon \right)},{\underline{\nabla }}_{l\left(\Upsilon \right)}\right),\left({\bar{\Delta }}_{l\left(\Upsilon \right)},{\bar{\Lambda }}_{l\left(\Upsilon \right)},{\bar{\nabla }}_{l\left(\Upsilon \right)}\right)\right\}, \end{array}$$

is known as the SV − NHF rough number (SV − NHFRN).

**Definition 12 .**

Let Ω be the universal set, then any subset ∅∈SV − NHFRS(Ω × Ω) is known an SV-neutrosophic hesitant fuzzy mapping. The pair (Ω, ∅) is called SV-NHFRS approximation space. If for any *𝕜*⊆SV − NHFRS(Ω), then the *L* ∼ *H* operators of *𝕜* to SV-NHFRS approximation space (Ω, ∅) are two SV-NHFRSs, which are given by $\bar{\emptyset }\left(𝕜\right)$ and $\underline{\emptyset }\left(𝕜\right)$ and are defined as

(45) $$\begin{array}{c} \bar{\emptyset }\left(k\right)=\left\{\langle \Xi ,\Delta_{l_{\bar{\emptyset }\left(k\right)}}\left(\Xi \right),\Lambda_{l_{\bar{\emptyset }\left(k\right)}}\left(\Xi \right),\nabla_{l_{\bar{\emptyset }\left(k\right)}}\left(\Xi \right)\rangle \left|\Xi \right.\in \Omega \right\}\text{,}\\ \underline{\emptyset }\left(k\right)=\left\{\langle \Xi ,\Delta_{l_{\underline{\emptyset }\left(k\right)}}\left(\Xi \right),\Lambda_{l_{\underline{\emptyset }\left(k\right)}}\left(\Xi \right),\nabla_{l_{\underline{\emptyset }\left(k\right)}}\left(\Xi \right)\rangle \left|\Xi \right.\in \Omega \right\}\text{,} \end{array}$$

where

(46) $$\begin{array}{c} \Delta_{l_{\bar{\emptyset }\left(k\right)}}\left(\Xi \right)=\underset{k\in \Omega }{\vee }\left[\Delta_{l_{\emptyset }}\left(\Xi ,k\right)\vee \Delta_{l_{k}}\left(k\right)\right]\text{,}\Lambda_{l_{\bar{\emptyset }\left(k\right)}}\left(\Xi \right)=\underset{k\in \Omega }{\wedge }\left[\Lambda_{l_{\emptyset }}\left(\Xi ,k\right)\wedge \Lambda_{l_{k}}\left(k\right)\right]\text{,}\\ \Delta_{l_{\underline{\emptyset }\left(k\right)}}\left(\Xi \right)=\underset{k\in \Omega }{\wedge }\left[\Delta_{l_{\emptyset }}\left(\Xi ,k\right)\wedge \Delta_{l_{k}}\left(k\right)\right]\text{,}\Lambda_{l_{\underline{\emptyset }\left(k\right)}}\left(\Xi \right)=\underset{k\in \Omega }{\vee }\left[\Lambda_{l_{\emptyset }}\left(\Xi ,k\right)\vee \Lambda_{l_{k}}\left(k\right)\right]\text{,}\\ \nabla_{l_{\bar{\emptyset }\left(k\right)}}\left(\Xi \right)=\underset{k\in \Omega }{\vee }\left[\nabla_{l_{\emptyset }}\left(\Xi ,k\right)\vee \nabla_{l_{k}}\left(k\right)\right]\text{,}\nabla_{l_{\underline{\emptyset }\left(k\right)}}\left(\Xi \right)=\underset{k\in \Omega }{\wedge }\left[\nabla_{l_{\emptyset }}\left(\Xi ,k\right)\wedge \nabla_{l_{k}}\left(k\right)\right]\text{,} \end{array}$$

such that

(47) $$\begin{array}{c} 0<\left(\max\;\left(\Delta_{l_{\bar{\emptyset }\left(k\right)}}\left(\Xi \right)\right)\right)+\left(\min\;\left(\Lambda_{l_{\bar{\emptyset }\left(k\right)}}\left(\Xi \right)\right)\right)+\left(\min\;\left(\nabla_{l_{\bar{\emptyset }\left(k\right)}}\left(\Xi \right)\right)\right)\leq 3\text{,} \end{array}$$

and

(48) $$\begin{array}{c} 0<\left(\left.\min\;\left(\Delta_{l_{\underline{\emptyset }\left(k\right)}}\right.\left(\Xi \right.\right)\right)+\left(\max\;\left(\Lambda_{l_{\underline{\emptyset }\left(k\right)}}\left(\Xi \right)\right)\right)+\left(\max\;\left(\Lambda_{l_{\underline{\emptyset }\left(k\right)}}\left(\Xi \right)\right)\right)\leq 3\text{.} \end{array}$$

As $\left(\bar{\emptyset }\left(𝕜\right),\underline{\emptyset }\left(𝕜\right)\right)$ are SV − NHFRS, then $\bar{\emptyset }\left(𝕜\right),\underline{\emptyset }\left(𝕜\right):SV-NHFRS\left(\Omega \right)⟶SV-NHFRS\left(\Omega \right)$ are operators. The pair

(49) $$\begin{array}{c} \emptyset \left(k\right)=\left(\underline{\emptyset }\left(k\right),\bar{\emptyset }\left(k\right)\right)=\left\{\langle \begin{array}{c} \Xi ,\left(\Delta_{l_{\underline{\emptyset }\left(k\right)}}\left(\Xi \right),\Lambda_{l_{\underline{\emptyset }\left(k\right)}}\left(\Xi \right),\nabla_{l_{\underline{\emptyset }\left(k\right)}}\left(\Xi \right)\right),\\ \Xi ,\left(\Delta_{l_{\bar{\emptyset }\left(k\right)}}\left(\Xi \right),\Lambda_{l_{\bar{\emptyset }\left(k\right)}}\left(\Xi \right),\nabla_{l_{\bar{\emptyset }\left(k\right)}}\left(\Xi \right)\right) \end{array}\rangle \left|\Xi \right.\in k\right\}, \end{array}$$

will be called the SV-neutrosophic hesitant fuzzy rough set. For simplicity,

(50) $$\begin{array}{c} \emptyset \left(k\right)=\left\{\begin{array}{c} \langle \Xi ,\left(\Delta_{l_{\underline{\emptyset }\left(k\right)}}\left(\Xi \right),\Lambda_{l_{\underline{\emptyset }\left(k\right)}}\left(\Xi \right),\nabla_{l_{\underline{\emptyset }\left(k\right)}}\left(\Xi \right)\right),\\ (\left(\Delta_{l_{\bar{\emptyset }\left(k\right)}}\left(\Xi \right),\Lambda_{l_{\bar{\emptyset }\left(k\right)}}\left(\Xi \right),\nabla_{l_{\bar{\emptyset }\left(k\right)}}\left(\Xi \right)\right)\rangle \left|\Xi \right.\in k \end{array}\right\}, \end{array}$$

is described as

(51) $$\begin{array}{c} \emptyset \left(k\right)=\left(\left(\underline{\Delta },\underline{\Lambda },\underline{\nabla }\right),\left(\bar{\Delta },\bar{\Lambda },\bar{\nabla }\right)\right), \end{array}$$

and is called SV-NHFRSs.

**Definition 13 .**

Let $\emptyset \left({𝕜}_{1}\right)=\left(\underline{\emptyset }\left({𝕜}_{1}\right),\bar{\emptyset }\left({𝕜}_{1}\right)\right)$ and $\emptyset \left({𝕜}_{2}\right)=\left(\underline{\emptyset }\left({𝕜}_{2}\right),\bar{\emptyset }\left({𝕜}_{2}\right)\right)$ be two SV-NHFRSs. Then

- $\emptyset \left({𝕜}_{1}\right)\cup \emptyset \left({𝕜}_{2}\right)=\left\{\left(\underline{\emptyset }\left({𝕜}_{1}\right)\cup \underline{\emptyset }\left({𝕜}_{2}\right)\right),\left(\bar{\emptyset }\left({𝕜}_{1}\right)\cup \bar{\emptyset }\left({𝕜}_{2}\right)\right)\right\}$
- $\emptyset \left({𝕜}_{1}\right)\cap \emptyset \left({𝕜}_{2}\right)=\left\{\left(\underline{\emptyset }\left({𝕜}_{1}\right)\cap \underline{\emptyset }\left({𝕜}_{2}\right)\right),\left(\bar{\emptyset }\left({𝕜}_{1}\right)\cap \bar{\emptyset }\left({𝕜}_{2}\right)\right)\right\}$

**Definition 14 .**

Let $\emptyset \left({𝕜}_{1}\right)=\left(\underline{\emptyset }\left({𝕜}_{1}\right),\bar{\emptyset }\left({𝕜}_{1}\right)\right)$ and $\emptyset \left({𝕜}_{2}\right)=\left(\underline{\emptyset }\left({𝕜}_{2}\right),\bar{\emptyset }\left({𝕜}_{2}\right)\right)$ be two SV-NHFRSs. Then

- $\emptyset \left({𝕜}_{1}\right)⊕\emptyset \left({𝕜}_{2}\right)=\left\{\left(\underline{\emptyset }\left({𝕜}_{1}\right)⊕\underline{\emptyset }\left({𝕜}_{2}\right)\right),\left(\bar{\emptyset }\left({𝕜}_{1}\right)⊕\bar{\emptyset }\left({𝕜}_{2}\right)\right)\right\}$
- $\emptyset \left({𝕜}_{1}\right)⊗\emptyset \left({𝕜}_{2}\right)=\left\{\left(\underline{\emptyset }\left({𝕜}_{1}\right)⊗\underline{\emptyset }\left({𝕜}_{2}\right)\right),\left(\bar{\emptyset }\left({𝕜}_{1}\right)⊗\bar{\emptyset }\left({𝕜}_{2}\right)\right)\right\}$
- $\emptyset \left({𝕜}_{1}\right)\subseteq \emptyset \left({𝕜}_{2}\right)=\left\{\left(\underline{\emptyset }\left({𝕜}_{1}\right)\subseteq \underline{\emptyset }\left({𝕜}_{2}\right)\right)\right)$ and $\left(\left(\bar{\emptyset }\left({𝕜}_{1}\right)\subseteq \bar{\emptyset }\left({𝕜}_{2}\right)\right)\right\}$
- $\left.\Delta \emptyset \left({𝕜}_{1}\right)=\left(\Delta \right.\underline{\emptyset }\left({𝕜}_{1}\right.\right)$, $\left.\Delta \bar{\emptyset }\left({𝕜}_{1}\right)\right)$ for Δ ≥ 1
- $\left(\emptyset \left({𝕜}_{1}\right){\left(\text{⁣}\right)}^{\Delta }=\left(\left(\underline{\emptyset }\right.\right.\left({𝕜}_{1}\right){\left(\text{⁣}\right)}^{\Delta }\right.$, $\left(\bar{\emptyset }\left({𝕜}_{1}\right){\left(\text{⁣}\right)}^{\Delta }\right)$ for Δ ≥ 1
- ${\left({𝕜}_{1}\right)}^{c}=\left(\underline{\emptyset }\right.{\left({𝕜}_{1}\right)}^{c}$, $\bar{\emptyset }\left({𝕜}_{1}{\left(\text{⁣}\right)}^{c}\right)$, where $\underline{\emptyset }{\left({𝕜}_{1}\right)}^{c}$ and $\bar{\emptyset }{\left({𝕜}_{1}\right)}^{c}$ shows the aggregate of SV-neutrosophic fuzzy rough operators $\underline{\emptyset }\left({𝕜}_{1}\right)$ and $\bar{\emptyset }\left({𝕜}_{1}\right)$, that is, $\underline{\emptyset }{\left({𝕜}_{1}\right)}^{c}=\left(\nabla_{\ell_{\underline{\emptyset }\left(𝕜\right)}},\Lambda_{\ell_{\underline{\emptyset }\left(𝕜\right)}},\Delta_{\ell_{\underline{\emptyset }\left(𝕜\right)}}\right)$
- ∅(*𝕜*_1_) = ∅(*𝕜*_2_) iff $\underline{\emptyset }\left({𝕜}_{1}\right)=\underline{\emptyset }\left({𝕜}_{2}\right)$ and $\bar{\emptyset }\left({𝕜}_{1}\right)=\bar{\emptyset }\left({𝕜}_{2}\right)$

The score function is used to compare/rank two or more SV-NHFRNs. The SV-NHFRNs has the greater score value are said to be superior SV-NHFRNs. When the score values are equal, we will use the accuracy function.

**Definition 15 .**

The score function for SV-NHFRNs

(52) $$\begin{array}{c} \emptyset \left(k\right)=\left(\underline{\emptyset }\left(k\right),\bar{\emptyset }\left(k\right)\right)=\left(\left(\underline{\Delta },\underline{\Lambda },\underline{\nabla }\right),\left(\bar{\nabla },\bar{\Lambda },\bar{\Delta }\right)\right), \end{array}$$

is given as

(53) $$\begin{array}{c} \Delta \left(\emptyset \left(k\right)\right)=\frac{1}{6}\left(\begin{array}{c} 3+\left(\begin{array}{c} \frac{1}{Z_{\underline{textfranc}}}\underset{\underline{\mu_{l_{\tau }}}\in \Delta_{l_{\underline{\emptyset }\left(k\right)}}}{\sum }\left\{\underline{\mu_{l_{\tau }}}\right\}+\\ \frac{1}{Y_{\bar{\Omega }}}\sum \bar{\mu_{l_{\tau }}}\in \Delta_{l_{\bar{\emptyset }\left(k\right)}}\left\{\bar{\mu_{l_{\tau }}}\right\} \end{array}\right)-\left(\begin{array}{c} \frac{1}{M_{\underline{textfranc}}}\underset{\underline{\nu_{l_{\tau }}}\in \Lambda_{l_{\underline{\emptyset }\left(k\right)}}}{\sum }\left(\underline{\nu_{l_{\tau }}}\right)+\\ \frac{1}{V_{\bar{\Omega }}}\underset{\bar{\nu_{l_{\tau }}}\in \Lambda_{l_{\bar{\emptyset }\left(k\right)}}}{\sum }\left(\bar{\nu_{l_{\tau }}}\right) \end{array}\right)-\\ \left(\begin{array}{c} \frac{1}{U_{\underline{textfranc}}}\underset{\underline{\Pi_{l_{\tau }}}\in \nabla_{l_{\underline{\emptyset }\left(k\right)}}}{\sum }\left\{\underline{\lambda_{l_{\tau }}}\right\}+\\ \;\frac{1}{\delta_{\bar{\Omega }}}\underset{\bar{\Pi_{l_{\tau }}}\in \nabla_{l_{\bar{\emptyset }\left(k\right)}}}{\sum }\left(\bar{\lambda_{l_{\tau }}}\right) \end{array}\right) \end{array}\right)\text{.} \end{array}$$

The accuracy function for SV-NHFRNs

(54) $$\begin{array}{c} \emptyset \left(k\right)=\left(\underline{\emptyset }\left(k\right),\bar{\emptyset }\left(k\right)\right)=\left(\left(\underline{\Delta },\underline{\Lambda },\underline{\nabla }\right),\left(\bar{\nabla },\bar{\Lambda },\bar{\Delta }\right)\right), \end{array}$$

is given as

(55) $$\begin{array}{c} AC\emptyset \left(k\right)=\frac{1}{6}\left(\begin{array}{c} \frac{1}{Z_{\underline{\Omega }}}\underset{\underline{\mu_{l_{\tau }}}\in \Delta_{l_{\underline{\emptyset }\left(k\right)}}}{\sum }\left(\underline{\mu_{l_{\tau }}}\right)+\\ \frac{1}{Y_{\bar{\Omega }}}\underset{\bar{\mu_{l_{\tau }}}\in \Delta_{l_{\bar{\emptyset }\left(k\right)}}}{\sum }\left(\bar{\mu_{l_{\tau }}}\right)+\\ \frac{1}{M_{\underline{\Omega }}}\underset{\underline{\nu_{l_{\tau }}}\in \Lambda_{l_{\underline{\emptyset }\left(k\right)}}}{\sum }\left(\underline{\nu_{l_{\tau }}}\right)+\\ \frac{1}{V_{\bar{\Omega }}}\underset{\bar{\nu_{l_{\tau }}}\in \Lambda_{l_{\bar{\emptyset }\left(k\right)}}}{\sum }\left(\bar{\nu_{l_{\tau }}}\right)+\\ \begin{array}{c} \frac{1}{U_{\underline{\Omega }}}\underset{\underline{\lambda_{l_{\tau }}}\in \nabla_{l_{\underline{\emptyset }\left(k\right)}}}{\sum }\left\{\underline{\lambda_{l_{\tau }}}\right\}+\\ \frac{1}{\delta_{\bar{\Omega }}}\underset{\bar{\lambda_{l_{\tau }}}\in \nabla_{l_{\bar{\emptyset }\left(k\right)}}}{\sum }\left(\bar{\lambda_{l_{\tau }}}\right) \end{array} \end{array}\right)\text{,} \end{array}$$

where *Z*_Ω_, *Y*_Ω_, *M*_Ω_, *V*_Ω_, *U*_Ω_, and *δ*_Ω_ represent the number of elements in ∇_*ℓ*_♭__, Λ_*ℓ*_♭__, and Δ_*ℓ*_♭__, respectively.

**Definition 16 .**

Suppose $\emptyset \left({𝕜}_{1}\right)=\left(\underline{\emptyset }\left({𝕜}_{1}\right),\bar{\emptyset }\left({𝕜}_{1}\right)\right)$ and $\emptyset \left({𝕜}_{2}\right)=\left(\underline{\emptyset }\left({𝕜}_{2}\right),\bar{\emptyset }\left({𝕜}_{2}\right)\right)$ are two SV-NHFNs. Then

(i) If Δ(∅(*𝕜*_1_)) > Δ(∅(*𝕜*_2_)), then ∅(*𝕜*_1_) > ∅(*𝕜*_2_)
(ii) If Δ(∅(*𝕜*_1_))≺Δ(∅(*𝕜*_2_)), then ∅(*𝕜*_1_)≺∅(*𝕜*_2_)
(iii) If Δ(∅(*𝕜*_1_)) = Δ(∅(*𝕜*_2_)), then
  - If **AC**∅(*𝕜*_1_) > **AC**∅(*𝕜*_2_) then ∅(*𝕜*_1_) > ∅(*𝕜*_2_)
  - If **AC**∅(*𝕜*_1_)≺**AC**∅(*𝕜*_2_) then ∅(*𝕜*_1_)≺∅(*𝕜*_2_)
  - If **AC**∅(*𝕜*_1_) = **AC**∅(*𝕜*_2_) then ∅(*𝕜*_1_) = ∅(*𝕜*_2_)

## 4. SV-Neutrosophic Hesitant Fuzzy Rough Aggregation Operators

We introduce a novel concept of SV-NHF rough aggregation operators in this article by combining rough sets and SV-NHF aggregation operators to produce the aggregation concepts SV-NHFRWA, SV-NHFROWA, and SV-NHFRHWA. These ideas' fundamental features are addressed in this article.

### 4.1. Single-Valued Neutrosophic Hesitant Fuzzy Rough Weighted Averaging Operator

**Definition 17 .**

Consider the set of values $\emptyset \left({𝕜}_{\tau }\right)=\left(\underline{\emptyset }\left({𝕜}_{\tau }\right),\bar{\emptyset }\left({𝕜}_{\tau }\right)\right)\left(\tau =1,2,3,4,\ldots ,\overset{ˇ}{n}\right)$ of SV-NHFRNs with weight vector $M={\left(M_{1},M_{2},\ldots M_{\overset{ˇ}{n}}\right)}^{\delta }$ such that ${⊕}_{\tau =1}^{\overset{ˇ}{n}}M_{\tau }=1$ and 0 ≤ *M*_*τ*_ ≤ 1. The SV-NHFRWA operator is determined as

(56) $$\begin{array}{c} SV-NHFWA\left(\emptyset \left(k_{1}\right),\emptyset \left(k_{2}\right),\ldots ,\emptyset \left(k_{\overset{ˇ}{n}}\right)\right)=\left({⊕}_{\tau =1}^{\overset{ˇ}{n}}M_{\tau }\underline{\emptyset }\left(k_{\tau }\right),{⊕}_{\tau =1}^{\overset{ˇ}{n}}M_{\tau }\bar{\emptyset }\left(k_{\tau }\right)\right)\text{.} \end{array}$$

**Theorem 1 .**

Let $\emptyset \left({𝕜}_{\tau }\right)=\left(\underline{\emptyset }\left({𝕜}_{\tau }\right),\bar{\emptyset }\left({𝕜}_{\tau }\right)\right)\left(\tau =1,2,3,4,\ldots \overset{ˇ}{n}\right)$ be the set of values of SV− NHFRNs with weight vector $M={\left(M_{1},M_{2},\ldots M_{\overset{ˇ}{n}}\right)}^{\delta }$. Then the SV− NHFRWA operator is defined as

(57) $$\begin{array}{c} SV-NHFWA\left(\emptyset \left(k_{1}\right),\emptyset \left(k_{2}\right),\ldots \emptyset \left(k_{\overset{ˇ}{n}}\right)\right)=\left(\overset{\overset{ˇ}{n}}{\underset{\tau =1}{⊕}}M_{\tau }\underline{\emptyset }\left(k_{\tau }\right),\overset{\overset{ˇ}{n}}{\underset{\tau =1}{⊕}}M_{\tau }\bar{\emptyset }\left(k_{\tau }\right)\right)\text{,}\\ =\left[\begin{array}{c} \left(\begin{array}{c} \underset{\underline{\mu_{l_{\tau }}}\in \Delta_{l_{\underline{\emptyset }\left(k\right)}}}{\cup }\left(\left(1-\overset{\overset{ˇ}{n}}{\underset{\tau =1}{⊕}}{\left(1-\underline{\mu_{l_{\tau }}}\right)}^{M_{\tau }}\right)\right),\\ \underset{\underline{\lambda_{l_{\tau }}}\in \Lambda_{l_{\underline{\emptyset }\left(k\right)}}}{\cup }\left(\overset{\overset{ˇ}{n}}{\underset{\tau =1}{⊕}}{\left(\underline{\lambda_{l_{\tau }}}\right)}^{M_{\tau }}\right),\\ \;\underset{\underline{\nu_{l_{\tau }}}\in \nabla_{l_{\underline{\emptyset }\left(k\right)}}}{\cup }\left(\overset{\overset{ˇ}{n}}{\underset{\tau =1}{⊕}}{\left(\underline{\nu_{l_{\tau }}}\right)}^{M_{\tau }}\right) \end{array}\right)\\ \left(\begin{array}{c} \underset{\bar{\mu_{l_{\tau }}}\in \Delta_{l_{\bar{\emptyset }\left(k\right)}}}{\cup }\left(\left(1-\overset{\overset{ˇ}{n}}{\underset{\tau =1}{⊕}}{\left(1-\left(\bar{\mu_{l_{\tau }}}\right)\right)}^{M_{\tau }}\right)\right),\\ \underset{\bar{\lambda_{l_{\tau }}}\in \Lambda_{l_{\bar{\emptyset }\left(k\right)}}}{\cup }\left(\overset{\overset{ˇ}{n}}{\underset{\tau =1}{⊕}}{\left(\bar{\nu_{l_{\tau }}}\right)}^{M_{\tau }}\right)\\ \;\underset{\bar{\nu_{l_{\tau }}}\in \nabla_{l_{\bar{\emptyset }\left(k\right)}}}{\cup }\left(\overset{\overset{ˇ}{n}}{\underset{\tau =1}{⊕}}{\left(\bar{\nu_{l_{\tau }}}\right)}^{M_{\tau }}\right) \end{array}\right) \end{array}\right]\text{.} \end{array}$$

**Proof**

Applying mathematical induction to proof. Applying the operational law, it follows that

(58) $$\begin{array}{c} \emptyset \left(k_{1}\right)⊕\emptyset \left(k_{2}\right)=\left[\underline{\emptyset }\left(k_{1}\right)⊕\underline{\emptyset }\left(k_{2}\right),\bar{\emptyset }\left(k_{1}\right)⊕\bar{\emptyset }\left(k_{2}\right)\right], \end{array}$$

and

(59) $$\begin{array}{c} \Delta \emptyset \left(k_{1}\right)=\left(\Delta \underline{\emptyset }\left(k_{1}\right),\Delta \bar{\emptyset }\left(k_{1}\right)\right)\text{.} \end{array}$$

If $\overset{ˇ}{n}=2$, then

(60) $$\begin{array}{c} SV-NHFRWA\left(\emptyset \left(k_{1}\right),\emptyset \left(k_{2}\right)\right)\text{,}\\ =\left({⊕}_{\tau =1}^{2}M_{\tau }\underline{\emptyset }\left(k_{\tau }\right),{⊕}_{\tau =1}^{2}M_{\tau }\bar{\emptyset }\left(k_{\tau }\right)\right)\text{,}\\ =\left(\begin{array}{c} \left(\begin{array}{c} \underset{\underline{\mu_{l_{\tau }}}\in \Delta_{l_{\%\underline{\emptyset }\left(k\right)}}}{\cup }\left(\left(1-\overset{2}{\underset{\tau =1}{⊗}}{\left(1-\underline{\mu_{l_{\tau }}}\right)}^{M_{\tau }}\right)\right),\\ \underset{\underline{\lambda_{l_{\tau }}}\in \Lambda_{l_{\%\underline{\emptyset }\left(k\right)}}}{\cup }\left(\overset{2}{\underset{\tau =1}{⊗}}{\left(\underline{\lambda_{l_{\tau }}}\right)}^{M_{\tau }}\right),\\ \underset{\underline{\nu_{l_{\tau }}}\in \nabla_{l_{\%\underline{\emptyset }\left(k\right)}}}{\cup }\left(\overset{2}{\underset{\tau =1}{⊗}}{\left(\underline{\nu_{l_{\tau }}}\right)}^{M_{\tau }}\right) \end{array}\right)\\ \left(\begin{array}{c} \underset{\bar{\mu_{l_{\tau }}}\in \Delta_{l_{\%\bar{\emptyset }\left(k\right)}}}{\cup }\left(\left(1-\overset{2}{\underset{\tau =1\%}{⊗}}{\left(1-\bar{\mu_{l_{\tau }}}\right)}^{M_{\tau }}\right)\right),\\ \underset{\bar{\lambda_{l_{\tau }}}\in \Lambda_{l_{\%\bar{\emptyset }\left(k\right)}}}{\cup }\left(\overset{2}{\underset{\tau =1}{⊗}}{\left(\bar{\lambda_{l_{\tau }}}\right)}^{M_{\tau }}\right)\\ \underset{\bar{\nu_{l_{\tau }}}\in \nabla_{l_{\%\bar{\emptyset }\left(k\right)}}}{\cup }\left(\overset{2}{\underset{\tau =1}{⊗}}{\left(\bar{\nu_{l_{\tau }}}\right)}^{M_{\tau }}\right) \end{array}\right) \end{array}\right)\text{.} \end{array}$$

Hence, the result is correct for $\overset{ˇ}{n}=2$. Let it be correct for $\overset{ˇ}{n}=k$, that is,

(61) $$\begin{array}{c} SV-NHFRWA\left(\emptyset \left(k_{1}\right),\emptyset \left(k_{2}\right),\ldots \emptyset \left(k_{k}\right)\right)\text{,}\\ =\left({⊕}_{\tau =1}^{k}M_{\tau }\underline{\emptyset }\left(k_{\tau }\right),{⊕}_{\tau =1}^{k}M_{\tau }\bar{\emptyset }\left(k_{\tau }\right)\right)\text{,}\\ =\left(\begin{array}{c} \left(\begin{array}{c} \underset{\underline{\mu_{l_{\tau }}}\in \Delta_{l_{\%\underline{\emptyset }\left(k\right)}}}{\cup }\left(\left(1-\overset{k}{\underset{\tau =1}{⊗}}{\left(1-\underline{\mu_{l_{\tau }}}\right)}^{M_{\tau }}\right)\right),\\ \underset{\underline{\lambda_{l_{\tau }}}\in \Lambda_{l_{\underline{\emptyset }\left(k\right)}}}{\cup }\left(\overset{k}{\underset{\tau =1}{⊗}}{\left(\underline{\lambda_{l_{\tau }}}\right)}^{M_{\tau }}\right),\\ \underset{\underline{\nu_{l_{\tau }}}\in \nabla_{l_{\%\underline{\emptyset }\left(k\right)}}}{\cup }\left(\overset{k}{\underset{\tau =1}{⊗}}{\left(\underline{\nu_{l_{\tau }}}\right)}^{M_{\tau }}\right) \end{array}\right)\\ \left(\begin{array}{c} \underset{\bar{\mu_{l_{\tau }}}\in \Delta_{l_{\%\bar{\emptyset }\left(k\right)}}}{\cup }\left(\left(1-\overset{k}{\underset{\tau =1\%}{⊗}}{\left(1-\bar{\mu_{l_{\tau }}}\right)}^{M_{\tau }}\right)\right),\\ \underset{\bar{\lambda_{l_{\tau }}}\in \Lambda_{l_{\%\bar{\emptyset }\left(k\right)}}}{\cup }\left(\overset{k}{\underset{\tau =1}{⊗}}{\left(\bar{\lambda_{l_{\tau }}}\right)}^{M_{\tau }}\right)\\ \underset{\bar{\nu_{l_{\tau }}}\in \nabla_{l_{\%\bar{\emptyset }\left(k\right)}}}{\cup }\left(\overset{k}{\underset{\tau =1}{⊗}}{\left(\bar{\nu_{l_{\tau }}}\right)}^{M_{\tau }}\right) \end{array}\right) \end{array}\right)\text{.} \end{array}$$

Now, we have to show that it is correct for $\overset{ˇ}{n}=k+1$, then we have

(62) $$\begin{array}{c} SV-NHFRWA\left(\emptyset \left(k_{1}\right),\emptyset \left(k_{2}\right),\ldots \emptyset \left(k_{k+1}\right)\right)\text{,}\\ =\left(\begin{array}{c} \left({⊕}_{\tau =1}^{k}M_{\tau }\underline{\emptyset }\left(k_{\tau }\right)⊕M_{k+1}\underline{\emptyset }\left(k_{k+1}\right)\right)\text{,}\\ \left({⊕}_{\tau =1}^{k}M_{\tau }\bar{\emptyset }\left(k_{\tau }\right)⊕M_{k+1}\bar{\emptyset }\left(k_{k+1}\right)\right) \end{array}\right)\text{,}\\ =\left(\begin{array}{c} \left(\begin{array}{c} \underset{\underline{\mu_{l_{\tau }}}\in \Delta_{l_{\%\underline{\emptyset }\left(k\right)}}}{\cup }\left(\left(1-\overset{k+1}{\underset{\%\tau =1}{⊗}}{\left(1-\underline{\mu_{l_{\tau }}}\right)}^{M_{\tau }}\right)\right),\\ \underset{\underline{\lambda_{l_{\tau }}}\in \Lambda_{l_{\%\underline{\emptyset }\left(k\right)}}}{\cup }\left(\overset{k+1}{\underset{\tau =1}{⊗}}{\left(\underline{\lambda_{l_{\tau }}}\right)}^{M_{\tau }}\right)\\ \underset{\underline{\nu_{l_{\tau }}}\in \nabla_{l_{\%\underline{\emptyset }\left(k\right)}}}{\cup }\left(\overset{k+1}{\underset{\tau =1}{⊗}}{\left(\underline{\nu_{l_{\tau }}}\right)}^{M_{\tau }}\right) \end{array}\right)\\ \left(\begin{array}{c} \underset{\bar{\mu_{l_{\tau }}}\in \Delta_{l_{\%\bar{\emptyset }\left(k\right)}}}{\cup }\left(\left(1-\overset{k+1}{\underset{\tau =1}{⊗}}{\left(1-\bar{\mu_{l_{\tau }}}\right)}^{M_{\tau }}\right)\right),\\ \underset{\bar{\lambda_{l_{\tau }}}\in \Lambda_{l_{\bar{\emptyset }\left(k\right)}}}{\cup }\left(\overset{k+1}{\underset{\tau =1}{⊗}}{\left(\bar{\lambda_{l_{\tau }}}\right)}^{M_{\tau }}\right),\\ \underset{\bar{\nu_{l_{\tau }}}\in \nabla_{l_{\%\bar{\emptyset }\left(k\right)}}}{\cup }\left(\overset{k+1}{\underset{\tau =1}{⊗}}{\left(\bar{\nu_{l_{\tau }}}\right)}^{M_{\tau }}\right) \end{array}\right) \end{array}\right)\text{.} \end{array}$$

As a result, the conclusion is correct for $\overset{ˇ}{n}=k+1$. Hence, the result is correct for all $\overset{ˇ}{n}\geq 1$.

From the above analysis $\underline{\emptyset }\left(𝕜\right)$ and $\bar{\emptyset }\left(𝕜\right)$ are SV-NHFRNs. So ${⊕}_{\tau =1}^{k}M_{\tau }\underline{\emptyset }\left({𝕜}_{\tau }\right)$ and ${⊕}_{\tau =1}^{k}M_{\tau }\bar{\emptyset }\left({𝕜}_{\tau }\right)$ are also SV-NHFRNs.

Therefore, SV-NHFRWA $\left(\emptyset \left({𝕜}_{1}\right),\emptyset \left({𝕜}_{2}\right),\ldots \emptyset \left({𝕜}_{\overset{ˇ}{n}}\right)\right)$ is a SV-NHFRN under SV-NHF approximation space (£, ∅).

**Theorem 2 .**

Consider the set of values $\emptyset \left({𝕜}_{\tau }\right)=\left(\underline{\emptyset }\left({𝕜}_{\tau }\right),\bar{\emptyset }\left({𝕜}_{\tau }\right)\right)\left(\tau =1,2,3,4,\ldots ,\overset{ˇ}{n}\right)$ of SV-NHFRNs with $M={\left(M_{1},M_{2},\ldots M_{\overset{ˇ}{n}}\right)}^{\delta }$ such that ${⊕}_{\tau =1}^{\overset{ˇ}{n}}M_{\tau }=1$ and 0 ≤ *M*_*τ*_ ≤ 1.

Then SV-NHFRWA operator satisfies the following properties:

(1) Idempotency: If ∅(*𝕜*_*τ*_) = *ℑ*(*𝕜*) for $\left(\tau =1,2,3,4,\ldots ,\overset{ˇ}{n}\right)$, where
  (63) $$\begin{array}{c} I\left(k\right)=\left(\underline{I}\left(k\right),\bar{I}\left(k\right)\right)=\left(\left(\underline{a_{l\left(\Upsilon \right)}},\underline{b_{l\left(\Upsilon \right)}},\underline{c_{l\left(\Upsilon \right)}}\right),\left({\bar{a}}_{l\left(\Upsilon \right)},\bar{b_{l\left(\Upsilon \right)}},\bar{c_{l\left(\Upsilon \right)}}\right)\right)\text{,} \end{array}$$
then
  (64) $$\begin{array}{c} SV-NHFRWA\left(\emptyset \left(k_{1}\right),\emptyset \left(k_{2}\right),\ldots \emptyset \left(k_{\overset{ˇ}{n}}\right)\right)=I\left(k\right)\text{.} \end{array}$$
(2) Boundedness**:** Let ${\left(\emptyset \left(𝕜\right)\right)}^{-}=\left(\min_{\tau }\underline{\emptyset }\left({𝕜}_{\tau }\right),\max_{\tau }\bar{\emptyset }\left({𝕜}_{\tau }\right)\right)$ and ${\left(\emptyset \left(𝕜\right)\right)}^{+}=\left(\max_{\tau }\underline{\emptyset }\left({𝕜}_{\tau }\right),\min_{\tau }\bar{\emptyset }\left({𝕜}_{\tau }\right)\right)$. Then
  (65) $$\begin{array}{c} {\left(\emptyset \left(k\right)\right)}^{-}\leq SV-NHFRWA\left(\emptyset \left(k_{1}\right),\emptyset \left(k_{2}\right),\ldots ,\emptyset \left(k_{\overset{ˇ}{n}}\right)\right)\leq {\left(\emptyset \left(k\right)\right)}^{+}\text{.} \end{array}$$
(3) Monotonicity**:** Suppose $ℑ\left(𝕜\right)=\left(\underline{\emptyset }\left({𝕜}_{\tau }\right),\bar{ℑ}\left({𝕜}_{\tau }\right)\right)\left(\tau =\tau ,2,\ldots ,\overset{ˇ}{n}\right)$ is another set of values of SV-NHFRNs such that $\underline{ℑ}\left({𝕜}_{\tau }\right)\leq \underline{\emptyset }\left({𝕜}_{\tau }\right)$ and $\bar{ℑ}\left({𝕜}_{\tau }\right)\leq \bar{\emptyset }\left({𝕜}_{\tau }\right)$. Then
  (66) $$\begin{array}{c} SV-NHFRWA\left(I\left(k_{1}\right),I\left(k_{2}\right),\ldots ,I\left(k_{\overset{ˇ}{n}}\right)\right)\leq SV-NHFRWA\left(\emptyset \left(k_{1}\right),\emptyset \left(k_{2}\right),\ldots \emptyset \left(k_{\overset{ˇ}{n}}\right)\right)\text{.} \end{array}$$
(4) Shift invariance**:** Consider another SV-NHFRN
  (67) $$\begin{array}{c} I\left(k\right)=\left(\underline{I}\left(k\right),\bar{I}\left(k\right)\right)=\left(\left(\underline{a_{l\left(\Upsilon \right)}},\underline{b_{l\left(\Upsilon \right)}},\underline{c_{l\left(\Upsilon \right)}}\right),\left({\bar{a}}_{l\left(\Upsilon \right)},\bar{b_{l\left(\Upsilon \right)}},\bar{c_{l\left(\Upsilon \right)}}\right)\right)\text{,} \end{array}$$
then
  (68) $$\begin{array}{c} SV-NHFRWA\left(\emptyset \left(k_{1}\right)⊕I\left(k\right),\emptyset \left(k_{2}\right)⊕I\left(k\right),\ldots ,\emptyset \left(k_{\overset{ˇ}{n}}\right)⊕I\left(k\right)\right)\text{,}\\ =SV-NHFRWA\left(\emptyset \left(k_{1}\right),\emptyset \left(k_{2}\right),\ldots \emptyset \left(k_{\overset{ˇ}{n}}\right)\right)⊕I\left(k\right)\text{.} \end{array}$$
(5) Homogeneity**:** For any real number Δ > 0,
  (69) $$\begin{array}{c} SV-NHFRWA\left(\Delta \emptyset \left(k_{1}\right),\Delta \emptyset \left(k_{2}\right),\ldots ,\Delta \emptyset \left(k_{\overset{ˇ}{n}}\right)\right)=\Delta \cdot SV-NHFRWA\left(\emptyset \left(k_{1}\right),\emptyset \left(k_{2}\right),\ldots ,\emptyset \left(k_{\overset{ˇ}{n}}\right)\right)\text{.} \end{array}$$
(6) Commutativity**:** Suppose $\emptyset^{\prime }\left({𝕜}_{\tau }\right)=\left(\underline{\emptyset }\left({𝕜}_{\tau }\right),\bar{\emptyset^{\prime }}\left({𝕜}_{\tau }\right)\right)$ and $\emptyset \left({𝕜}_{\tau }\right)=\left(\underline{\emptyset }\left({𝕜}_{\tau }\right),\bar{\emptyset }\left({𝕜}_{\tau }\right)\right)$, $\left(\tau =1,2,3,4,\ldots ,\overset{ˇ}{n}\right)$ is a set of values of SV-NHFRNs.

Then

(70) $$\begin{array}{c} SV-NHFRWA\left(\emptyset \left(k_{1}\right),\emptyset \left(k_{2}\right),\ldots ,\emptyset \left(k_{\overset{ˇ}{n}}\right)\right)=SV-NHFRWA\left(\emptyset^{\prime }\left(k_{1}\right),\emptyset^{\prime }\left(k_{2}\right),\ldots ,\emptyset^{\prime }\left(k_{\overset{ˇ}{n}}\right)\right)\text{.} \end{array}$$

**Proof**

(1) **Idempotency:** As ∅(*𝕜*_*τ*_) = *ℑ*(*𝕜*) (for all $\tau =1,2,3,\ldots ,\overset{ˇ}{n}$) where
  (71) $$\begin{array}{c} I\left(k_{\tau }\right)=\left(\underline{I}\left(k\right),\bar{I}\left(k\right)\right)=\left(\left(\underline{a_{l\left(\Upsilon \right)}},\underline{b_{l\left(\Upsilon \right)}},\underline{c_{l\left(\Upsilon \right)}}\right),\left({\bar{a}}_{l\left(\Upsilon \right)},\bar{b_{l\left(\Upsilon \right)}},\bar{c_{l\left(\Upsilon \right)}}\right)\right)\text{.} \end{array}$$
It follows that
  (72) $$\begin{array}{c} SV-NHFRWA\left(\emptyset \left(k_{1}\right),\emptyset \left(k_{2}\right),\ldots ,\emptyset \left(k_{\overset{ˇ}{n}}\right)\right)\text{,}\\ =\left(\overset{\overset{ˇ}{n}}{\underset{\tau =1}{⊕}}M_{\tau }\underline{\emptyset }\left(k_{\tau }\right),\overset{\overset{ˇ}{n}}{\underset{\tau =1}{⊕}}M_{\tau }\bar{\emptyset }\left(k_{\tau }\right)\right)\text{,}\\ =\left[\begin{array}{c} \left(\begin{array}{c} \underset{\underline{\mu_{l_{\tau }}}\in \Delta_{l_{\underline{\emptyset }\left(k\right)}}}{\cup }\left(1-\overset{\overset{ˇ}{n}}{\underset{\tau =1}{⊕}}{\left(1-\underline{\mu_{l_{\tau }}}\right)}^{M_{\tau }}\right),\\ \underset{\underline{\lambda_{l_{\tau }}}\in \Lambda_{l_{\underline{\emptyset }\left(k\right)}}}{\cup }\left(\overset{\overset{ˇ}{n}}{\underset{\tau =1}{⊕}}{\left(\underline{\lambda_{l_{\tau }}}\right)}^{M_{\tau }}\right)\\ \underset{\underline{\nu_{l_{\tau }}}\in \nabla_{l_{\underline{\emptyset }\left(k\right)}}}{\cup }\left(\overset{\overset{ˇ}{n}}{\underset{\tau =1}{⊕}}{\left(\underline{\nu_{l_{\tau }}}\right)}^{M_{\tau }}\right) \end{array}\right)\\ \left(\begin{array}{c} \underset{\bar{\mu_{l_{\tau }}}\in \Delta_{l_{\bar{\emptyset }\left(k\right)}}}{\cup }\left(1-\overset{\overset{ˇ}{n}}{\underset{\tau =1}{⊕}}{\left(1-\bar{\mu_{l_{\tau }}}\right)}^{M_{\tau }}\right),\\ \underset{\bar{\lambda_{l_{\tau }}}\in \Lambda_{l_{\bar{\emptyset }\left(k\right)}}}{\cup }\left(\overset{\overset{ˇ}{n}}{\underset{\tau =1}{⊕}}{\left(\bar{\lambda_{l_{\tau }}}\right)}^{M_{\tau }}\right),\\ \underset{\bar{\nu_{l_{\tau }}}\in \nabla_{l_{\bar{\emptyset }\left(k\right)}}}{\cup }\left(\overset{\overset{ˇ}{n}}{\underset{\tau =1}{⊕}}{\left(\bar{\nu_{l_{\tau }}}\right)}^{M_{\tau }}\right) \end{array}\right) \end{array}\right]\text{.} \end{array}$$
For all *τ*, $\emptyset \left({𝕜}_{\tau }\right)=ℑ\left(𝕜\right)=\left(\underline{ℑ}\left(𝕜\right),\bar{ℑ}\left(𝕜\right)\right)=\left(\left(\underline{b_{\ell \left(\Upsilon \right)}},\underline{d_{\ell \left(\Upsilon \right)}}\right),\left({\bar{a}}_{\ell \left(\Upsilon \right)},\bar{b_{\ell \left(\Upsilon \right)}}\right)\right)$. Therefore,
  (73) $$\begin{array}{c} =\left[\begin{array}{c} \left(\begin{array}{c} \underset{\underline{b_{l\left(\Upsilon \right)}}\in \nabla_{l_{\underline{\emptyset }\left(k\right)}}}{\cup }\left(\left(1-\overset{\overset{ˇ}{n}}{\underset{\tau =1}{⊕}}{\left(1-\underline{b_{l\left(\Upsilon \right)}}\right)}^{M_{\tau }}\right)\right),\\ \underset{\underline{d_{l\left(\Upsilon \right)}}\in \Delta_{l_{\underline{\emptyset }\left(k\right)}}}{\cup }\left(\overset{\overset{ˇ}{n}}{\underset{\tau =1}{⊕}}{\left(\underline{d_{l\left(\Upsilon \right)}}\right)}^{M_{\tau }}\right) \end{array}\right)\\ \left(\begin{array}{c} \underset{\bar{a_{l\left(\Upsilon \right)}}\in \nabla_{l_{\bar{\emptyset }\left(k\right)}}}{\cup }\left(\left(1-\overset{\overset{ˇ}{n}}{\underset{\tau =1}{⊕}}{\left(1-\bar{a_{l\left(\Upsilon \right)}}\right)}^{M_{\tau }}\right)\right),\\ \underset{\bar{a_{l\left(\Upsilon \right)}}\in \Delta_{l_{\bar{\emptyset }\left(k\right)}}}{\cup }\left(\overset{\overset{ˇ}{n}}{\underset{\tau =1}{⊕}}{\left(\bar{b_{l\left(\Upsilon \right)}}\right)}^{M_{\tau }}\right) \end{array}\right) \end{array}\right]\text{,}\\ =\left[\left(1-\left(1-\underline{a_{l\left(\Upsilon \right)}}\right),\underline{b_{l\left(\Upsilon \right)}},\underline{c_{l\left(\Upsilon \right)}}\right),\left(1-\left(1-{\bar{a}}_{l\left(\Upsilon \right)}\right),{\bar{b}}_{l\left(\Upsilon \right)},{\bar{c}}_{l\left(\Upsilon \right)}\right)\right]\text{,}\\ =\left(\underline{I}\left(k\right),\bar{I}\left(k\right)\right)=I\left(k\right)\text{.} \end{array}$$
Hence, SV-NHFRWA $\left(\emptyset \left({𝕜}_{1}\right),\emptyset \left({𝕜}_{2}\right),\ldots \emptyset \left({𝕜}_{\overset{ˇ}{n}}\right)\right)=ℑ\left(𝕜\right)$.
(2) Boundedness: As
  (74) $$\begin{array}{c} {\left(\underline{\emptyset }\left(k\right)\right)}^{-}=\left[\begin{array}{c} \left(\min_{\tau }\left\{\underline{\mu_{l_{\tau }}}\right\},\max_{\tau }\left\{\underline{\lambda_{l_{\tau }}}\right\},\max_{\tau }\left\{\underline{\nu_{l_{\tau }}}\right\}\right)\text{,}\\ \left(\min_{\tau }\left\{\bar{\mu_{l_{\tau }}}\right\},\max_{\tau }\left\{{\bar{\lambda_{l}}}_{\tau }\right\},\max_{\tau }\left\{{\bar{\nu_{l}}}_{\tau }\right\}\right) \end{array}\right]\text{,}\\ {\left(\underline{\emptyset }\left(k\right)\right)}^{+}=\left[\begin{array}{c} \left(\max_{\tau }\left\{\underline{\mu_{l_{\tau }}}\right\},\min_{\tau }\left\{\underline{\lambda_{l_{\tau }}}\right\},\min_{\tau }\left\{\underline{\nu_{l_{\tau }}}\right\}\right)\text{,}\\ \left(\max_{\tau }\left\{\bar{\mu_{l_{\tau }}}\right\},\min_{\tau }\left\{{\bar{\lambda_{l}}}_{\tau }\right\},\min_{\tau }\left\{{\bar{\nu_{l}}}_{\tau }\right\}\right) \end{array}\right]\text{,} \end{array}$$
and
  (75) $$\begin{array}{c} \emptyset \left(k_{\tau }\right)=\left[\left(\underline{\Delta },\underline{\Lambda },\underline{\nabla }\right),\left(\bar{\Delta_{\tau }},{\bar{\Lambda }}_{\tau },{\bar{\nabla }}_{\tau }\right)\right]\text{.} \end{array}$$
To prove that
  (76) $$\begin{array}{c} {\left(\emptyset \left(k\right)\right)}^{-}\leq SV-NHFRWA\left(\emptyset \left(k_{1}\right),\emptyset \left(k_{2}\right),\ldots ,\emptyset \left(k_{\overset{ˇ}{n}}\right)\right)\leq {\left(\emptyset \left(k\right)\right)}^{+}\text{.} \end{array}$$
Since for each $\tau =1,2,3,\ldots ,\overset{ˇ}{n}$, it follows that
  (77) $$\begin{array}{c} \min_{\tau }\left\{\underline{\mu_{l_{\tau }}}\right\}\leq \left\{\underline{\mu_{l_{\tau }}}\right\}\leq \max_{\tau }\left\{\underline{\mu_{l_{\tau }}}\right\}\text{,}\\ \Leftrightarrow 1-\max_{\tau }\left\{\underline{\mu_{l_{\tau }}}\right\}\leq 1-\left\{\underline{\mu_{l_{\tau }}}\right\}\leq 1-\left\{\underline{\mu_{l_{\tau }}}\right\}\text{,}\\ \Leftrightarrow \overset{\overset{ˇ}{n}}{\underset{\tau =1}{⊕}}{\left(1-\max_{\tau }\left\{\underline{\mu_{l_{\tau }}}\right\}\right)}^{M_{\tau }}\leq \overset{\overset{ˇ}{n}}{\underset{\tau =1}{⊕}}{\left(1-\left\{\underline{\mu_{l_{\tau }}}\right\}\right)}^{M_{\tau }}\text{,}\\ \leq \overset{\overset{ˇ}{n}}{\underset{\tau =1}{⊕}}{\left(1-\min_{\tau }\left\{\underline{\mu_{l_{\tau }}}\right\}\right)}^{M_{\tau }}\text{,}\\ \Leftrightarrow \left(1-\max_{\tau }\left\{\underline{\mu_{l_{\tau }}}\right\}\right)\leq \overset{\overset{ˇ}{n}}{\underset{\tau =1}{⊕}}{\left(1-\left\{\underline{\mu_{l_{\tau }}}\right\}\right)}^{M_{\tau }}\text{,}\\ \leq \left(1-\min_{\tau }\left\{\underline{\mu_{l_{\tau }}}\right\}\right)\text{,}\\ \Leftrightarrow 1-\left(1-\min_{\tau }\left\{\underline{\mu_{l_{\tau }}}\right\}\right)\leq 1-\overset{\overset{ˇ}{n}}{\underset{\tau =1}{⊕}}{\left(1-\left\{\underline{\mu_{l_{\tau }}}\right\}\right)}^{M_{\tau }}\text{,}\\ \leq 1-\left(1-\max_{\tau }\left\{\underline{\mu_{l_{\tau }}}\right\}\right)\text{.} \end{array}$$
Hence,
  (78) $$\begin{array}{c} \min_{\tau }\left\{\underline{\mu_{l_{\tau }}}\right\}\leq 1-\overset{\overset{ˇ}{n}}{\underset{\tau =1}{⊕}}{\left(1-\left\{\underline{\mu_{l_{\tau }}}\right\}\right)}^{M_{\tau }}\leq \max_{\tau }\left\{\underline{\mu_{l_{\tau }}}\right\}\text{.} \end{array}$$
Next for each $\tau =1,2,3,\ldots ,\overset{ˇ}{n}$, we have
  (79) $$\begin{array}{c} \min_{\tau }\left\{\underline{\lambda_{l_{\tau }}}\right\}\leq \left\{\underline{\lambda_{l_{\tau }}}\right\}\leq \max_{\tau }\left\{\underline{\lambda_{l_{\tau }}}\right\}\text{,}\\ \Leftrightarrow \overset{\overset{ˇ}{n}}{\underset{\tau =1}{⊕}}{\left(\min_{\tau }\left\{\underline{\lambda_{l_{\tau }}}\right\}\right)}^{M_{\tau }}\leq \overset{\overset{ˇ}{n}}{\underset{\tau =1}{⊕}}{\left(\underline{\lambda_{l_{\tau }}}\right)}^{M_{\tau }}\text{,}\\ \leq \overset{\overset{ˇ}{n}}{\underset{\tau =1}{⊕}}{\left(\max_{\tau }\left\{\underline{\lambda_{l_{\tau }}}\right\}\right)}^{M_{\tau }}\text{.} \end{array}$$
This implies that
  (80) $$\begin{array}{c} \min_{\tau }\left\{\underline{\lambda_{l_{\tau }}}\right\}\leq \overset{\overset{ˇ}{n}}{\underset{\tau =1}{⊕}}{\left\{\underline{\lambda_{l_{\tau }}}\right\}}^{M_{\tau }}\leq \max_{\tau }\left\{\underline{\lambda_{l_{\tau }}}\right\}\text{.} \end{array}$$
Likewise, we can present that
  (81) $$\begin{array}{c} \min_{\tau }\left\{\bar{\mu_{l_{\tau }}}\right\}\leq \overset{\overset{ˇ}{n}}{\underset{\tau =1}{⊕}}{\left\{\bar{\mu_{l_{\tau }}}\right\}}^{M_{\tau }}\leq \max_{\tau }\left\{\bar{\mu_{l_{\tau }}}\right\}\text{,}\\ \min_{\tau }\left\{{\bar{\lambda_{l}}}_{\tau }\right\}\leq \overset{\overset{ˇ}{n}}{\underset{\tau =1}{⊕}}{\left\{{\bar{\lambda_{l}}}_{\tau }\right\}}^{M_{\tau }}\leq \max_{\tau }\left\{{\bar{\lambda_{l}}}_{\tau }\right\} \end{array}$$
and
  (82) $$\begin{array}{c} \min_{\tau }\left\{{\bar{\nu_{l}}}_{\tau }\right\}\leq \overset{\overset{ˇ}{n}}{\underset{\tau =1}{⊕}}{\left\{{\bar{\nu_{l}}}_{\tau }\right\}}^{M_{\tau }}\leq \max_{\tau }\left\{{\bar{\nu_{l}}}_{\tau }\right\}\text{.} \end{array}$$
Next, for each $\tau =1,2,3,\ldots ,\overset{ˇ}{n}$, we have
  (83) $$\begin{array}{c} \min_{\tau }\left\{\underline{\nu_{l_{\tau }}}\right\}\leq \left\{\underline{\nu_{l_{\tau }}}\right\}\leq \max_{\tau }\left\{\underline{\nu_{l_{\tau }}}\right\}\text{,}\\ \Leftrightarrow \overset{\overset{ˇ}{n}}{\underset{\tau =1}{⊕}}{\left(\min_{\tau }\left\{\underline{\nu_{l_{\tau }}}\right\}\right)}^{M_{\tau }}\leq \overset{\overset{ˇ}{n}}{\underset{\tau =1}{⊕}}{\left(\underline{\nu_{l_{\tau }}}\right)}^{M_{\tau }}\text{,}\\ \leq \overset{\overset{ˇ}{n}}{\underset{\tau =1}{⊕}}{\left(\max_{\tau }\left\{\underline{\nu_{l_{\tau }}}\right\}\right)}^{M_{\tau }}\text{.} \end{array}$$
This implies that
  (84) $$\begin{array}{c} \min_{\tau }\left\{\underline{\nu_{l_{\tau }}}\right\}\leq \overset{\overset{ˇ}{n}}{\underset{\tau =1}{⊕}}{\left\{\underline{\nu_{l_{\tau }}}\right\}}^{M_{\tau }}\leq \max_{\tau }\left\{\underline{\nu_{l_{\tau }}}\right\}\text{.}\\ \min_{\tau }\left\{\underline{\lambda_{l_{\tau }}}\right\}\leq \overset{\overset{ˇ}{n}}{\underset{\tau =1}{⊕}}{\left\{\underline{\lambda_{l_{\tau }}}\right\}}^{M_{\tau }}\leq \max_{\tau }\left\{\underline{\lambda_{l_{\tau }}}\right\}\text{.}\\ \min_{\tau }\left\{\underline{\mu_{l_{\tau }}}\right\}\leq \overset{\overset{ˇ}{n}}{\underset{\tau =1}{⊕}}{\left\{\underline{\mu_{l_{\tau }}}\right\}}^{M_{\tau }}\leq \max_{\tau }\left\{\underline{\mu_{l_{\tau }}}\right\}\text{.} \end{array}$$
Likewise, we can present that
  (85) $$\begin{array}{c} \min_{\tau }\left\{\bar{\mu_{l_{\tau }}}\right\}\leq \overset{\overset{ˇ}{n}}{\underset{\tau =1}{⊕}}{\left\{\bar{\mu_{l_{\tau }}}\right\}}^{M_{\tau }}\leq \max_{\tau }\left\{\bar{\mu_{l_{\tau }}}\right\}\text{,}\\ \min_{\tau }\left\{{\bar{\lambda_{l}}}_{\tau }\right\}\leq \overset{\overset{ˇ}{n}}{\underset{\tau =1}{⊕}}{\left\{{\bar{\lambda_{l}}}_{\tau }\right\}}^{M_{\tau }}\leq \max_{\tau }\left\{{\bar{\lambda_{l}}}_{\tau }\right\}\text{,} \end{array}$$
and
  (86) $$\begin{array}{c} \min_{\tau }\left\{{\bar{\nu_{l}}}_{\tau }\right\}\leq \overset{\overset{ˇ}{n}}{\underset{\tau =1}{⊕}}{\left\{{\bar{\nu_{l}}}_{\tau }\right\}}^{M_{\tau }}\leq \max_{\tau }\left\{{\bar{\nu_{l}}}_{\tau }\right\}\text{.} \end{array}$$
So from (1)–(7) and (87), we have
  (87) $$\begin{array}{c} {\left(\underline{\emptyset }\left(k\right)\right)}^{-}=\left[\begin{array}{c} \left(\min_{\tau }\left\{\underline{\mu_{l_{\tau }}}\right\},\max_{\tau }\left\{\underline{\lambda_{l_{\tau }}}\right\},\max_{\tau }\left\{\underline{\nu_{l_{\tau }}}\right\}\right)\text{,}\\ \left(\min_{\tau }\left\{\bar{\mu_{l_{\tau }}}\right\},\max_{\tau }\left\{{\bar{\lambda_{l}}}_{\tau }\right\},\max_{\tau }\left\{{\bar{\nu_{l}}}_{\tau }\right\}\right) \end{array}\right]\text{.} \end{array}$$
(3) Monotonicity: Since
  (88) $$\begin{array}{c} I\left(k\right)=\left(\underline{I}\left(k_{\tau }\right),\bar{I}\left(k_{\tau }\right)\right)=\left(\left(\underline{a_{l_{\tau }}},\underline{b_{l_{\tau }}},\underline{c_{l_{\tau }}}\right),\left(\bar{a_{l_{\tau }}},\bar{b_{l_{\tau }}},\bar{c_{l_{\tau }}}\right)\right)\text{.} \end{array}$$
and $\emptyset \left({𝕜}_{\tau }\right)=\left(\underline{\emptyset }\left({𝕜}_{\tau }\right),\bar{\emptyset }\left({𝕜}_{\tau }\right)\right)$ to show that $\underline{ℑ}\left({𝕜}_{\tau }\right)\leq \underline{\emptyset }\left({𝕜}_{\tau }\right)$ and $\bar{ℑ}\left({𝕜}_{\tau }\right)\leq \bar{\emptyset }\left({𝕜}_{\tau }\right)$ (for $\tau =1,2,3,\ldots ,\overset{ˇ}{n}$), so
  (89) $$\begin{array}{c} \underline{a_{l_{\tau }}}\leq \underline{\mu_{l_{\tau }}}\Rightarrow 1-\underline{a_{l_{\tau }}}\leq 1-\underline{\mu_{l_{\tau }}}\text{,}\\ \Rightarrow \overset{\overset{ˇ}{n}}{\underset{\tau =1}{⊕}}{\left(1-\underline{\mu_{l_{\tau }}}\right)}^{M_{\tau }}\leq \overset{\overset{ˇ}{n}}{\underset{\tau =1}{⊕}}{\left(1-\underline{a_{l_{\tau }}}\right)}^{M_{\tau }}\text{,}\\ \Rightarrow \left(1-\overset{\overset{ˇ}{n}}{\underset{\tau =1}{⊕}}{\left(1-\underline{a_{l_{\tau }}}\right)}^{M_{\tau }}\right)\leq \left(1-\overset{\overset{ˇ}{n}}{\underset{\tau =1}{⊕}}{\left(1-\underline{\mu_{l_{\tau }}}\right)}^{M_{\tau }}\right)\text{.} \end{array}$$
Next,
  (90) $$\begin{array}{c} \underline{b_{l_{\tau }}}\geq \underline{\lambda_{l_{\tau }}}\Rightarrow \overset{\overset{ˇ}{n}}{\underset{\tau =1}{⊕}}{\underline{b_{l_{\tau }}}}^{M_{\tau }}\geq \overset{\overset{ˇ}{n}}{\underset{\tau =1}{⊕}}{\underline{\lambda_{l_{\tau }}}}^{M_{\tau }}\text{.} \end{array}$$
Next,
  (91) $$\begin{array}{c} \underline{c_{l_{\tau }}}\geq \underline{\nu_{l_{\tau }}}\Rightarrow \overset{\overset{ˇ}{n}}{\underset{\tau =1}{⊕}}{\underline{c_{l_{\tau }}}}^{M_{\tau }}\geq \overset{\overset{ˇ}{n}}{\underset{\tau =1}{⊕}}{\underline{\nu_{l_{\tau }}}}^{M_{\tau }}\text{.} \end{array}$$
Likewise, we can show that
  (92) $$\begin{array}{c} \left(1-\overset{\overset{ˇ}{n}}{\underset{\tau =1}{⊕}}{\left(1-\bar{a_{l_{\tau }}}\right)}^{M_{\tau }}\right)\leq \left(1-\overset{\overset{ˇ}{n}}{\underset{\tau =1}{⊕}}{\left(1-\bar{\mu_{l_{\tau }}}\right)}^{M_{\tau }}\right)\text{,}\\ \overset{\overset{ˇ}{n}}{\underset{\tau =1}{⊕}}{\left(\bar{b_{l_{\tau }}}\right)}^{M_{\tau }}/\geq \overset{\overset{ˇ}{n}}{\underset{\tau =1}{⊕}}{\left({\bar{\lambda_{l}}}_{\tau }\right)}^{M_{\tau }}\text{,}\\ \overset{\overset{ˇ}{n}}{\underset{\tau =1}{⊕}}{\left(\bar{c_{l_{\tau }}}\right)}^{M_{\tau }}\geq \overset{\overset{ˇ}{n}}{\underset{\tau =1}{⊕}}{\left({\bar{\nu_{l}}}_{\tau }\right)}^{M_{\tau }}\text{.} \end{array}$$
Hence, from (9), (11)–(14), we get $\underline{ℑ}\left({𝕜}_{\tau }\right)\leq \underline{\emptyset }\left({𝕜}_{\tau }\right)$ and $\bar{ℑ}\left({𝕜}_{\tau }\right)\leq \bar{\emptyset }\left({𝕜}_{\tau }\right)$.
Therefore,
  (93) $$\begin{array}{c} SV-NHFRWA\left(I\left(k_{1}\right),I\left(k_{2}\right),\ldots ,I\left(k_{\overset{ˇ}{n}}\right)\right)\leq SV-NHFRWA\left(\emptyset \left(k_{1}\right),\emptyset \left(k_{2}\right),\ldots \emptyset \left(k_{\overset{ˇ}{n}}\right)\right)\text{.} \end{array}$$
(4) Shift invariance: As
  (94) $$\begin{array}{c} I\left(k\right)=\left(\underline{I}\left(k\right),\bar{I}\left(k\right)\right)=\left(\left(\underline{a_{l\left(\Upsilon \right)}},\underline{b_{l\left(\Upsilon \right)}},\left.\underline{c_{l\left(\Upsilon \right)}}\right),\left(\bar{a_{l\left(\Upsilon \right)}}\right.,\left.\bar{b_{l\left(\Upsilon \right)}}\right),\bar{c_{l\left(\Upsilon \right)}}\right)\right) \end{array}$$
is a SV-NHFRN and
  (95) $$\begin{array}{c} \emptyset \left(k_{\tau }\right)=\left(\underline{\emptyset }\left(k_{\tau }\right),\bar{\emptyset }\left(k_{\tau }\right)\right)=\left[\left(\underline{\Delta },\underline{\Lambda },\underline{\nabla }\right),\left(\bar{\Delta_{\tau }},{\bar{\nabla }}_{\tau },{\bar{\Lambda }}_{\tau }\right)\right] \end{array}$$
is the set of values of SV-NHFRNs, so
  (96) $$\begin{array}{c} \emptyset \left(k_{1}\right)⊕I\left(k\right)=\left[\underline{\emptyset }\left(k_{1}\right)⊕\underline{I}\left(k\right),\bar{\emptyset }\left(k_{\tau }\right)⊕\bar{I}\left(k\right)\right]\text{.} \end{array}$$

As

(97) $$\begin{array}{c} \left(\begin{array}{c} \left(1-\left(1-\underline{\mu_{l_{\tau }}}\right)\left(1-\underline{a_{l\left(\Upsilon \right)}}\right),\left(\underline{\lambda_{l_{\tau }}}\underline{b_{l\left(\Upsilon \right)}}\right),\left(\underline{\nu_{l_{\tau }}}\underline{c_{l\left(\Upsilon \right)}}\right)\right)\text{,}\\ \left(1-\left(1-\bar{\mu_{l_{\tau }}}\right)\left(1-\bar{a_{l\left(\Upsilon \right)}}\right),\left({\bar{\lambda_{l}}}_{\tau }\times \bar{b_{l\left(\Upsilon \right)}}\right),\left({\bar{\nu_{l}}}_{\tau }\times \bar{c_{l\left(\Upsilon \right)}}\right)\right) \end{array}\right)\text{.} \end{array}$$

Thus, SV-NHFRN

(98) $$\begin{array}{c} I\left(k\right)=\left(\underline{I}\left(k\right),\bar{I}\left(k\right)\right)=\left(\left(\underline{a_{l\left(\Upsilon \right)}},\underline{b_{l\left(\Upsilon \right)}},\underline{c_{l\left(\Upsilon \right)}}\right),\left(\bar{a_{l\left(\Upsilon \right)}},\bar{b_{l\left(\Upsilon \right)}},\bar{c_{l\left(\Upsilon \right)}}\right)\right)\text{.} \end{array}$$

It follows that

(99) $$\begin{array}{c} SV-NHFRWA\left(\emptyset \left(k_{1}\right)⊕I\left(k\right),\emptyset \left(k_{2}\right)⊕I\left(k\right),\ldots ,\emptyset \left(k_{\overset{ˇ}{n}}\right)⊕I\left(k\right)\right)\text{,}\\ =\left(\left[\begin{array}{c} \underset{\underline{\mu_{l_{\tau }}}\in \Delta_{l_{\underline{\emptyset }\left(k\right)}}}{\cup }\left(\begin{array}{c} 1-\overset{\overset{ˇ}{n}}{\underset{\tau =1}{⊕}}{\left(1-\underline{\mu_{l_{\tau }}}\right)}^{M_{\tau }}\\ {\left(1-\underline{a_{l\left(\Upsilon \right)}}\right)}^{M_{\tau }} \end{array}\right)\text{,}\\ \;\underset{\underline{\lambda_{l_{\tau }}}\in \Lambda_{l_{\underline{\emptyset }\left(k\right)}}}{\cup }\left(\overset{\overset{ˇ}{n}}{\underset{\tau =1}{⊕}}{\left(\underline{\lambda_{l_{\tau }}}\right)}^{M_{\tau }}{\underline{b_{l\left(\Upsilon \right)}}}^{M_{\tau }}\right)\text{,}\\ \;\underset{\underline{\nu_{l_{\tau }}}\in \nabla_{l_{\underline{\emptyset }\left(k\right)}}}{\cup }\left(\overset{\overset{ˇ}{n}}{\underset{\tau =1}{⊕}}{\left(\underline{\nu_{l_{\tau }}}\right)}^{M_{\tau }}{\underline{c_{l\left(\Upsilon \right)}}}^{M_{\tau }}\right) \end{array}\right]\right)\text{,}\\ =\left[\begin{array}{c} \left\{\overset{\overset{ˇ}{n}}{\underset{\tau =1}{⊕}}M_{\tau }\underline{\emptyset }\left(k_{\tau }\right)⊕I\left(k\right),\overset{\overset{ˇ}{n}}{\underset{\tau =1}{⊕}}M_{\tau }\left(\bar{\emptyset }\left(k_{\tau }\right)⊕I\left(k\right)\right)\right\}\text{,}\\ \left\{\begin{array}{c} \underset{\bar{\mu_{l_{\tau }}}\in \Delta_{l_{\bar{\emptyset }\left(k\right)}}}{\cup }\left(\begin{array}{c} \left(1-\overset{\overset{ˇ}{n}}{\underset{\tau =1}{⊕}}{\left(1-\bar{\mu_{l_{\tau }}}\right)}^{M_{\tau }}\right)\\ {\left(1-{\bar{a}}_{l\left(\Upsilon \right)}\right)}^{M_{\tau }} \end{array}\right),\\ \;\underset{\bar{\lambda_{l_{\tau }}}\in \Lambda_{l_{\bar{\emptyset }\left(k\right)}}}{\cup }\left(\bar{b_{l\left(\Upsilon \right)}}\overset{\overset{ˇ}{n}}{\underset{\tau =1}{⊕}}{\left({\bar{\lambda_{l}}}_{\tau }\right)}^{M_{\tau }}\right),\\ \;\underset{\bar{\nu_{l_{\tau }}}\in \nabla_{l_{\bar{\emptyset }\left(k\right)}}}{\cup }\left(\bar{c_{l\left(\Upsilon \right)}}\overset{\overset{ˇ}{n}}{\underset{\tau =1}{⊕}}{\left({\bar{\nu_{l}}}_{\tau }\right)}^{M_{\tau }}\right) \end{array}\right\} \end{array}\right]\text{,}\\ =\left[\begin{array}{c} \left\{\begin{array}{c} \left(\begin{array}{c} \underset{\underline{\mu_{l_{\tau }}}\in \Delta_{l_{\underline{\emptyset }\left(k\right)}}}{\cup }\left(1-\overset{\overset{ˇ}{n}}{\underset{\tau =1}{⊕}}{\left(1-\underline{\mu_{l_{\tau }}}\right)}^{M_{\tau }}\right),\\ \underset{\underline{\lambda_{l_{\tau }}}\in \Lambda_{l_{\underline{\emptyset }\left(k\right)}}}{\cup }\overset{\overset{ˇ}{n}}{\underset{\tau =1}{⊕}}{\left(\underline{\hbar_{l_{\tau }}}\right)}^{M_{\tau }},\\ \underset{\underline{\nu_{l_{\tau }}}\in \nabla_{l_{\underline{\emptyset }\left(k\right)}}}{\cup }\overset{\overset{ˇ}{n}}{\underset{\tau =1}{⊕}}{\left(\underline{\nu_{l_{\tau }}}\right)}^{M_{\tau }} \end{array}\right)\\ ⊕\left(\underline{a_{l\left(\Upsilon \right)}},\underline{b_{l\left(\Upsilon \right)}},\underline{c_{l\left(\Upsilon \right)}}\right) \end{array}\right\}\text{,}\\ \left\{\begin{array}{c} \left(\begin{array}{c} \underset{\bar{\mu_{l_{\tau }}}\in \Delta_{l_{\bar{\emptyset }\left(k\right)}}}{\cup }\left(1-\overset{\overset{ˇ}{n}}{\underset{\tau =1}{⊕}}{\left(1-\bar{\mu_{l_{\tau }}}\right)}^{M_{\tau }}\right),\\ \;\underset{\bar{\lambda_{l_{\tau }}}\in \Lambda_{l_{\bar{\emptyset }\left(k\right)}}}{\cup }\overset{\overset{ˇ}{n}}{\underset{\tau =1}{⊕}}{\left({\bar{\lambda_{l}}}_{\tau }\right)}^{M_{\tau }},\\ \;\underset{\bar{\nu_{l_{\tau }}}\in \nabla_{l_{\bar{\emptyset }\left(k\right)}}}{\cup }\overset{\overset{ˇ}{n}}{\underset{\tau =1}{⊕}}{\left({\bar{\nu_{l}}}_{\tau }\right)}^{M_{\tau }} \end{array}\right)\\ ⊕\left(\bar{a_{l\left(\Upsilon \right)}},\bar{b_{l\left(\Upsilon \right)}},\bar{c_{l\left(\Upsilon \right)}}\right) \end{array}\right\} \end{array}\right]\text{,}\\ =\left[\begin{array}{c} \left(\begin{array}{c} \underset{\underline{\mu_{l_{\tau }}}\in \Delta_{l_{\underline{\emptyset }\left(k\right)}}}{\cup }\left(1-\overset{\overset{ˇ}{n}}{\underset{\tau =1}{⊕}}{\left(1-\underline{\mu_{l_{\tau }}}\right)}^{M_{\tau }}\right),\\ \underset{\underline{\lambda_{l_{\tau }}}\in \Lambda_{l_{\underline{\emptyset }\left(k\right)}}}{\cup }\overset{\overset{ˇ}{n}}{\underset{\tau =1}{⊕}}{\left(\underline{\lambda_{l_{\tau }}}\right)}^{M_{\tau }},\\ \underset{\underline{\nu_{l_{\tau }}}\in \nabla_{l_{\underline{\emptyset }\left(k\right)}}}{\cup }\overset{\overset{ˇ}{n}}{\underset{\tau =1}{⊕}}{\left(\underline{\nu_{l_{\tau }}}\right)}^{M_{\tau }} \end{array}\right)\text{,}\\ \left(\begin{array}{c} \underset{\bar{\mu_{l_{\tau }}}\in \Delta_{l_{\bar{\emptyset }\left(k\right)}}}{\cup }\left(1-\overset{\overset{ˇ}{n}}{\underset{\tau =1}{⊕}}{\left(1-\bar{\mu_{l_{\tau }}}\right)}^{M_{\tau }}\right),\\ \;\underset{\bar{\lambda_{l_{\tau }}}\in \Lambda_{l_{\bar{\emptyset }\left(k\right)}}}{\cup }\overset{\overset{ˇ}{n}}{\underset{\tau =1}{⊕}}{\left({\bar{\lambda_{l}}}_{\tau }\right)}^{M_{\tau }}\\ \;\underset{\bar{\nu_{l_{\tau }}}\in \nabla_{l_{\bar{\emptyset }\left(k\right)}}}{\cup }\overset{\overset{ˇ}{n}}{\underset{\tau =1}{⊕}}{\left({\bar{\nu_{l}}}_{\tau }\right)}^{M_{\tau }} \end{array}\right) \end{array}\right]\text{,}\\ ⊕\left[\left(\underline{a_{l\left(\Upsilon \right)}},\underline{b_{l\left(\Upsilon \right)}},\underline{c_{l\left(\Upsilon \right)}}\right),\left(\bar{a_{l\left(\Upsilon \right)}},\bar{b_{l\left(\Upsilon \right)}},\bar{c_{l\left(\Upsilon \right)}}\right)\right]\text{,}\\ =SV-NHFRWA\left(\emptyset \left(k_{1}\right),\emptyset \left(k_{2}\right),\ldots ,\emptyset \left(k_{\overset{ˇ}{n}}\right)\right)⊕I\left(k\right)\text{.} \end{array}$$

**Proof**

(1) Homogeneity: Let real number Δ > 0 and $\emptyset \left({𝕜}_{\tau }\right)=\left(\underline{\emptyset }\left({𝕜}_{\tau }\right),\bar{\emptyset }\left({𝕜}_{\tau }\right)\right)$ be a SV-NHFRNs. Consider
  (100) $$\begin{array}{c} \Delta \emptyset \left(k_{\tau }\right)=\left(\Delta \underline{\emptyset }\left(k_{\tau }\right),\Delta \bar{\emptyset }\left(k_{\tau }\right)\right)\text{,}\\ =\left[\begin{array}{c} \left\{\begin{array}{c} \underset{\underline{\mu_{l_{\tau }}}\in \Delta_{l_{\underline{\emptyset }\left(k\right)}}}{\cup }\left(\left(1-{\left(1-\underline{\mu_{l_{\tau }}}\right)}^{\Delta }\right)\right),\\ \underset{\underline{\lambda_{l_{\tau }}}\in \Lambda_{l_{\underline{\emptyset }\left(k\right)}}}{\cup }\left({\underline{\lambda_{l_{\tau }}}}^{\Delta }\right),\\ \underset{\underline{\nu_{l_{\tau }}}\in \nabla_{l_{\underline{\emptyset }\left(k\right)}}}{\cup }\left({\underline{\nu_{l_{\tau }}}}^{\Delta }\right) \end{array}\right\}\text{,}\\ \left\{\begin{array}{c} \underset{\bar{\mu_{l_{\tau }}}\in \Delta_{l_{\bar{\emptyset }\left(k\right)}}}{\cup }\left(1-{\left(1-\bar{\mu_{l_{\tau }}}\right)}^{\Delta }\right),\\ \underset{\bar{\lambda_{l_{\tau }}}\in \Lambda_{l_{\underline{\emptyset }\left(k\right)}}}{\cup }\left({\bar{\lambda_{l_{\tau }}}}_{\tau }\right),\\ \underset{\bar{\nu_{l_{\tau }}}\in \nabla_{l_{\underline{\emptyset }\left(k\right)}}}{\cup }\left({\bar{\nu_{l_{\tau }}}}_{\tau }\right) \end{array}\right\}\text{.} \end{array}\right]\text{.} \end{array}$$
Now,
  (101) $$\begin{array}{c} SV-NHFRWA\left(\Delta \emptyset \left(k_{1}\right),\Delta \emptyset \left(k_{2}\right),\ldots ,\Delta \emptyset \left(k_{\overset{ˇ}{n}}\right)\right)\text{,}\\ =\left[\begin{array}{c} \left(\begin{array}{c} \underset{\underline{\mu_{l_{\tau }}}\in \Delta_{l_{\underline{\emptyset }\left(k\right)}}}{\cup }\left(1-\overset{\overset{ˇ}{n}}{\underset{\tau =1}{⊕}}{\left(1-\underline{\mu_{l_{\tau }}}\right)}^{\Delta }\right),\\ \underset{\underline{\lambda_{l_{\tau }}}\in \Lambda_{l_{\underline{\emptyset }\left(k\right)}}}{\cup }\left(\overset{\overset{ˇ}{n}}{\underset{\tau =1}{⊕}}{\left(\underline{\lambda_{l_{\tau }}}\right)}^{\Delta }\right),\\ \underset{\underline{\nu_{l_{\tau }}}\in \nabla_{l_{\underline{\emptyset }\left(k\right)}}}{\cup }\left(\overset{\overset{ˇ}{n}}{\underset{\tau =1}{⊕}}{\left(\underline{\nu_{l_{\tau }}}\right)}^{\Delta }\right) \end{array}\right)\text{,}\\ \left(\begin{array}{c} \underset{\bar{\mu_{l_{\tau }}}\in \Delta_{l_{\bar{\emptyset }\left(k\right)}}}{\cup }\left(1-\overset{\overset{ˇ}{n}}{\underset{\tau =1}{⊕}}{\left(1-\bar{\mu_{l_{\tau }}}\right)}^{\Delta }\right),\\ \underset{\bar{\lambda_{l_{\tau }}}\in \Lambda_{l_{\underline{\emptyset }\left(k\right)}}}{\cup }\left(\overset{\overset{ˇ}{n}}{\underset{\tau =1}{⊕}}{\left({\bar{\lambda_{l}}}_{\tau }\right)}^{\Delta }\right),\\ \underset{\bar{\nu_{l_{\tau }}}\in \nabla_{l_{\underline{\emptyset }\left(k\right)}}}{\cup }\left(\overset{\overset{ˇ}{n}}{\underset{\tau =1}{⊕}}{\left({\bar{\nu_{l}}}_{\tau }\right)}^{\Delta }\right) \end{array}\right) \end{array}\right]\text{,}\\ =\Delta SV-NHFRWA\left(\emptyset \left(k_{1}\right),\emptyset \left(k_{2}\right),\ldots ,\emptyset \left(k_{\overset{ˇ}{n}}\right)\right)\text{.} \end{array}$$
(2) Commutativity: Suppose
  (102) $$\begin{array}{c} SV-NHFRWA\left(\emptyset \left(k_{1}\right),\emptyset \left(k_{2}\right),\ldots ,\emptyset \left(k_{\overset{ˇ}{n}}\right)\right)\text{,}\\ =\left[\overset{\overset{ˇ}{n}}{\underset{\tau =1}{⊕}}\Delta_{\tau }\underline{\emptyset }\left(k_{\tau }\right),\overset{\overset{ˇ}{n}}{\underset{\tau =1}{⊕}}\Delta_{\tau }\bar{\emptyset }\left(k_{\tau }\right)\right]\text{,}\\ =\left[\begin{array}{c} \left(\begin{array}{c} \underset{\underline{\mu_{l_{\tau }}}\in \Delta_{l_{\underline{\emptyset }\left(k\right)}}}{\cup }\left(1-\overset{\overset{ˇ}{n}}{\underset{\tau =1}{⊕}}{\left(1-\underline{\mu_{l_{\tau }}}\right)}^{\Delta_{\tau }}\right),\\ \underset{\underline{\lambda_{l_{\tau }}}\in \Lambda_{l_{\underline{\emptyset }\left(k\right)}}}{\cup }\left(\overset{\overset{ˇ}{n}}{\underset{\tau =1}{⊕}}{\left(\underline{\lambda_{l_{\tau }}}\right)}^{\Delta_{\tau }}\right),\\ \underset{\underline{\nu_{l_{\tau }}}\in \nabla_{l_{\underline{\emptyset }\left(k\right)}}}{\cup }\left(\overset{\overset{ˇ}{n}}{\underset{\tau =1}{⊕}}{\left(\underline{\nu_{l_{\tau }}}\right)}^{\Delta_{\tau }}\right). \end{array}\right)\text{,}\\ \left(\begin{array}{c} \underset{\bar{\mu_{l_{\tau }}}\in \Delta_{l_{\bar{\emptyset }\left(k\right)}}}{\cup }\left(1-\overset{\overset{ˇ}{n}}{\underset{\tau =1}{⊕}}{\left(1-\bar{\mu_{l_{\tau }}}\right)}^{\Delta_{\tau }}\right),\\ \underset{\bar{\lambda_{l_{\tau }}}\in \Lambda_{l_{\underline{\emptyset }\left(k\right)}}}{\cup }\left(\overset{\overset{ˇ}{n}}{\underset{\tau =1}{⊕}}{\left({\bar{\lambda_{l}}}_{\tau }\right)}^{\Delta_{\tau }}\right),\\ \underset{\bar{\nu_{l_{\tau }}}\in \nabla_{l_{\underline{\emptyset }\left(k\right)}}}{\cup }\left(\overset{\overset{ˇ}{n}}{\underset{\tau =1}{⊕}}{\left({\bar{\nu_{l}}}_{\tau }\right)}^{\Delta_{\tau }}\right). \end{array}\right) \end{array}\right]\text{.} \end{array}$$

Let $\left(\emptyset^{\prime }\left({𝕜}_{1}\right),\emptyset^{\prime }\left({𝕜}_{2}\right),\ldots ,\emptyset^{\prime }\left({𝕜}_{\overset{ˇ}{n}}\right)\right)$ be a permutation of $\left(\emptyset \left({𝕜}_{1}\right),\emptyset \left({𝕜}_{2}\right),\ldots ,\emptyset \left({𝕜}_{\overset{ˇ}{n}}\right)\right)$. Then, we have $\emptyset \left({𝕜}_{\tau }\right)=\emptyset^{\prime }\left({𝕜}_{\tau }\right)\left(\tau =1,2,3,\ldots ,\overset{ˇ}{n}\right)$

(103) $$\begin{array}{c} =\left[\begin{array}{c} \left(\begin{array}{c} \underset{\underline{\mu_{l_{\tau }}}\in \Delta_{l_{\underline{\emptyset }\left(k\right)}}}{\cup }\left(1-\overset{\overset{ˇ}{n}}{\underset{\tau =1}{⊕}}{\left(1-\underline{\mu_{l_{\tau }}}\right)}^{\Delta_{\tau }}\right),\\ \underset{\underline{\lambda_{l_{\tau }}}\in \Lambda_{l_{\underline{\emptyset }\left(k\right)}}}{\cup }\left(\overset{\overset{ˇ}{n}}{\underset{\tau =1}{⊕}}{\left(\underline{\lambda_{l_{\tau }}}\right)}^{\Delta_{\tau }}\right),\\ \underset{\underline{\nu_{l_{\tau }}}\in \nabla_{l_{\underline{\emptyset }\left(k\right)}}}{\cup }\left(\overset{\overset{ˇ}{n}}{\underset{\tau =1}{⊕}}{\left(\underline{\nu_{l_{\tau }}}\right)}^{\Delta_{\tau }}\right), \end{array}\right)\text{,}\\ \left(\begin{array}{c} \underset{\bar{\mu_{l_{\tau }}}\in \Delta_{l_{\bar{\emptyset }\left(k\right)}}}{\cup }\left(1-\overset{\overset{ˇ}{n}}{\underset{\tau =1}{⊕}}{\left(1-\bar{\mu_{l_{\tau }}}\right)}^{\Delta_{\tau }}\right),\\ \underset{\bar{\lambda_{l_{\tau }}}\in \Lambda_{l_{\underline{\emptyset }\left(k\right)}}}{\cup }\left(\overset{\overset{ˇ}{n}}{\underset{\tau =1}{⊕}}{\left(\bar{\lambda_{l_{\tau }}}\right)}^{\Delta_{\tau }}\right),\\ \underset{\bar{\nu_{l_{\tau }}}\in \nabla_{l_{\underline{\emptyset }\left(k\right)}}}{\cup }\left(\overset{\overset{ˇ}{n}}{\underset{\tau =1}{⊕}}{\left(\bar{\nu_{l_{\tau }}}\right)}^{\Delta_{\tau }}\right) \end{array}\right) \end{array}\right]\text{,}\\ =\left[\overset{\overset{ˇ}{n}}{\underset{\tau =1}{⊕}}\Delta_{\tau }{\underline{\emptyset }}^{\prime }\left(k_{\tau }\right),\overset{\overset{ˇ}{n}}{\underset{\tau =1}{⊕}}\Delta_{\tau }\bar{\emptyset^{\prime }}\left(k_{\tau }\right)\right]\text{,}\\ =SV-NHFRWA\left(\emptyset^{\prime }\left(k_{1}\right),\emptyset^{\prime }\left(k_{2}\right),\ldots ,\emptyset^{\prime }\left(k_{\overset{ˇ}{n}}\right)\right)\text{.} \end{array}$$

**Definition 18 .**

The set of values $\emptyset \left({𝕜}_{\tau }\right)=\left(\underline{\emptyset }\left({𝕜}_{\tau }\right),\bar{\emptyset }\left({𝕜}_{\tau }\right)\right)\left(\tau =1,2,3,4,\ldots ,\overset{ˇ}{n}\right)$ of SV-NHFRNs with weight vector $M={\left(M_{1},M_{2},\ldots M_{\overset{ˇ}{n}}\right)}^{\delta }$ is

such that ${⊕}_{\tau =1}^{\overset{ˇ}{n}}M_{\tau }=1$ and 0 ≤ *M*_*τ*_ ≤ 1. The SV-NHFROWA operator is determined as

(104) $$\begin{array}{c} SV-NHFROWA\left(\emptyset \left(k_{1}\right),\emptyset \left(k_{2}\right),\ldots ,\emptyset \left(k_{\overset{ˇ}{n}}\right)\right)\\ =\left(\overset{\overset{ˇ}{n}}{\underset{\tau =1}{⊕}}M_{\tau }\underline{\emptyset }\left(k_{\tau }\right),\overset{\overset{ˇ}{n}}{\underset{\tau =1}{⊕}}M_{\tau }\bar{\emptyset_{\nabla_{\tau }}}\left(k_{\tau }\right)\right)\text{.}\\ \left(\begin{array}{c} \left(\begin{array}{c} \underset{\underline{\mu_{l_{\tau }}}\in \Delta_{l_{\underline{\emptyset }\left(k\right)}}}{\cup }\left(1-\overset{k+1}{\underset{\tau =1}{⊗}}{\left(1-\underline{\mu_{l_{\tau }}}\right)}^{M_{\tau }}\right),\\ \underset{\underline{\lambda_{l_{\tau }}}\in \Lambda_{l_{\underline{\emptyset }\left(k\right)}}}{\cup }\left(\overset{k+1}{\underset{\tau =1}{⊗}}{\left(\underline{\lambda_{l_{\tau }}}\right)}^{M_{\tau }}\right),\\ \underset{\underline{\nu_{l_{\tau }}}\in \nabla_{l_{\underline{\emptyset }\left(k\right)}}}{\cup }\left(\overset{k+1}{\underset{\tau =1}{⊗}}{\left(\underline{\nu_{l_{\tau }}}\right)}^{M_{\tau }}\right) \end{array}\right)\\ \left(\begin{array}{c} \underset{\bar{\mu_{l_{\tau }}}\in \Delta_{l_{\bar{\emptyset }\left(k\right)}}}{\cup }\left(1-\overset{k+1}{\underset{\tau =1}{⊗}}{\left(1-\bar{\mu_{l_{\tau }}}\right)}^{M_{\tau }}\right),\\ \underset{\;\bar{\lambda_{l_{\tau }}}\in \Lambda_{l_{\bar{\emptyset }\left(k\right)}}}{\cup }\left(\overset{k+1}{\underset{\tau =1}{⊗}}{\left(\bar{\lambda_{l_{\tau }}}\right)}^{M_{\tau }}\right),\\ \;\underset{\bar{\nu_{l_{\tau }}}\in \nabla_{l_{\bar{\emptyset }\left(k\right)}}}{\cup }\left(\overset{k+1}{\underset{\tau =1}{⊗}}{\left(\bar{\nu_{l_{\tau }}}\right)}^{M_{\tau }}\right) \end{array}\right) \end{array}\right)\text{.} \end{array}$$

**Theorem 3 .**

Let $\emptyset \left({𝕜}_{\tau }\right)=\left(\underline{\emptyset }\left({𝕜}_{\tau }\right),\bar{\emptyset }\left({𝕜}_{\tau }\right)\right)\left(\tau =1,2,3,\ldots \overset{ˇ}{n}\right)$ be the set of values of SV-NHFRNs with $M={\left(M_{1},M_{2},\ldots M_{\overset{ˇ}{n}}\right)}^{\delta }$.

Then, the SV-NHFROWA operator is defined as

(105) $$\begin{array}{c} SV-NHFROWA\left(\emptyset \left(k_{1}\right),\emptyset \left(k_{2}\right),\ldots \emptyset \left(k_{\overset{ˇ}{n}}\right)\right)\text{,}\\ =\left(\overset{\overset{ˇ}{n}}{\underset{\tau =1}{⊕}}M_{\tau }NHFROWA\left(k_{\tau }\right),\overset{\overset{ˇ}{n}}{\underset{\tau =1}{⊕}}M_{\tau }\bar{\emptyset_{\nabla_{\tau }}}\left(k_{\tau }\right)\right)\text{,}\\ =\left[\begin{array}{c} \left(\begin{array}{c} \underset{\underline{\mu_{l_{\nabla_{\tau }}}}\in \Delta_{l_{\underline{\emptyset }\left(k\right)}}}{\cup }\left(1-\overset{\overset{ˇ}{n}}{\underset{\tau =1}{⊕}}{\left(1-\underline{\mu_{l_{\nabla_{\tau }}}}\right)}^{M_{\tau }}\right),\\ \underset{\underline{\lambda_{l_{\nabla_{\tau }}}}\in \Lambda_{l_{\underline{\emptyset }\left(k\right)}}}{\cup }\left(\overset{\overset{ˇ}{n}}{\underset{\tau =1}{⊕}}{\left(\underline{\lambda_{l_{\nabla_{\tau }}}}\right)}^{M_{\tau }}\right),\\ \underset{\underline{\nu_{l_{\nabla_{\tau }}}}\in \nabla_{l_{\underline{\emptyset }\left(k\right)}}}{\cup }\left(\overset{\overset{ˇ}{n}}{\underset{\tau =1}{⊕}}{\left(\underline{\nu_{l_{\nabla_{\tau }}}}\right)}^{M_{\tau }}\right) \end{array}\right)\\ \left(\begin{array}{c} \underset{\bar{\mu_{l_{\nabla_{\tau }}}}\in \Delta_{l_{\bar{\emptyset }\left(k\right)}}}{\cup }\left(1-\overset{\overset{ˇ}{n}}{\underset{\tau =1}{⊕}}{\left(1-\bar{\mu_{l_{\nabla_{\tau }}}}\right)}^{M_{\tau }}\right),\\ \underset{\bar{\lambda_{l_{\nabla_{\tau }}}}\in \Lambda_{l_{\bar{\emptyset }\left(k\right)}}}{\cup }\left(\overset{\overset{ˇ}{n}}{\underset{\tau =1}{⊕}}{\left(\bar{\lambda_{l_{\nabla_{\tau }}}}\right)}^{M_{\tau }}\right),\\ \underset{\bar{\nu_{l_{\nabla_{\tau }}}}\in \nabla_{l_{\bar{\emptyset }\left(k\right)}}}{\cup }\left(\overset{\overset{ˇ}{n}}{\underset{\tau =1}{⊕}}{\left(\bar{\nu_{l_{\nabla_{\tau }}}}\right)}^{M_{\tau }}\right) \end{array}\right) \end{array}\right]\text{.} \end{array}$$

where $\emptyset_{\nabla }\left({𝕜}_{\tau }\right)=\left(\underline{\emptyset }\left({𝕜}_{\tau }\right),\bar{\emptyset_{\nabla_{\tau }}}\left({𝕜}_{\tau }\right)\right)$ depicts the superior value of permutation from the set of values of SV-NHFRNs.

**Proof**

The proof is similar to the proof of Theorem 1.

**Theorem 4 .**

Let

(106) $$\begin{array}{c} \emptyset \left(k_{\tau }\right)=\left(\underline{\emptyset }\left(k_{\tau }\right),\bar{\emptyset }\left(k_{\tau }\right)\right)\left(\tau =1,2,3,4,\ldots ,\overset{ˇ}{n}\right) \end{array}$$

be the set of values of SV-NHFRNs with weight vectors $M={\left(M_{1},M_{2},\ldots M_{\overset{ˇ}{n}}\right)}^{\delta }$ such that ${⊕}_{\tau =1}^{\overset{ˇ}{n}}M_{\tau }=1$ and 0 ≤ *M*_*τ*_ ≤ 1.

Then, the SV-NHFROWA operator satisfies the following properties:

(1) Idempotency: If ∅(*𝕜*_*τ*_) = *ℑ*(*𝕜*) for $\tau =1,2,3,\ldots ,\overset{ˇ}{n}$, where
  (107) $$\begin{array}{c} I\left(k\right)=\left(\underline{I}\left(k\right),\bar{I}\left(k\right)\right)=\left(\left(\underline{a_{l\left(\Upsilon \right)}},\underline{b_{l\left(\Upsilon \right)}},\underline{c_{l\left(\Upsilon \right)}}\right),\left({\bar{a}}_{l\left(\Upsilon \right)},\bar{b_{l\left(\Upsilon \right)}},\bar{c_{l\left(\Upsilon \right)}}\right)\right)\text{.} \end{array}$$
Then,
  (108) $$\begin{array}{c} SV-NHFROWA\left(\emptyset \left(k_{1}\right),\emptyset \left(k_{2}\right),\ldots \emptyset \left(k_{\overset{ˇ}{n}}\right)\right)=I\left(k\right)\text{.} \end{array}$$
(2) Boundedness**:** Let ${\left(\emptyset \left(𝕜\right)\right)}^{-}=\left(\min_{\tau }\underline{\emptyset }\left({𝕜}_{\tau }\right),\max_{\tau }\bar{\emptyset }\left({𝕜}_{\tau }\right)\right)$ and ${\left(\emptyset \left(𝕜\right)\right)}^{+}=\left(\max_{\tau }\underline{\emptyset }\left({𝕜}_{\tau }\right),\min_{\tau }\bar{\emptyset }\left({𝕜}_{\tau }\right)\right)$. Then,
  (109) $$\begin{array}{c} {\left(\emptyset \left(k\right)\right)}^{-}\leq SV-NHFROWA\left(\emptyset \left(k_{1}\right),\emptyset \left(k_{2}\right),\ldots ,\emptyset \left(k_{\overset{ˇ}{n}}\right)\right)\leq {\left(\emptyset \left(k\right)\right)}^{+}\text{.} \end{array}$$
(3) Monotonicity**:** Suppose $ℑ\left(𝕜\right)=\left(\underline{ℑ}\left({𝕜}_{\tau }\right),\bar{ℑ}\left({𝕜}_{\tau }\right)\right)\left(\tau =\tau ,2,\ldots ,\overset{ˇ}{n}\right)$ is another set of values of SV-NHFRNssuch that $\underline{ℑ}\left({𝕜}_{\tau }\right)\leq \underline{\emptyset }\left({𝕜}_{\tau }\right)$ and $\bar{ℑ}\left({𝕜}_{\tau }\right)\leq \bar{\emptyset }\left({𝕜}_{\tau }\right)$. Then,
  (110) $$\begin{array}{c} SV-NHFROWA\left(I\left(k_{1}\right),I\left(k_{2}\right),\ldots ,I\left(k_{\overset{ˇ}{n}}\right)\right)\leq SV-NHFROWA\left(\emptyset \left(k_{1}\right),\emptyset \left(k_{2}\right),\ldots \emptyset \left(k_{\overset{ˇ}{n}}\right)\right)\text{.} \end{array}$$
(4) Shift invariance**:** Consider another SV-NHFRN $ℑ\left(𝕜\right)=\left(\underline{ℑ}\left(𝕜\right),\bar{ℑ}\left(𝕜\right)\right)=\left(\left(\underline{b},\underline{d},\underline{c}\right),\left(\bar{b},\bar{d},\bar{c}\right)\right)$. Then,
  (111) $$\begin{array}{c} SV-NHFROWA\left(\emptyset \left(k_{1}\right)⊕I\left(k\right),\emptyset \left(k_{2}\right)⊕I\left(k\right),\ldots ,\emptyset \left(k_{\overset{ˇ}{n}}\right)⊕I\left(k\right)\right)\text{,}\\ =SV-NHFROWA\left(\emptyset \left(k_{1}\right),\emptyset \left(k_{2}\right),\ldots \emptyset \left(k_{\overset{ˇ}{n}}\right)\right)⊕I\left(k\right)\text{.} \end{array}$$
(5) Homogeneity**:** For any real number Δ > 0,
  (112) $$\begin{array}{c} SV-NHFROWA\left(\Delta \emptyset \left(k_{1}\right),\Delta \emptyset \left(k_{2}\right),\ldots ,\Delta \emptyset \left(k_{\overset{ˇ}{n}}\right)\right)=\Delta \times SV-NHFROWA\left(\emptyset \left(k_{1}\right),\emptyset \left(k_{2}\right),\ldots ,\emptyset \left(k_{\overset{ˇ}{n}}\right)\right)\text{.} \end{array}$$
(6) Commutativity**:** Suppose $\emptyset^{\prime }\left({𝕜}_{\tau }\right)=\left(\underline{\emptyset }\left({𝕜}_{\tau }\right),\bar{\emptyset^{\prime }}\left({𝕜}_{\tau }\right)\right)$ and $\emptyset \left({𝕜}_{\tau }\right)=\left(\underline{\emptyset }\left({𝕜}_{\tau }\right),\bar{\emptyset }\left({𝕜}_{\tau }\right)\right)$ and $\left(\tau =1,2,3,\ldots ,\overset{ˇ}{n}\right)$ be any SV-NHFRNs.

Then

(113) $$\begin{array}{c} SV-NHFROWA\left(\emptyset \left(k_{1}\right),\emptyset \left(k_{2}\right),\ldots ,\emptyset \left(k_{\overset{ˇ}{n}}\right)\right)=SV-NHFROWA\left(\emptyset^{\prime }\left(k_{1}\right),\emptyset^{\prime }\left(k_{2}\right),\ldots ,\emptyset^{\prime }\left(k_{\overset{ˇ}{n}}\right)\right)\text{.} \end{array}$$

**Proof**

The argument is identical to the proof of Theorem 20.

## 5. SV-Neutrosophic Hesitant Fuzzy Rough Geometric Aggregation Operator

In this section, we discuss the SV-NHFR geometric aggregation operator by employing the idea of rough sets into SV-NHF geometric operators. The important characteristics of the developed operators are illustrated.

**Definition 19 .**

Let $\emptyset \left({𝕜}_{\tau }\right)=\left(\underline{\emptyset }\left({𝕜}_{\tau }\right),\bar{\emptyset }\left({𝕜}_{\tau }\right)\right)\left(\tau =1,2,3,4,\ldots ,\overset{ˇ}{n}\right)$ be the set of values of SV-NHFRNs with $M={\left(M_{1},M_{2},\ldots M_{\overset{ˇ}{n}}\right)}^{\delta }$ such that ${⊕}_{\tau =1}^{\overset{ˇ}{n}}M_{\tau }=1$ and 0 ≤ *M*_*τ*_ ≤ 1. Then, the SV-NHFRWGA operator is determined as

(114) $$\begin{array}{c} SV-NHFRWG\left(\emptyset \left(k_{1}\right),\emptyset \left(k_{2}\right),\ldots ,\emptyset \left(k_{\overset{ˇ}{n}}\right)\right)=\left({⊕}_{\tau =1}^{\overset{ˇ}{n}}{\left(\underline{\emptyset }\left(k_{\tau }\right)\right)}^{M_{\tau }},{⊕}_{\tau =1}^{\overset{ˇ}{n}}{\left(\bar{\emptyset }\left(k_{\tau }\right)\right)}^{M_{\tau }}\right)\text{.} \end{array}$$

The aggregated conclusion for the SV-NHFRWGA operator is given in the following theorem, which is based on the above mentioned formulation.

**Theorem 5 .**

Let $\emptyset \left({𝕜}_{\tau }\right)=\left(\underline{\emptyset }\left({𝕜}_{\tau }\right),\bar{\emptyset }\left({𝕜}_{\tau }\right)\right)\left(\tau =1,2,3,\ldots \overset{ˇ}{n}\right)$ be the set of values of SV-NHFRNs with $M={\left(M_{1},M_{2},\ldots M_{\overset{ˇ}{n}}\right)}^{\delta }$ such that ${⊕}_{\tau =1}^{\overset{ˇ}{n}}M_{\tau }=1$ and 0 ≤ *M*_*τ*_ ≤ 1. Then, the SV-NHFRWG operator is described as

(115) $$\begin{array}{c} SV-NHFRWG\left(\emptyset \left(k_{1}\right),\emptyset \left(k_{2}\right),\ldots \emptyset \left(k_{\overset{ˇ}{n}}\right)\right)\text{,}\\ =\left(\overset{\overset{ˇ}{n}}{\underset{\tau =1}{⊕}}{\left(\underline{\emptyset }\left(k_{\tau }\right)\right)}^{M_{\tau }},\overset{\overset{ˇ}{n}}{\underset{\tau =1}{⊕}}{\left(\bar{\emptyset }\left(k_{\tau }\right)\right)}^{M_{\tau }}\right)\text{,}\\ =\left(\begin{array}{c} \left\{\begin{array}{c} \underset{\underline{\mu_{l_{\tau }}}\in \Delta_{l_{\underline{\emptyset }\left(k\right)}}}{\cup }\left(\overset{\overset{ˇ}{n}}{\underset{\tau =1}{⊕}}{\left(\underline{\mu_{l_{\tau }}}\right)}^{M_{\tau }}\right),\\ \underset{\underline{\lambda_{l_{\tau }}}\in \Lambda_{l_{\underline{\emptyset }\left(k\right)}}}{\cup }\left(1-\overset{\overset{ˇ}{n}}{\underset{\tau =1}{⊕}}{\left(1-\underline{\lambda_{l_{\tau }}}\right)}^{M_{\tau }}\right),\\ \underset{\underline{\nu_{l_{\tau }}}\in \nabla_{l_{\underline{\emptyset }\left(k\right)}}}{\cup }\left(1-\overset{\overset{ˇ}{n}}{\underset{\tau =1}{⊕}}{\left(1-\underline{\nu_{l_{\tau }}}\right)}^{M_{\tau }}\right) \end{array}\right\}\\ \left\{\begin{array}{c} \underset{\bar{\mu_{l_{\tau }}}\in \Delta_{l_{\bar{\emptyset }\left(k\right)}}}{\cup }\left(\overset{\overset{ˇ}{n}}{\underset{\tau =1}{⊕}}{\left(\bar{\mu_{l_{\tau }}}\right)}^{M_{\tau }}\right),\\ \underset{\bar{\lambda_{l_{\tau }}}\in \Lambda_{l_{\bar{\emptyset }\left(k\right)}}}{\cup }\left(1-\overset{\overset{ˇ}{n}}{\underset{\tau =1}{⊕}}{\left(1-{\bar{\lambda_{l}}}_{\tau }\right)}^{M_{\tau }}\right),\\ \underset{\bar{\nu_{l_{\tau }}}\in \nabla_{l_{\bar{\emptyset }\left(k\right)}}}{\cup }\left(1-\overset{\overset{ˇ}{n}}{\underset{\tau =1}{⊕}}{\left(1-{\bar{\nu_{l}}}_{\tau }\right)}^{M_{\tau }}\right) \end{array}\right\} \end{array}\right)\text{.} \end{array}$$

**Proof**

The proof is similar to the proof of Theorem 1.

**Theorem 6 .**

Let $\emptyset \left({𝕜}_{\tau }\right)=\left(\underline{\emptyset }\left({𝕜}_{\tau }\right),\bar{\emptyset }\left({𝕜}_{\tau }\right)\right)\left(\tau =1,2,3,\ldots ,\overset{ˇ}{n}\right)$ be the set of values of SV-NHFRNs with $M={\left(M_{1},M_{2},\ldots M_{\overset{ˇ}{n}}\right)}^{\delta }$ such that ${⊕}_{\tau =1}^{\overset{ˇ}{n}}M_{\tau }=1$ and 0 ≤ *M*_*τ*_ ≤ 1. Then, the SV-NHFRWG operator satisfies the following properties:

(1) Idempotency**:** If ∅(*𝕜*_*τ*_) = *ℑ*(*𝕜*), for $\tau =1,2,3,\ldots ,\overset{ˇ}{n,}$ where $ℑ\left(𝕜\right)=\left(\underline{ℑ}\left(𝕜\right),\bar{ℑ}\left(𝕜\right)\right)=\left(\left(\underline{a_{\ell \left(\Upsilon \right)}},\underline{b_{\ell \left(\Upsilon \right)}}\underline{,\underline{c_{\ell \left(\Upsilon \right)}}}\right),\left({\bar{a}}_{\ell \left(\Upsilon \right)},\bar{b_{\ell \left(\Upsilon \right)}},\bar{c_{\ell \left(\Upsilon \right)}}\right)\right)$. Then,
  (116) $$\begin{array}{c} SV-NHFRWG\left(\emptyset \left(k_{1}\right),\emptyset \left(k_{2}\right),\ldots \emptyset \left(k_{\overset{ˇ}{n}}\right)\right)=I\left(k\right)\text{.} \end{array}$$
(2) Boundedness: Let ${\left(\emptyset \left(𝕜\right)\right)}^{-}=\left(\min_{\tau }\underline{\emptyset }\left({𝕜}_{\tau }\right),\max_{\tau }\bar{\emptyset }\left({𝕜}_{\tau }\right)\right)$ and ${\left(\emptyset \left(𝕜\right)\right)}^{+}=\left(\max_{\tau }\underline{\emptyset }\left({𝕜}_{\tau }\right),\min_{\tau }\bar{\emptyset }\left({𝕜}_{\tau }\right)\right)$. Then,
  (117) $$\begin{array}{c} {\left(\emptyset \left(k\right)\right)}^{-}\leq SV-NHFRWG\left(\emptyset \left(k_{1}\right),\emptyset \left(k_{2}\right),\ldots ,\emptyset \left(k_{\overset{ˇ}{n}}\right)\right)\leq {\left(\emptyset \left(k\right)\right)}^{+}\text{.} \end{array}$$
(3) Monotonicity: Suppose $ℑ\left(𝕜\right)=\left(\underline{ℑ}\left({𝕜}_{\tau }\right),\bar{ℑ}\left({𝕜}_{\tau }\right)\right)\left(\tau =\tau ,2,\ldots ,\overset{ˇ}{n}\right)$ is another set of values of SV-NHFRNssuch that $\underline{ℑ}\left({𝕜}_{\tau }\right)\leq \underline{\emptyset }\left({𝕜}_{\tau }\right)$ and $\bar{ℑ}\left({𝕜}_{\tau }\right)\leq \bar{\emptyset }\left({𝕜}_{\tau }\right)$. Then,
  (118) $$\begin{array}{c} SV-NHFRWG\left(I\left(k_{1}\right),I\left(k_{2}\right),\ldots ,I\left(k_{\overset{ˇ}{n}}\right)\right)\leq SV-NHFRWG\left(\emptyset \left(k_{1}\right),\emptyset \left(k_{2}\right),\ldots \emptyset \left(k_{\overset{ˇ}{n}}\right)\right)\text{.} \end{array}$$
(4) Shift invariance: Let SV-NHFRN $ℑ\left(𝕜\right)=\left(\underline{ℑ}\left(𝕜\right),\bar{ℑ}\left(𝕜\right)\right)=\left(\left(\underline{b},\underline{c},\underline{d}\right),\left(\bar{b},\bar{c},\bar{d}\right)\right)$. Then,
  (119) $$\begin{array}{c} SV-NHFRWG\left(\emptyset \left(k_{1}\right)⊕I\left(k\right),\emptyset \left(k_{2}\right)⊕I\left(k\right),\ldots ,\emptyset \left(k_{\overset{ˇ}{n}}\right)⊕I\left(k\right)\right)\text{,}\\ =SV-NHFRWG\left(\emptyset \left(k_{1}\right),\emptyset \left(k_{2}\right),\ldots \emptyset \left(k_{\overset{ˇ}{n}}\right)\right)⊕I\left(k\right)\text{.} \end{array}$$
(5) Homogeneity**:** For any real number Ξ > 0,
  (120) $$\begin{array}{c} SV-NHFRWG\left(\Delta \emptyset \left(k_{1}\right),\Delta \emptyset \left(k_{2}\right),\ldots ,\Delta \emptyset \left(k_{\overset{ˇ}{n}}\right)\right)=\Delta \cdot SV-NHFRWG\left(\emptyset \left(k_{1}\right),\emptyset \left(k_{2}\right),\ldots ,\emptyset \left(k_{\overset{ˇ}{n}}\right)\right)\text{.} \end{array}$$
(6) Commutativity: Suppose $\emptyset^{\prime }\left({𝕜}_{\tau }\right)=\left(\underline{\emptyset }\left({𝕜}_{\tau }\right),\bar{\emptyset^{\prime }}\left({𝕜}_{\tau }\right)\right)$ and $\emptyset \left({𝕜}_{\tau }\right)=\left(\underline{\emptyset }\left({𝕜}_{\tau }\right),\bar{\emptyset }\left({𝕜}_{\tau }\right)\right)$, $\left(\tau =1,2,3,\ldots ,\overset{ˇ}{n}\right)$ is a set of values of SV-NHFRNs.

Then

(121) $$\begin{array}{c} SV-NHFRWG\left(\emptyset \left(k_{1}\right),\emptyset \left(k_{2}\right),\ldots ,\emptyset \left(k_{\overset{ˇ}{n}}\right)\right)=SV-NHFRWG\left(\emptyset^{\prime }\left(k_{1}\right),\emptyset^{\prime }\left(k_{2}\right),\ldots ,\emptyset^{\prime }\left(k_{\overset{ˇ}{n}}\right)\right)\text{.} \end{array}$$

**Proof**

The proof is similar to the proof of Theorem 2.

**Definition 20 .**

Let $\emptyset \left({𝕜}_{\tau }\right)=\left(\underline{\emptyset }\left({𝕜}_{\tau }\right),\bar{\emptyset }\left({𝕜}_{\tau }\right)\right)\left(\tau =1,2,3,\ldots ,\overset{ˇ}{n}\right)$ be the set of values of SV-NHFRNs with weight vector $M={\left(M_{1},M_{2},\ldots M_{\overset{ˇ}{n}}\right)}^{\delta }$ such that ${⊕}_{\tau =1}^{\overset{ˇ}{n}}M_{\tau }=1$ and 0 ≤ *M*_*τ*_ ≤ 1.

Then the SV-NHFROWG operator is described as

(122) $$\begin{array}{c} SV-NHFROWG\left(\emptyset \left(k_{1}\right),\emptyset \left(k_{2}\right),\ldots ,\emptyset \left(k_{\overset{ˇ}{n}}\right)\right)=\left({⊕}_{\tau =1}^{\overset{ˇ}{n}}{\left({\underline{\emptyset }}_{\nabla }\left(k_{\tau }\right)\right)}^{M_{\tau }},{⊕}_{\tau =1}^{\overset{ˇ}{n}}{\left(\bar{\emptyset_{\nabla }}\left(k_{\tau }\right)\right)}^{M_{\tau }}\right)\text{.} \end{array}$$

**Theorem 7 .**

Let $\emptyset \left({𝕜}_{\tau }\right)=\left(\underline{\emptyset }\left({𝕜}_{\tau }\right),\bar{\emptyset }\left({𝕜}_{\tau }\right)\right)\left(\tau =1,2,3,\ldots \overset{ˇ}{n}\right)$ be the set of values of SV-NHFRNs with $M={\left(M_{1},M_{2},\ldots M_{\overset{ˇ}{n}}\right)}^{\delta }$ such that ${⊕}_{\tau =1}^{\overset{ˇ}{n}}M_{\tau }=1$ and 0 ≤ *M*_*τ*_ ≤ 1. Then the SV-NHFROWG operator is a mapping defined as

(123) $$\begin{array}{c} SV-NHFROWG\left(\emptyset \left(k_{1}\right),\emptyset \left(k_{2}\right),\ldots \emptyset \left(k_{\overset{ˇ}{n}}\right)\right)\text{,}\\ =\left(\overset{\overset{ˇ}{n}}{\underset{\tau =1}{⊕}}{\left({\underline{\emptyset }}_{\nabla }\left(k_{\tau }\right)\right)}^{M_{\tau }},\overset{\overset{ˇ}{n}}{\underset{\tau =1}{⊕}}{\left(\bar{\emptyset_{\nabla }}\left(k_{\tau }\right)\right)}^{M_{\tau }}\right)\text{,}\\ =\left(\begin{array}{c} \left\{\begin{array}{c} \underset{\underline{\mu_{l_{\nabla_{\tau }}}}\in \Delta_{l_{\underline{\emptyset }\left(k\right)}}}{\cup }\left(\overset{\overset{ˇ}{n}}{\underset{\tau =1}{⊕}}{\left(\underline{\mu_{l_{\nabla_{\tau }}}}\right)}^{M_{\tau }}\right),\\ \underset{\underline{\lambda_{l_{\nabla_{\tau }}}}\in \Lambda_{l_{\underline{\emptyset }\left(k\right)}}}{\cup }\left(1-\overset{\overset{ˇ}{n}}{\underset{\tau =1}{⊕}}{\left(1-\underline{\lambda_{l_{\nabla_{\tau }}}}\right)}^{M_{\tau }}\right),\\ \underset{\underline{\nu_{l_{\nabla_{\tau }}}}\in \nabla_{l_{\underline{\emptyset }\left(k\right)}}}{\cup }\left(1-\overset{\overset{ˇ}{n}}{\underset{\tau =1}{⊕}}{\left(1-\underline{\nu_{l_{\nabla_{\tau }}}}\right)}^{M_{\tau }}\right) \end{array}\right\}\\ \left\{\begin{array}{c} \underset{\bar{\mu_{l_{\nabla_{\tau }}}}\in \Delta_{l_{\bar{\emptyset }\left(k\right)}}}{\cup }\left(\overset{\overset{ˇ}{n}}{\underset{\tau =1}{⊕}}{\left(\bar{\mu_{l_{\nabla_{\tau }}}}\right)}^{M_{\tau }}\right),\\ \underset{\bar{\lambda_{l_{\tau }}}\in \Lambda_{l_{\bar{\emptyset }\left(k\right)}}}{\cup }\left(1-\overset{\overset{ˇ}{n}}{\underset{\tau =1}{⊕}}{\left(1-\bar{\lambda_{l_{\nabla_{\tau }}}}\right)}^{M_{\tau }}\right),\\ \underset{\bar{\nu_{l_{\tau }}}\in \nabla_{l_{\bar{\emptyset }\left(k\right)}}}{\cup }\left(1-\overset{\overset{ˇ}{n}}{\underset{\tau =1}{⊕}}{\left(1-\bar{\nu_{l_{\nabla_{\tau }}}}\right)}^{M_{\tau }}\right) \end{array}\right\} \end{array}\right)\text{,} \end{array}$$

where $\emptyset_{\nabla }\left({𝕜}_{\tau }\right)=\left(\underline{\emptyset }\left({𝕜}_{\tau }\right),\bar{\emptyset_{\nabla_{\tau }}}\left({𝕜}_{\tau }\right)\right)$ depicts the superior value of permutation from the set of values of SV-NHFRNs.

**Proof**

The proof is similar to the proof of Theorem 1.

**Theorem 8 .**

Let $\emptyset \left({𝕜}_{\tau }\right)=\left(\underline{\emptyset }\left({𝕜}_{\tau }\right),\bar{\emptyset }\left({𝕜}_{\tau }\right)\right)\left(\tau =1,2,3,\ldots ,\overset{ˇ}{n}\right)$ be the set of values of SV-NHFRNs with $M={\left(M_{1},M_{2},\ldots M_{\overset{ˇ}{n}}\right)}^{\delta }$ such that ${⊕}_{\tau =1}^{\overset{ˇ}{n}}M_{\tau }=1$ and 0 ≤ *M*_*τ*_ ≤ 1. Then the SV-NHFROWG operator satisfies the following properties:

(1) Idempotency: If ∅(*𝕜*_*τ*_) = *ℑ*(*𝕜*) for $\tau =1,2,3,\ldots ,\overset{ˇ}{n}$, where $ℑ\left(𝕜\right)=\left(\underline{ℑ}\left(𝕜\right),\bar{ℑ}\left(𝕜\right)\right)=\left(\left(\underline{a_{\ell \left(\Upsilon \right)}},\underline{b_{\ell \left(\Upsilon \right)}},\underline{c_{\ell \left(\Upsilon \right)}}\right),\left({\bar{a}}_{\ell \left(\Upsilon \right)},\bar{b_{\ell \left(\Upsilon \right)}},\bar{c_{\ell \left(\Upsilon \right)}}\right)\right)$. Then
  (124) $$\begin{array}{c} SV-NHFROWG\left(\emptyset \left(k_{1}\right),\emptyset \left(k_{2}\right),\ldots \emptyset \left(k_{\overset{ˇ}{n}}\right)\right)=I\left(k\right)\text{.} \end{array}$$
(2) Boundedness**:** Let ${\left(\emptyset \left(𝕜\right)\right)}^{-}=\left(\min_{\tau }\underline{\emptyset }\left({𝕜}_{\tau }\right),\max_{\tau }\bar{\emptyset }\left({𝕜}_{\tau }\right)\right)$ and ${\left(\emptyset \left(𝕜\right)\right)}^{+}=\left(\max_{\tau }\underline{\emptyset }\left({𝕜}_{\tau }\right),\min_{\tau }\bar{\emptyset }\left({𝕜}_{\tau }\right)\right)$. Then
  (125) $$\begin{array}{c} {\left(\emptyset \left(k\right)\right)}^{-}\leq SV-NHFROWG\left(\emptyset \left(k_{1}\right),\emptyset \left(k_{2}\right),\ldots ,\emptyset \left(k_{\overset{ˇ}{n}}\right)\right)\leq {\left(\emptyset \left(k\right)\right)}^{+}\text{.} \end{array}$$
(3) Monotonicity**:** Let $ℑ\left(𝕜\right)=\left(\underline{ℑ}\left({𝕜}_{\tau }\right),\bar{ℑ}\left({𝕜}_{\tau }\right)\right)\left(\tau =1,2,3,\ldots ,\overset{ˇ}{n}\right)$ is another set of values of SV-NHFRNs
such that $\underline{ℑ}\left({𝕜}_{\tau }\right)\leq \underline{\emptyset }\left({𝕜}_{\tau }\right)$ and $\bar{ℑ}\left({𝕜}_{\tau }\right)\leq \bar{\emptyset }\left({𝕜}_{\tau }\right)$. Then
  (126) $$\begin{array}{c} SV-NHFROWG\left(I\left(k_{1}\right),I\left(k_{2}\right),\ldots ,I\left(k_{\overset{ˇ}{n}}\right)\right)\leq SV-NHFROWG\left(\emptyset \left(k_{1}\right),\emptyset \left(k_{2}\right),\ldots \emptyset \left(k_{\overset{ˇ}{n}}\right)\right)\text{.} \end{array}$$
(4) Shift invariance: Let SV-NROPHFRV
  (127) $$\begin{array}{c} I\left(k\right)=\left(\underline{I}\left(k\right),\bar{I}\left(k\right)\right)=\left(\left(\underline{a_{l\left(\Upsilon \right)}},\underline{b_{l\left(\Upsilon \right)}},\underline{c_{l\left(\Upsilon \right)}}\right),\left({\bar{a}}_{l\left(\Upsilon \right)},\bar{b_{l\left(\Upsilon \right)}},\bar{c_{l\left(\Upsilon \right)}}\right)\right)\text{.} \end{array}$$
Then
  (128) $$\begin{array}{c} SV-NHFROWG\left(\emptyset \left(k_{1}\right)⊕I\left(k\right),\emptyset \left(k_{2}\right)⊕I\left(k\right),\ldots ,\emptyset \left(k_{\overset{ˇ}{n}}\right)⊕I\left(k\right)\right)\text{,}\\ =SV-NHFROWG\left(\emptyset \left(k_{1}\right),\emptyset \left(k_{2}\right),\ldots \emptyset \left(k_{\overset{ˇ}{n}}\right)\right)⊕I\left(k\right)\text{.} \end{array}$$
(5) Homogeneity: For any real number Δ > 0,
  (129) $$\begin{array}{c} SV-NHFROWG\left(\Delta \emptyset \left(k_{1}\right),\Delta \emptyset \left(k_{2}\right),\ldots ,\Delta \emptyset \left(k_{\overset{ˇ}{n}}\right)\right)=\Delta \cdot SV-NHFROWG\left(\emptyset \left(k_{1}\right),\emptyset \left(k_{2}\right),\ldots ,\emptyset \left(k_{\overset{ˇ}{n}}\right)\right)\text{.} \end{array}$$
(6) Commutativity: Let $\emptyset^{\prime }\left({𝕜}_{\tau }\right)=\left(\underline{\emptyset }\left({𝕜}_{\tau }\right),\bar{\emptyset^{\prime }}\left({𝕜}_{\tau }\right)\right)$ and $\emptyset \left({𝕜}_{\tau }\right)=\left(\underline{\emptyset }\left({𝕜}_{\tau }\right),\bar{\emptyset }\left({𝕜}_{\tau }\right)\right)$, $\left(\tau =1,2,3,\ldots ,\overset{ˇ}{n}\right)$ is a set of values of SV-NHFRNs.

Then

(130) $$\begin{array}{c} SV-NHFROWG\left(\emptyset \left(k_{1}\right),\emptyset \left(k_{2}\right),\ldots ,\emptyset \left(k_{\overset{ˇ}{n}}\right)\right)=SV-NHFROWG\left(\emptyset^{\prime }\left(k_{1}\right),\emptyset^{\prime }\left(k_{2}\right),\ldots ,\emptyset^{\prime }\left(k_{\overset{ˇ}{n}}\right)\right)\text{.} \end{array}$$

**Proof**

The proof is similar to the proof of Theorem 2.

## 6. Multiattribute Decision-Making Methodology

Here, we developed an algorithm to inscribe uncertainty in MAGDM under SV-NHFR details/informations. Assume a DM challenge with a set $\left\{\Xi_{1},\Xi_{2},\ldots \ldots ,\Xi_{\overset{ˇ}{n}}\right\}$ of $\overset{ˇ}{n}$ alternatives and a set of $\overset{ˇ}{n}$ attributes $\left\{P_{1},P_{2},\ldots .,P_{\overset{ˇ}{n}}\right\}$ with ${\left(M_{1},M_{2},\ldots ..,M_{\overset{ˇ}{n}}\right)}^{\delta }$ the weights, that is, *M*_*τ*_ ∈ [0,1] and ${⊕}_{\tau =1}^{\overset{ˇ}{n}}M_{\tau }=1$. To test the reliability of kth alternative Ξ_*τ*_ under the the attribute *P*_*τ*_, let {D_1_, D_2_,…., D} be a set of decision-makers (DMs) and ${\left({℧}_{1},{℧}_{2},\ldots .,{℧}_{\overset{ˇ}{n}}\right)}^{\delta }$ be DMs weights such that ℧_*τ*_ ∈ [0,1] and ${⊕}_{\tau =1}^{\overset{ˇ}{n}}{℧}_{\tau }=1$. The expert evaluation matrix is

(131) $$\begin{array}{c} M={\left[\bar{\emptyset }\left(k_{\tau j}\right)\right]}_{m\times \overset{ˇ}{n}}\text{,}\\ =\left[\begin{array}{cccc} \left(\bar{\emptyset }\left(k_{11}\right),\underline{\emptyset }\left(k_{11}\right)\right) & \left(\bar{\emptyset }\left(k_{12}\right),\underline{\emptyset }\left(k_{12}\right)\right) & \cdots & \left(\bar{\emptyset }\left(k_{1j}\right),\underline{\emptyset }\left(k_{1j}\right)\right)\\ \left(\bar{\emptyset }\left(k_{21}\right),\underline{\emptyset }\left(k_{21}\right)\right) & \left(\bar{\emptyset }\left(k_{22}\right),\underline{\emptyset }\left(k_{22}\right)\right) & \cdots & \left(\bar{\emptyset }\left(k_{2j}\right),\underline{\emptyset }\left(k_{2j}\right)\right)\\ \left(\bar{\emptyset }\left(k_{31}\right),\underline{\emptyset }\left(k_{31}\right)\right) & \left(\bar{\emptyset }\left(k_{32}\right),\underline{\emptyset }\left(k_{32}\right)\right) & \cdots & \left(\bar{\emptyset }\left(k_{3j}\right),\underline{\emptyset }\left(k_{3j}\right)\right)\\ \vdots & \vdots & \ddots & \vdots \\ \left(\bar{\emptyset }\left(k_{\tau 1}\right),\underline{\emptyset }\left(k_{\tau 1}\right)\right) & \left(\bar{\emptyset }\left(k_{\tau 2}\right),\underline{\emptyset }\left(k_{\tau 2}\right)\right) & \cdots & \left(\bar{\emptyset }\left(k_{\tau j}\right),\underline{\emptyset }\left(k_{\tau j}\right)\right) \end{array}\right]\text{,} \end{array}$$

where

(132) $$\begin{array}{c} \bar{\emptyset }\left(k_{\tau j}\right)=\left\{\langle \Xi ,\Delta_{l_{\bar{\emptyset }\left(k\right)}}\left(\Xi \right),\Lambda_{l_{\bar{\emptyset }\left(k\right)}}\left(\Xi \right),\nabla_{l_{\bar{\emptyset }\left(k\right)}}\left(\Xi \right)\rangle |\Xi \in I\right\}\text{,} \end{array}$$

and

(133) $$\begin{array}{c} \underline{\emptyset }\left(k\right)=\left\{\langle \Xi ,\Delta_{l_{\underline{\emptyset }\left(k\right)}}\left(\Xi \right),\Lambda_{l_{\underline{\emptyset }\left(k\right)}}\left(\Xi \right),\nabla_{l_{\underline{\emptyset }\left(k\right)}}\left(\Xi \right)\rangle |\Xi \in I\right\}\text{,} \end{array}$$

such that

(134) $$\begin{array}{c} 0<\left(\max\;\left(\Delta_{l_{\bar{\emptyset }\left(k\right)}}\left(\Xi \right)\right)\right)+\left(\min\;\left(\Delta_{l_{\bar{\emptyset }\left(k\right)}}\left(\Xi \right)\right)\right)+\left(\min\;\left(\nabla_{l_{\bar{\emptyset }\left(k\right)}}\left(\Xi \right)\right)\right)\leq 3\text{,} \end{array}$$

and

(135) $$\begin{array}{c} 0<\left(\left.\min\;\left(\Delta_{l_{\underline{\emptyset }\left(k\right)}}\right.\left(\Xi \right.\right)\right)+\left(\max\;\left(\Delta_{l_{\underline{\emptyset }\left(k\right)}}\left(\Xi \right)\right)\right)+\left(\max\;\left(\nabla_{l_{\underline{\emptyset }\left(k\right)}}\left(\Xi \right)\right)\right)\leq 3, \end{array}$$

are the SV-NHF rough values. The main steps for MAGDM are as follows:

*Step 1*. Construct the expert evaluation matrices as
  (136) $$\begin{array}{c} {\left(D\right)}^{\overset{\wedge }{\text{í}}}=\left[\begin{array}{cccc} \left(\bar{\emptyset }\left(k_{11}^{\overset{\wedge }{\text{í}}}\right),\underline{\emptyset }\left(k_{11}^{\overset{\wedge }{\text{í}}}\right)\right) & \left(\bar{\emptyset }\left(k_{12}^{\overset{\wedge }{\text{í}}}\right),\underline{\emptyset }\left(k_{12}^{\overset{\wedge }{\text{í}}}\right)\right) & \cdots & \left(\bar{\emptyset }\left(k_{1j}^{\overset{\wedge }{\text{í}}}\right),\underline{\emptyset }\left(k_{1j}^{\overset{\wedge }{\text{í}}}\right)\right)\\ \left(\bar{\emptyset }\left(k_{21}^{\overset{\wedge }{\text{í}}}\right),\underline{\emptyset }\left(k_{21}^{\overset{\wedge }{\text{í}}}\right)\right) & \left(\bar{\emptyset }\left(k_{22}^{\overset{\wedge }{\text{í}}}\right),\underline{\emptyset }\left(k_{22}^{\overset{\wedge }{\text{í}}}\right)\right) & \cdots & \left(\bar{\emptyset }\left(k_{2j}^{\overset{\wedge }{\text{í}}}\right),\underline{\emptyset }\left(k_{2j}^{\overset{\wedge }{\text{í}}}\right)\right)\\ \left(\bar{\emptyset }\left(k_{31}^{\overset{\wedge }{\text{í}}}\right),\underline{\emptyset }\left(k_{31}^{\overset{\wedge }{\text{í}}}\right)\right) & \left(\bar{\emptyset }\left(k_{32}^{\overset{\wedge }{\text{í}}}\right),\underline{\emptyset }\left(k_{32}^{\overset{\wedge }{\text{í}}}\right)\right) & \cdots & \left(\bar{\emptyset }\left(k_{3j}^{\overset{\wedge }{\text{í}}}\right),\underline{\emptyset }\left(k_{3j}^{\overset{\wedge }{\text{í}}}\right)\right)\\ \vdots & \vdots & \vdots & \vdots \\ \left(\bar{\emptyset }\left(k_{\tau 1}^{\text{í}}\right),\underline{\emptyset }\left(k_{\tau 1}^{\overset{\wedge }{\text{í}}}\right)\right) & \left(\bar{\emptyset }\left(k_{\tau 2}^{\overset{\wedge }{\text{í}}}\right),\underline{\emptyset }\left(k_{\tau 2}^{\overset{\wedge }{\text{í}}}\right)\right) & \cdots & \left(\bar{\emptyset }\left(k_{\tau j}^{\overset{\wedge }{\text{í}}}\right),\underline{\emptyset }\left(k_{\tau j}^{\overset{\wedge }{\text{í}}}\right)\right) \end{array}\right]\text{,} \end{array}$$
where $\overset{\wedge }{\text{í}}$ shows the number of expert
*Step 2*. Evaluate the normalized experts matrices ${\left(\mathbb{N}\right)}^{\overset{\wedge }{\text{í}}}$ as
  (137) $$\begin{array}{c} {\left(N\right)}^{\overset{\wedge }{\text{í}}}=\left\{\begin{array}{cc} \emptyset \left(k_{\tau j}\right)=\left(\underline{\emptyset }\left(k_{\tau j}\right),\bar{\emptyset }\left(k_{\tau j}\right)\right), & if\;For\;benefit,\\ {\left(\emptyset \left(k_{\tau j}\right)\right)}^{c}=\left({\left(\underline{\emptyset }\left(k_{\tau j}\right)\right)}^{c},{\left(\bar{\emptyset }\left(k_{\tau j}\right)\right)}^{c}\right), & if\;For\;cost. \end{array}\right. \end{array}$$
*Step 3*. Compute the collected SV-neutrosophic fuzzy rough information of decision-makers using the SV-NHFRWA and SV-NHFRWG aggregation operators as
  (138) $$\begin{array}{c} SV-NHFRWA\left(\emptyset \left(k_{1}\right),\emptyset \left(k_{2}\right),\ldots \emptyset \left(k_{\overset{ˇ}{n}}\right)\right)=\left(\overset{\overset{ˇ}{n}}{\underset{\tau =1}{⊕}}M_{\tau }\underline{\emptyset }\left(k_{\tau }\right),\overset{\overset{ˇ}{n}}{\underset{\tau =1}{⊕}}M_{\tau }\bar{\emptyset }\left(k_{\tau }\right)\right)\left[\begin{array}{c} \left(\begin{array}{c} \underset{\underline{\mu_{l_{\tau }}}\in \Delta_{l_{\underline{\emptyset }\left(k\right)}}}{\cup }\left(1-\overset{\overset{ˇ}{n}}{\underset{\tau =1}{⊕}}{\left(1-\underline{\mu_{l_{\tau }}}\right)}^{M_{\tau }}\right),\\ \underset{\underline{\lambda_{l_{\tau }}}\in \Lambda_{l_{\underline{\emptyset }\left(k\right)}}}{\cup }\left(\overset{\overset{ˇ}{n}}{\underset{\tau =1}{⊕}}{\left(\underline{\lambda_{l_{\tau }}}\right)}^{M_{\tau }}\right)\\ \underset{\underline{\nu_{l_{\tau }}}\in \nabla_{l_{\underline{\emptyset }\left(k\right)}}}{\cup }\left(\overset{\overset{ˇ}{n}}{\underset{\tau =1}{⊕}}{\left(\underline{\nu_{l_{\tau }}}\right)}^{M_{\tau }}\right) \end{array}\right)\\ \left(\begin{array}{c} \underset{\bar{\mu_{l_{\tau }}}\in \Delta_{l_{\bar{\emptyset }\left(k\right)}}}{\cup }\left(1-\overset{\overset{ˇ}{n}}{\underset{\tau =1}{⊕}}{\left(1-\bar{\mu_{l_{\tau }}}\right)}^{M_{\tau }}\right),\\ \;\underset{\bar{\lambda_{l_{\tau }}}\in \Lambda_{l_{\bar{\emptyset }\left(k\right)}}}{\cup }\left(\overset{\overset{ˇ}{n}}{\underset{\tau =1}{⊕}}{\left(\bar{\lambda_{l_{\tau }}}\right)}^{M_{\tau }}\right)\\ \;\underset{\bar{\nu_{l_{\tau }}}\in \nabla_{l_{\bar{\emptyset }\left(k\right)}}}{\cup }\left(\overset{\overset{ˇ}{n}}{\underset{\tau =1}{⊕}}{\left(\bar{\nu_{l_{\tau }}}\right)}^{M_{\tau }}\right) \end{array}\right) \end{array}\right]sV-NHFROWG\left(\emptyset \left(k_{1}\right),\emptyset \left(k_{2}\right),\ldots \emptyset \left(k_{\overset{ˇ}{n}}\right)\right)=\left(\overset{\overset{ˇ}{n}}{\underset{\tau =1}{⊕}}{\left({\underline{\emptyset }}_{\nabla }\left(k_{\tau }\right)\right)}^{M_{\tau }},\overset{\overset{ˇ}{n}}{\underset{\tau =1}{⊕}}{\left(\bar{\emptyset_{\nabla }}\left(k_{\tau }\right)\right)}^{M_{\tau }}\right)\left(\begin{array}{c} \left\{\begin{array}{c} \underset{\underline{\mu_{l_{\nabla_{\tau }}}}\in \Delta_{l_{\underline{\emptyset }\left(k\right)}}}{\cup }\left(\overset{\overset{ˇ}{n}}{\underset{\tau =1}{⊕}}{\left(\underline{\mu_{l_{\nabla_{\tau }}}}\right)}^{M_{\tau }}\right),\\ \underset{\underline{\lambda_{l_{\nabla_{\tau }}}}\in \Lambda_{l_{\underline{\emptyset }\left(k\right)}}}{\cup }\left(1-\overset{\overset{ˇ}{n}}{\underset{\tau =1}{⊕}}{\left(1-\underline{\lambda_{l_{\nabla_{\tau }}}}\right)}^{M_{\tau }}\right),\\ \underset{\underline{\nu_{l_{\nabla_{\tau }}}}\in \nabla_{l_{\underline{\emptyset }\left(k\right)}}}{\cup }\left(1-\overset{\overset{ˇ}{n}}{\underset{\tau =1}{⊕}}{\left(1-\underline{\nu_{l_{\nabla_{\tau }}}}\right)}^{M_{\tau }}\right) \end{array}\right\}\\ \left\{\begin{array}{c} \underset{\bar{\mu_{l_{\nabla_{\tau }}}}\in \Delta_{l_{\bar{\emptyset }\left(k\right)}}}{\cup }\left(\overset{\overset{ˇ}{n}}{\underset{\tau =1}{⊕}}{\left(\bar{\mu_{l_{\nabla_{\tau }}}}\right)}^{M_{\tau }}\right),\\ \underset{\bar{\lambda_{l_{\tau }}}\in \Lambda_{l_{\bar{\emptyset }\left(k\right)}}}{\cup }\left(1-\overset{\overset{ˇ}{n}}{\underset{\tau =1}{⊕}}{\left(1-\bar{\lambda_{l_{\nabla_{\tau }}}}\right)}^{M_{\tau }}\right),\\ \underset{\bar{\nu_{l_{\tau }}}\in \nabla_{l_{\bar{\emptyset }\left(k\right)}}}{\cup }\left(1-\overset{\overset{ˇ}{n}}{\underset{\tau =1}{⊕}}{\left(1-\bar{\nu_{l_{\nabla_{\tau }}}}\right)}^{M_{\tau }}\right) \end{array}\right\} \end{array}\right)\text{.} \end{array}$$
*Step 4*. Evaluate the aggregated SV-NHFRNs for each considered alternative with respect to the given list of criteria/attributes by utilizing the proposed aggregation information
*Step 5*. Find the ranking of alternatives based on score function as
  (139) $$\begin{array}{c} \Delta \left(\emptyset \left(k\right)\right)=\frac{1}{6}\left(\begin{array}{c} 3+\left(\frac{1}{Z_{\Omega }}\underset{\underline{\mu_{l_{\tau }}}\in \Delta_{l_{\underline{\emptyset }\left(k\right)}}}{\sum }\left(\underline{\mu_{l_{\tau }}}\right)+\frac{1}{Y_{\Omega }}\underset{\bar{\mu_{l_{\tau }}}\in \Delta_{l_{\bar{\emptyset }\left(k\right)}}}{\sum }\left(\bar{\mu_{l_{\tau }}}\right)\right)-\\ \left(\frac{1}{Z_{\Omega }}\underset{\underline{\lambda_{l_{\tau }}}\in \Lambda_{l_{\underline{\emptyset }\left(k\right)}}}{\sum }\left(\underline{\lambda_{l_{\tau }}}\right)-\frac{1}{Y_{\Omega }}\underset{\bar{\lambda_{l_{\tau }}}\in \Lambda_{l_{\bar{\emptyset }\left(k\right)}}}{\sum }\left(\bar{\lambda_{l_{\tau }}}\right)\right)-\\ \left(\frac{1}{Z_{\Omega }}\underset{\underline{\nu_{l_{\tau }}}\in \nabla_{l_{\underline{\emptyset }\left(k\right)}}}{\sum }\left(\underline{\nu_{l_{\tau }}}\right)-\frac{1}{Y_{\Omega }}\underset{\bar{\nu_{l_{\tau }}}\in \nabla_{l_{\bar{\emptyset }\left(k\right)}}}{\sum }\left(\bar{\nu_{l_{\tau }}}\right)\right) \end{array}\right). \end{array}$$
*Step 6*. Rank all the alternative scores in the descending order. The alternatives having larger value will be superior/best

### 6.1. Numerical Example

We provided a multiattribute approach for evaluating the sustainability factor for industry based on IoT in this part, which integrates under SV-neutrosophic hesitant fuzzy rough information. We consider a numerical illustration of determining the right option that helps to achieve the manufacturing industry sustainability based on technology using proposed SV-NHFR aggregation information.

The following four alternatives depend upon to achieve the sustainability for manufacturing industry.

#### 6.1.1. Case Study (Sustainability of Manufacturing Industry)

The article discusses manufacturing industry aspects that will aid in the achievement of long-term manufacturing sustainable goals. All of these elements are impossible to accomplish for a manufacturer. Our goal is to ensure long-term viability. We categorize the best option that will be more advantageous to reaching sustainability goals based on the findings of this study. We choose four option such as: waste management and product waste reduction, environmental protection and social welfare, manufacturing cost savings and efficiency, and chemicals and resources. Four criteria are used to each choice. Here four alternatives are as given in the next section:

#### 6.1.2. Waste Management and Reduce Product Waste

Reducing the amount of solid waste deposited or burnt is a primary objective of sustainable waste management. People who do not properly dispose garbage contribute to climate change and air pollution while also damaging ecosystems and species around the globe. A potent greenhouse gas connected to climate change, methane, is produced by landfills and is the last resort in the waste hierarchy. Industrial operations, economic growth, and population growth have resulted in a rise in trash creation on a daily basis.

Agricultural, commercial, industrial, and municipal waste can all be classified as waste. As an obstacle to urban sustainability, environmental issues necessitate the development of an innovative approach for tracking their dynamics across numerous regions and countries. In spite of the fact that this is a widely used approach for municipal waste disposal across the world, the reader will not discover in-depth talks on the matter. Accordingly, landfilling and the many treatment methods that stabilize hazardous waste are not cost-effective even if they allow businesses and governments to comply with environmental rules. Disposal and treatment technologies necessitate substantial up-front and continuing financial commitments. However, waste and pollution are still being managed and disposed of, posing continuing and future threats to the population and environment.

This section lays the basis for alternatives to so-called end-of-pipe treatment and disposal methods. Some of the terminologies used to characterize these possibilities are waste reduction, waste-to-energy, resource recovery and reuse, and recycling. Although a better general name or phrase to cover all of these alternative methods may exist, we will use the term prevention or P2 again, bearing in mind that it is not always used in the strictest sense of source reduction.

Furthermore, no difference is made, if any, between waste and pollution. Pollution is a by-product. In an ideal society, processes would be completely efficient, and customers would have no undesired or worn-out items to dispose. However, all industrial activities produce nonvalue-added by-products, and consumer goods have throwaway packaging and limited lifespans. These sorts of solid waste are just a loss of money due to inefficiencies in the industry and cultural practices. This book focuses on recovering and controlling financial losses in order to enhance industrial and public environmental performance.

#### 6.1.3. Environmental Protection and Social Welfare

Environmental and social sustainability refers to the adoption and incorporation of precautionary environmental and social concepts and variables into decision-making processes (ESS). The adaptation and integration of precautionary environmental and social principles and factors into decision-making processes is known as Environmental and Social Sustainability (ESS). UN organizations across the system are dedicated to internalizing the concepts they stand for—leading by example and retaining credibility as a partner in sustainable development—as part of the UN's commitment to promote nationally owned sustainable development results. Enhancing the UN's environmental and social sustainability is an important part of this. Policy makers are frequently forced to select between various environmental policy instruments.

The impact of various policies on company incentives to adopt cleaner manufacturing technologies is a crucial factor influencing this decision. In the long run, technological innovation's cumulative effect may considerably alleviate what may appear to be serious conflicts between economic activity and environmental quality in the short term. This impact is particularly relevant in the context of global climate change as governments have been hesitant to implement policies to significantly cut greenhouse gas emissions due to the possible economic consequences. Environmental economics includes a strand of literature that looks at the influence of environmental policies on technological innovation. This literature is mostly theoretical. In a single-firm scenario, several early studies found that emission fees and emission permits give greater incentives for technological innovation than “command and control” policies (such as performance requirements and technology mandates). Many ideas, on the other hand, are applicable to more than one company. The spillover advantages of innovation to other businesses, as well as inventors' incapacity to completely collect the rents from invention, are at the heart of most R&D models in the industrial organizations literature. As a result, more recent environmental economics research studies have enlarged prior models to include the spread of innovative technology to other enterprises in the industry.

#### 6.1.4. Manufacturing Cost Savings and Efficiency

Sustainable practices and green manufacturing are two ways for cutting long-term costs and enhancing efficiency. They go beyond the simple cost-cutting strategies employed by many companies. Going green and implementing sustainable practices are long-term investments in the profitability of a business. They require a paradigm shift in planning, but the benefits are well worth the effort. Before we get into the benefits, let us go over the definitions of green manufacturing and sustainable processes. First, the quantity of emissions abatement is bigger under the emissions tax than it is under the emissions permits following innovation. With tax, innovation lowers the (marginal) cost of emissions reduction, inspiring additional reduction, but with permits, the amount of emissions at the industry level is fixed by definition. Businesses are willing to spend more for innovations that cut abatement costs because the tax reduces emissions by a greater amount. The reduction in abatement costs induced by industry-level innovation is referred to as the abatement cost impact.

As a result, emissions tax has a bigger abatement cost effect than emissions permit. The impact of innovation on the equilibrium permit price causes the second effect. Firms benefit from cheaper permit prices if they buy enough permits to cover their emissions, which they do via auctioned permits. The emissions payment impact refers to the reduction in payments on company emissions caused by innovation. In the case of a constant emissions tax and (in the aggregate) free permits, this effect is missing. When compared to the emissions tax, the effect of the emissions payment in MP is frequently significant to increase the overall incentives for innovation in auctioned permits. Our article differs from Milliman and Prince (MP) in three significant areas. To begin, some assumptions regarding the adoption process and the spillover mechanism are altered. MP implies that inventors can allocate a predetermined percentage of the private gains from a new technology to all industry businesses. In our research, we assume a competitive equilibrium in which noninnovating businesses pay a royalty for the new technology. The royalty level is determined endogenously by the innovator's desire to get payment from the marginal, noninnovating enterprise. A key corollary of this assumption in a permit system is that the innovator cannot steal any of the emission payment impact going to noninnovators because the marginal enterprise has no effect on the equilibrium permit price.

#### 6.1.5. Chemicals and Resources

Chemical reactions is a fantastic tool for discovering novel products that can be used in future developments. Chemical reactions take a lot of time and money since we have to buy a lot of chemicals and their instruments, and sometimes toxic products are formed that hurt us and our environment. Purchase only as much chemical as you require. Use up your household chemicals and items before they go bad or expire. Give free paint and chemicals to individuals who will actually utilize them. Automobile fluid should be recycled, reused, or donated.List of the criteria that are helpful to create sustainibility.

#### 6.1.6. Efficient Inventory Management

This conclusion implies that as manufacturing enterprises improve their inventory management of raw materials, their profitability rises. About 1% improvement in raw materials inventory management will result in a 9.35% gain in profitability. The relationship has a 93.5% strength rating. Reduce, reuse, and recycle—these three “*R*” words are crucial to living a sustainable lifestyle since they assist to reduce the amount of garbage we produce. It is a piece of cake! Reduce how much waste you generate. Before you replace something, try to reuse it as much as possible. Patagonia, an outdoor clothing manufacturer, is consistently ranked at the top of eco-friendly company lists, and with good reason. Throughout its 47-year history, the Ventura-based company has been at the forefront of ecologically friendly corporate practices. For long-term sustainability, reducing transportation's environmental impact is a potential option. Transportation exacerbates emissions, noise, and climate change. Transportation accounts for approximately 15% of total greenhouse gas emissions and 22% of CO2 emissions.

#### 6.1.7. Economic Viability (Market Capacity, Capacity of Businesses to Supply, Levels of Demand for the Required Service)

When seen in the context of society as a whole, a project is economically viable if its economic advantages outweigh its economic costs. The project's economic costs are not the same as its financial cost and; externalities and environmental implications must be taken into account. Currently, the green economy is valued as much as the fossil fuel sector, with clean energy, energy efficiency, water, waste, and pollution services accounting for 6% of the world stock market, or around $4 trillion USD. Many of the most pressing environmental stressors and social conflicts highlighted by the United Nations' Sustainable Development Goals are exacerbated by global supply networks (SDGs). Companies are implementing a number of voluntary procedures to improve the environmental and/or social management of their suppliers' activities in response to concerns from the worldwide community. Those six critical factors, according to Foundry, are optimizing current fossil fuel use, eliminating waste, recycling, recovering energy, saving time, and reducing or eliminating pollution. All of these seem nice, but you can tell they are more concerned with revenues and image than with environmental concerns.

#### 6.1.8. Adopting Lean Manufacturing Principles

As most businesses are beginning to realize, the quest to go green takes them back to lean manufacturing. Lean principles call for a systematic approach to identifying and eliminating waste while striving for continuous improvement. One of the most essential ways to improve environmental performance is to use this strategy. Many evidences imply that lean manufacturing is helpful for long-term production, particularly from an environmental and economic standpoint. This article identifies important research gaps for integrated lean and sustainable manufacturing, as well as modeling as a methodology approach to improve business performance. There are five fundamental lean principles according to Womack and Jones: value, value stream, flow, pull, and perfection.

#### 6.1.9. Air and Soil Pollution

Pollutants in the air have a deleterious effect on environmental growth primarily by interfering with the accumulation of resources. Air pollution has a number of effects on crops and trees. Ground-level ozone can diminish agricultural crop and commercial forest yields, stunt tree seedling growth and survival, and make plants more susceptible to disease, pests, and other environmental challenges (such as harsh weather). In addition to harming our air and water, the soil suffers significant damage, which, if not addressed, could eventually result in the inability to produce healthy foods. The poorest countries and those with abundant mineral resources will be the hardest hit. Loss of soil productivity and agricultural yields, contamination of food goods and loss of marketability, degradation of biodiversity, and reduction of water quality are all quantifiable economic losses caused by soil pollution.

Suppose an industrialization department wants sustainability in manufacturing industry that is based on IoT. The department will invite a panel of experts to analyze a number of alternatives where each alternative depends upon the same criteria. Let {Ξ_1_, Ξ_2_, Ξ_3_, Ξ_4_} be the set of alternatives that create sustainability in manufacturing industry and {*P*_1_, *P*_2_, *P*_3_, *P*_4_} be the selection criteria defined by the experts evaluation team established by the industrialization department which are cyber risk management (*P*_1_), software supply chain security (*P*_2_), IoT fingerprinting (*P*_3_), and IoT lifecycle management (*P*_4_). Each criterion is evaluated by three different components. To determine the best alternative that creates sustainability for manufacturing industry based on the list of criteria *P*_1_, *P*_2_, *P*_3_, and *P*_4_, the industrialization department invites a group of professionals to examine the four alternatives each based on four evaluation criteria. Because of the uncertainty, the DMs' selection information is presented as SV-NHFR information. The weight vector for criteria is *M* = (0.2,0.3,0.1,0.4)^*δ*^. To provide foundational understanding on how industry technologies connect to sustainability, we use secondary data from a World Economic Forum White Paper. To solve the MCDM problem using the developed methodology for evaluating alternatives, calculations are performed as given in the following part.

**Figure 1:**
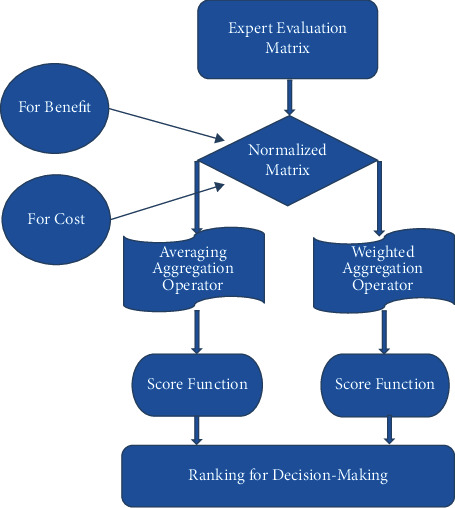
Flowchart of the algorithm.

The flowchart of the algorithm is shown in Figure 1. Table 1-11

Step 1: The information of professional expert is given in Tables 2–5 in the form of SV-NHFRS.
Step 2: Expert opinion is of the beneficiary type. As a result, we do not need to normalize the SV-NHFRNs in this context.
Step 3: Only one expert is considered in this problem for the collection of uncertain information. As a result, we are not required to detect the obtained information.
Step 4: The following aggregation operators are used to evaluate the alternative's aggregation details under the given list of attributes:
    Case-1: Tables 6 and 7 show the results of aggregation using the weighted averaging operator.
    Case-2: Aggregation information using SV-NHFRWG operator is presented in Tables 8 and 9.
Step 5: Score values of all alternatives under developed aggregation operators are presented in Table 10.
Step 6: Ranked alternatives Ξ_*k*_(*k* = 1,2,…., 4) are enclosed in Table 11.

From the above computational process, we concluded that alternative Ξ_2_ is the finest alternative among others, and therefore, it is highly recommended.

## 7. Reliability and Validity Test

Choosing the best choice from the group's evaluation criteria is a difficult process in reality. Wang and Triantaphyllou proposed a method for evaluating the reliability and validity of DM systems [34]. The following is the testing technique.

 
**Test Step 1**: The proper and successful MAGDM approach is to substitute the normalized element for the poorer element of the alternative by providing the appropriate alternative with no change and also without changing the similar position of each choice criterion.
 
**Test Step 2**: The transitive property must be fulfilled using a MAGDM technique that is both efficient and accurate.
 
**Test Step 3**: When a major MAGDM issue is reduced to a minor issue, the merged alternative rating should be similar to the undecomposed problem's original rating. We use the same approaches used in the MAGDM problem on minor issues to rank the alternative.

In order to attain the best result, the MAGDM issue was decreased to a smaller one, and the same proposed DM approach was applied. The appropriate and effective MAGDM technique is that we can use the same approach to a minor problem and get the same outcome as with the MAGDM problem.

### 7.1. Validity Test of the Proposed DM Methodology

In this section [34], we examine the adequacy and validity of our established strategy using the proficiency and validity of the above mentioned test. The *SV*-NHFR information is enclosed in Table 11.

#### 7.1.1. Test Step 1

In this stage, we replace the poorer part of the alternative by providing the appropriate alternative with no changes to the comparable positions of each selection criterion. Tables 12–15 enclosed the updated decision matrix

Now, we calculate the combined preference values of each alternative under criteria weight *M* = (0.2,0.3,0.1,0.4)^*δ*^ using proposed list of *SV*-neutrosophic probabilistic hesitant fuzzy rough aggregation operators as follows:

  Case-I: Aggregated information using *SV*-NHFRWA operators is enclosed in Tables 16 and 17
  Case-II: Updated aggregated information using *SV*-NHFRWG operators is enclosed in Tables 18 and 19

Score values of all alternatives under developed aggregation operators are presented in Table 20.

Ranked alternatives Ξ_*k*_(*k* = 1,2,…., 4) are enclosed in Table 21:

We get again the same alternative Ξ_1_ by using the test step 1, which is also obtained by applying of our suggested method.

*Test Steps 2 and 3*. We are now testing the validity test steps 2 and 3 to demonstrate that the proposed approach is reliable and relevant. To this end, we first transformed the MAGDM problem into three smaller subproblems such as {Ξ_2_, Ξ_4_, Ξ_1_}, {Ξ_4_, Ξ_1_, Ξ_3_}, and {Ξ_2_, Ξ_4_, Ξ_3_}. We now implement our suggested decision-making approach to the smaller problems that have been transformed and give us the ranking of alternatives as Ξ_2_>Ξ_4_ > Ξ_1_, Ξ_4_>Ξ_1_ > Ξ_3_, and Ξ_2_ > Ξ_4_ > Ξ_3_, respectively. We analyzed that Ξ_2_ > Ξ_4_ > Ξ_1_ > Ξ_3_ is the same as the standard decision-making approach results [35] when assigning a detailed ranking. [36–39]

## 8. Conclusion

The technique of SV-NHFRS was established under the SV-neutrosophic set, hesitant fuzzy set, and rough set environment to allow additional flexibility for decision-makers in MAGDM challenges by qualitatively defining the evaluation values. A SV-NHFR model, in contrast to traditional fuzzy models, can tackle real-world problems involving inconsistency, imprecision, and imperfection.

We introduced the SV-NHFRWA and SV-NHFRWG aggregation operators, which are efficient and adaptable operators for dealing with MCGDM situations involving uncertainty.

Furthermore, an example is given to maintain the sustainability for a manufacturing industry to illustrate the technique's authenticity and efficacy. As a result, the suggested SV-NHFR multicriteria decision-making method is better suited to real-world scientific and engineering applications since it can manage not only incomplete but also indeterminate and inconsistent data, both of which are typical in real-world scenarios. The strategy suggested in this paper complements existing decision-making techniques and provides a helpful tool for decision-makers. Some crucial subjects are still present in terms of potential future works that are good enough to justify.

Our research will be expanded in the future to include the following: (1) SV-neutrosophic hesitant fuzzy rough ordered weighted averaging operator; (2) SV-neutrosophic hesitant fuzzy rough hybrid weighted averaging operator; (3) SV-neutrosophic hesitant fuzzy rough ordered weighted geometric operator; (4) SV-neutrosophic soft hesitant fuzzy rough hybrid weighted averaging operator; (5) SV-neutrosophic hyper soft hesitant fuzzy rough hybrid weighted averaging operator and other more extensions on this set.

## Figures and Tables

**Table 1:** SV-NHFR relation in *E*.

*𝕜*	${\overset{\wedge }{C}}_{1}$	${\overset{\wedge }{C}}_{2}$	${\overset{\wedge }{C}}_{3}$	${\overset{\wedge }{C}}_{4}$
Ξ_1_	$\left(\begin{array}{c} \left\{0.2,0.5,0.4\right\}\text{,}\\ \left\{0.7,0.6,0.7\right\}\text{,}\\ \left\{0.7,0.3,0.1\right\} \end{array}\right)$	$\left(\begin{array}{c} \left\{0,0.1,0.5\right\}\text{,}\\ \left\{0.8,0.9,0.4\right\}\text{,}\\ \left\{0.3,0.2,0.9\right\} \end{array}\right)$	$\left(\begin{array}{c} \left\{0.1,0.6,0.7\right\}\text{,}\\ \left\{0.7,0.3\right\}\text{,}\\ \left\{0.5,0.8,0.2\right\} \end{array}\right)$	$\left(\begin{array}{c} \left\{0.3,0.4,0.5\right\}\text{,}\\ \left\{0.8\right\}\text{,}\\ \left\{0.4,0.7,0.3\right\} \end{array}\right)$
Ξ_2_	$\left(\begin{array}{c} \left\{0.3,0.3,0.6\right\}\text{,}\\ \left\{0.1,0.7\right\}\text{,}\\ \left\{0.9,0.1,0.3\right\} \end{array}\right)$	$\left(\begin{array}{c} \left\{0.1,0.3,0.7\right\}\text{,}\\ \left\{0.2,0.4\right\}\text{,}\\ \left\{0.4,0.3,0.7\right\} \end{array}\right)$	$\left(\begin{array}{c} \left\{0.3,0.4,0.4\right\}\text{,}\\ \left\{0.6,0.9\right\}\text{,}\\ \left\{0.9,0.2,0.4\right\} \end{array}\right)$	$\left(\begin{array}{c} \left\{0.5,0.4\right\}\text{,}\\ \left\{0.7\right\}\text{,}\\ \left\{0.4,0.9\right\} \end{array}\right)$
Ξ_3_	$\left(\begin{array}{c} \left\{0.55,0.6\right\}\text{,}\\ \left\{0.7,0.8\right\}\text{,}\\ \left\{0.4,0.3,0.8\right\} \end{array}\right)$	$\left(\begin{array}{c} \left\{0.5,0.7,0.9\right\}\text{,}\\ \left\{0.2,0.9\right\}\text{,}\\ \left\{0.9,0.2,0.5\right\} \end{array}\right)$	$\left(\begin{array}{c} \left\{0.2,0.3\right\}\text{,}\\ \left\{0.5,0.9\right\}\text{,}\\ \left\{0.4,0.5,0.1\right\} \end{array}\right)$	$\left(\begin{array}{c} \left\{0.7,0.4\right\}\text{,}\\ \left\{0.15,0.2,0.4\right\}\text{,}\\ \left\{0.2,0.3,0.6\right\} \end{array}\right)$
Ξ_4_	$\left(\begin{array}{c} \left\{0.2,0.4,0.9\right\}\text{,}\\ \left\{0.6,0.5,0.7\right\}\text{,}\\ 0.3,0.4,0.9 \end{array}\right)$	$\left(\begin{array}{c} \left\{0.2,0.8,0.5\right\}\text{,}\\ \left\{0.3,0.8\right\}\text{,}\\ \left\{0.2,0.4,0.9\right\} \end{array}\right)$	$\left(\begin{array}{c} \left\{0.2,0.4\right\}\text{,}\\ \left\{0.3,0.9\right\}\text{,}\\ \left\{0.3,0.2,0.9\right\} \end{array}\right)$	$\left(\begin{array}{c} \left\{0.4,0.7,0.9\right\}\text{,}\\ \left\{0.1,0.6\right\}\text{,}\\ \left\{0.5,0.3\right\} \end{array}\right)$

**Table 2:** Expert information.

	*P*_1_	*P*_2_
Ξ_1_	$\left(\begin{array}{c} \left(\begin{array}{c} \left(0.5,0.6,0.4\right)\text{,}\\ \left(0.7,0.8\right)\text{,}\\ \left(0.7\right) \end{array}\right)\text{,}\\ \left(\begin{array}{c} \left(0.9,0.8\right),\\ \left(0.8,0.7\right)\\ \left(0.3,0.7\right) \end{array}\right) \end{array}\right)$	$\left(\begin{array}{c} \left(\begin{array}{c} \left(0.9,0.5\right)\text{,}\\ \left(0.9,0.3\right)\text{,}\\ \left(0.7,0.8\right) \end{array}\right)\text{,}\\ \left(\begin{array}{c} \left(0.6\right),\\ \left(0.6\right),\\ \left(0.9\right) \end{array}\right) \end{array}\right)$

Ξ_2_	$\left(\begin{array}{c} \left(\begin{array}{c} \left(0.1,0.9\right)\text{,}\\ \left(0.7\right)\text{,}\\ \left(0.8,0.6\right) \end{array}\right)\text{,}\\ \left(\begin{array}{c} \left(0.5,0.8\right),\\ \left(0.5,0.3\right)\\ \left(0.8,0.9\right) \end{array}\right) \end{array}\right)$	$\left(\begin{array}{c} \left(\begin{array}{c} \left(0.9\right)\text{,}\\ \left(0.8,0.5\right)\text{,}\\ \left(0.7\right) \end{array}\right)\text{,}\\ \left(\begin{array}{c} \left(0.6,0.7\right),\\ \left(0.2\right),\\ \left(0.5,0.7\right) \end{array}\right) \end{array}\right)$

**Table 3:** Expert information.

	*P*_3_	*P*_4_
Ξ_1_	$\left(\begin{array}{c} \left(\left(\begin{array}{c} \left(0.8\right)\text{,}\\ \left(0.6\right)\text{,}\\ \left(0.7\right) \end{array}\right)\right)\text{,}\\ \left(\left(\begin{array}{c} \left(0.7\right),\\ \left(0.8,0.3\right),\\ \left(0.1\right) \end{array}\right)\right) \end{array}\right)$	$\left(\begin{array}{c} \left(\left(\begin{array}{c} \left(0.2\right)\text{,}\\ \left(0.8\right)\text{,}\\ \left(0.4,0.3\right) \end{array}\right)\right)\text{,}\\ \left(\left(\begin{array}{c} \left(0.9\right),\\ \left(0.6,0.9\right),\\ \left(0.3\right) \end{array}\right)\right) \end{array}\right)$

Ξ_2_	$\left(\begin{array}{c} \left(\left(\begin{array}{c} \left(0.8\right)\text{,}\\ \left(0.7,0.3\right)\text{,}\\ \left(0.4\right) \end{array}\right)\right)\text{,}\\ \left(\left(\begin{array}{c} \left(0.9\right),\\ \left(0.6\right),\\ \left(0.8\right) \end{array}\right)\right) \end{array}\right)$	$\left(\begin{array}{c} \left(\left(\begin{array}{c} \left(0.7\right)\text{,}\\ \left(0.4\right)\text{,}\\ \left(0.4\right) \end{array}\right)\right)\text{,}\\ \left(\left(\begin{array}{c} \left(0.5\right),\\ \left(0.7,0.4\right),\\ \left(0.4\right) \end{array}\right)\right) \end{array}\right)$

**Table 4:** Expert information.

	*P*_1_	*P*_2_
Ξ_3_	$\left(\begin{array}{c} \left(\left(\begin{array}{c} \left(0.7\right)\text{,}\\ \left(0.7,0.4\right)\text{,}\\ \left(0.9,0.3\right. \end{array}\right)\right)\text{,}\\ \left(\left(\begin{array}{c} \left(0.6,0.5\right),\\ \left(0.1\right),\\ \left(0.3,0.8\right) \end{array}\right)\right) \end{array}\right)$	$\left(\begin{array}{c} \left(\left(\begin{array}{c} \left(0.7,0.2,0.9\right)\text{,}\\ \left(0.6\right)\text{,}\\ \left(0.3,0.8\right) \end{array}\right)\right)\text{,}\\ \left(\left(\begin{array}{c} \left(0.6,0.4\right),\\ \left(0.5\right),\\ \left(0.6,0.5\right) \end{array}\right)\right) \end{array}\right)$

Ξ_4_	$\left(\begin{array}{c} \left(\left(\begin{array}{c} \left(0.3\right)\text{,}\\ \left(0.7\right)\text{,}\\ \left(0.3\right) \end{array}\right)\right)\text{,}\\ \left(\left(\begin{array}{c} \left(0.4,0.7\right),\\ \left(0.8,0.4\right),\\ \left(0.3\right) \end{array}\right)\right) \end{array}\right)$	$\left(\begin{array}{c} \left(\left(\begin{array}{c} \left(0.4\right)\text{,}\\ \left(0.4,0.6,0.2\right)\text{,}\\ \left(0.7\right) \end{array}\right)\right)\text{,}\\ \left(\left(\begin{array}{c} \left(0.8\right),\\ \left(0.4\right),\\ \left(0.7,0.8\right) \end{array}\right)\right) \end{array}\right)$

**Table 5:** Expert information.

	*P*_3_	*P*_4_
Ξ_3_	$\left(\begin{array}{c} \left(\left(\begin{array}{c} \left(0.6\right)\text{,}\\ \left(0.5\right)\text{,}\\ \left(0.2\right) \end{array}\right)\right)\text{,}\\ \left(\left(\begin{array}{c} \left(0.4\right),\\ \left(0.9,0.3\right),\\ \left(0.6\right) \end{array}\right)\right) \end{array}\right)$	$\left(\begin{array}{c} \left(\left(\begin{array}{c} \left(0.3\right)\text{,}\\ \left(0.4\right)\text{,}\\ \left(0.5\right) \end{array}\right)\right)\text{,}\\ \left(\left(\begin{array}{c} \left(0.6\right),\\ \left(0.8,0.4\right),\\ \left(0.7\right) \end{array}\right)\right) \end{array}\right)$

Ξ_4_	$\left(\begin{array}{c} \left(\left(\begin{array}{c} \left(0.3\right)\text{,}\\ \left(0.2\right)\text{,}\\ \left(0.3,0.4,0.5\right) \end{array}\right)\right)\text{,}\\ \left(\left(\begin{array}{c} \left(0.9\right),\\ \left(0.7,0.4\right),\\ \left(0.5\right) \end{array}\right)\right) \end{array}\right)$	$\left(\begin{array}{c} \left(\left(\begin{array}{c} \left(0.4,0.6,0.2\right)\text{,}\\ \left(0.7\right)\text{,}\\ \left(0.4\right) \end{array}\right)\right)\text{,}\\ \left(\left(\begin{array}{c} \left(0.6,0.5\right),\\ \left(0.7\right),\\ \left(0.3,0.8\right) \end{array}\right)\right) \end{array}\right)$

**Table 6:** Aggregated details using SV-NHFRWA.

Ξ_1_	$\left(\begin{array}{c} \left(\begin{array}{c} \left\{\begin{array}{c} 0.6603,0.4494,0.6751,0.4734,\\ 0.6477,0.4290 \end{array}\right\}\text{,}\\ \left\{0.7840,0.5639,0.8053,0.5792\right\}\text{,}\\ \left\{0.5594,0.4988,0.5825,0.5192\right\} \end{array}\right)\text{,}\\ \left(\begin{array}{c} \left\{0.8308,0.8057\right\},\\ \left\{0.6541,0.6974,0.8057,0.5930,0.6368,\right.\\ \left(0.7490,0.6790,0.5773\right\},\\ \left\{0.3737,0.4427\right\} \end{array}\right) \end{array}\right)$
Ξ_2_	$\left(\begin{array}{c} \left(\begin{array}{c} \left\{0.7419,0.8337\right\}\text{,}\\ \left\{0.5825,0.5352,0.5059,0.4648\right\}\text{,}\\ \left\{0.5435,0.5131\right\} \end{array}\right)\text{,}\\ \left(\begin{array}{c} \left\{0.6019,0.6348,0.6686,0.6960\right\},\\ \left\{0.4425,0.3538,0.3996,0.3194\right\},\\ \left\{0.5266,0.5825,0.5391,0.5964\right\} \end{array}\right) \end{array}\right)$

**Table 7:** Aggregated information using SV-NHFRWA.

Ξ_3_	$\left(\begin{array}{c} \left(\begin{array}{c} \left\{0.5667,0.4184,0.6883\right\}\text{,}\\ \left\{0.5166,0.4619\right\}\text{,}\\ \left\{0.4402,0.5908,0.3534,0.4743\right\}\text{,} \end{array}\right)\text{,}\\ \left(\begin{array}{c} \left\{0.5834,0.5296,0.5644,0.50821\right\},\\ \left\{0.4638,0.3515,0.4156,0.3149\right\},\\ \left\{0.5556,0.5260,0.6760,0.6400\right\} \end{array}\right) \end{array}\right)$
Ξ_4_	$\left(\begin{array}{c} \left(\begin{array}{c} \left\{0.3716,0.4657,0.2950\right\}\text{,}\\ \left\{0.5221,0.5897,0.4241\right\}\text{,}\\ \left\{0.4340,0.4467,0.4567\right\} \end{array}\right)\text{,}\\ \left(\begin{array}{c} \left\{0.6933,0.6646,0.7330,0.7080\right\},\\ \left\{0.6078,0.5748,0.5292,0.5004\right\}\\ \left\{0.4071,0.6027,0.4237,0.6273\right\} \end{array}\right) \end{array}\right)$

**Table 8:** Aggregated information using SV-NHFRWG.

Ξ_1_	$\left(\begin{array}{c} \left(\begin{array}{c} \left\{\begin{array}{c} 0.4333,0.3633,0.4494,0.3767,\\ 0.4144,0.3474,0.3767 \end{array}\right\}\text{,}\\ \left\{1.0000,0.9999,1.0000,0.9999\right\}\text{,}\\ \left\{0.9999,0.9999,1.0000,0.9999\right\} \end{array}\right)\text{,}\\ \left(\begin{array}{c} \left\{0.7771,0.7590\right\},\\ \left\{\begin{array}{c} 1.0000,1.0000,1.0000,0.9999,1.0000,\\ 1.0000,1.0000,0.9998 \end{array}\right\},\\ \left\{0.9995,0.9998\right\} \end{array}\right) \end{array}\right)$
Ξ_2_	$\left(\begin{array}{c} \left(\begin{array}{c} \left\{0.5183,0.8044\right\}\text{,}\\ \left\{0.6495,0.6185,0.5386,0.4978\right\}\text{,}\\ \left\{0.6088,0.5506\right\} \end{array}\right)\text{,}\\ \left(\begin{array}{c} \left\{0.5601,0.5866,0.6153,0.6444\right\},\\ \left\{0.5410,0.3944,0.5091,0.3522\right\},\\ \left\{0.5914,0.6495,0.6443,0.6949\right\} \end{array}\right) \end{array}\right)$

**Table 9:** Aggregated information using SV-NHFRWG.

Ξ_3_	$\left(\begin{array}{c} \left(\begin{array}{c} \left\{0.4911,0.3373,0.5296\right\}\text{,}\\ \left\{0.5458,0.4783\right\}\text{,}\\ \left\{0.5798,0.7115,0.3799,0.5742\right\} \end{array}\right)\text{,}\\ \left(\begin{array}{c} \left\{0.5762,0.5102,0.5555,0.4919\right\},\\ \left\{0.6681,0.4850,0.5969,0.3744\right\},\\ \left\{0.6013,0.5737,0.6896,0.6681\right\} \end{array}\right) \end{array}\right)$
Ξ_4_	$\left(\begin{array}{c} \left(\begin{array}{c} \left\{0.3669,0.4315,0.2781\right\}\text{,}\\ \left\{0.5926,0.6393,0.5559\right\}\text{,}\\ \left\{0.4896,0.4974,0.5065\right\} \end{array}\right)\text{,}\\ \left(\begin{array}{c} \left\{0.6281,0.5839,0.7025,0.6531\right\},\\ \left\{0.6594,0.6350,0.5757,0.5453\right\},\\ \left\{0.4751,0.6820,0.5352,0.7184\right\} \end{array}\right) \end{array}\right)$

**Table 10:** Score values.

Operators	Δ(Ξ_1_)	Δ(Ξ_2_)	Δ(Ξ_3_)	Δ(Ξ_4_)
*SV*-NHFRWA	0.7055	0.7213	0.6893	0.6979
*SV*-NHFRWG	0.6942	0.7002	0.6795	0.6856

**Table 11:** Ranking of the alternatives.

Operators	Score	Best alternative
*SV*-NHFRWA	Δ(Ξ_2_) > Δ(Ξ_1_) > Δ(Ξ_4_) > Δ(Ξ_3_)	Ξ_2_
*SV*-NHFRWG	Δ(Ξ_2_) > Δ(Ξ_1_) > Δ(Ξ_4_) > Δ(Ξ_3_)	Ξ_2_

**Table 12:** Expert information updated.

	*P*_1_	*P*_2_
Ξ_1_	$\left(\begin{array}{c} \left(\begin{array}{c} \left(0.5,0.6,0.4\right)\text{,}\\ \left(0.7,0.8\right)\text{,}\\ \left(0.7\right) \end{array}\right)\text{,}\\ \left(\begin{array}{c} \left(0.9,0.8\right),\\ \left(0.8,0.7\right)\\ \left(0.3,0.7\right) \end{array}\right) \end{array}\right)$	$\left(\begin{array}{c} \left(\begin{array}{c} \left(0.7,0.8\right)\text{,}\\ \left(0.9,0.3\right)\text{,}\\ \left(0.9,0.5\right) \end{array}\right)\text{,}\\ \left(\begin{array}{c} \left(0.9\right),\\ \left(0.6\right),\\ \left(0.6\right) \end{array}\right) \end{array}\right)$

Ξ_2_	$\left(\begin{array}{c} \left(\begin{array}{c} \left(0.1,0.9\right)\text{,}\\ \left(0.7\right)\text{,}\\ \left(0.8,0.6\right) \end{array}\right)\text{,}\\ \left(\begin{array}{c} \left(0.5,0.8\right),\\ \left(0.5,0.3\right)\\ \left(0.8,0.9\right) \end{array}\right) \end{array}\right)$	$\left(\begin{array}{c} \left(\begin{array}{c} \left(0.7\right)\text{,}\\ \left(0.8,0.5\right)\text{,}\\ \left(0.9\right) \end{array}\right)\text{,}\\ \left(\begin{array}{c} \left(0.5,0.7\right),\\ \left(0.2\right),\\ \left(0.6,0.7\right) \end{array}\right) \end{array}\right)$

**Table 13:** Expert information updated.

	*P*_3_	*P*_4_
Ξ_1_	$\left(\begin{array}{c} \left(\left(\begin{array}{c} \left(0.8\right)\text{,}\\ \left(0.6\right)\text{,}\\ \left(0.7\right) \end{array}\right)\right)\text{,}\\ \left(\left(\begin{array}{c} \left(0.7\right),\\ \left(0.8,0.3\right),\\ \left(0.1\right) \end{array}\right)\right) \end{array}\right)$	$\left(\begin{array}{c} \left(\left(\begin{array}{c} \left(0.4,0.3\right)\text{,}\\ \left(0.8\right)\text{,}\\ \left(0.2\right) \end{array}\right)\right)\text{,}\\ \left(\left(\begin{array}{c} \left(0.3\right),\\ \left(0.6,0.9\right),\\ \left(0.9\right) \end{array}\right)\right) \end{array}\right)$

Ξ_2_	$\left(\begin{array}{c} \left(\left(\begin{array}{c} \left(0.8\right)\text{,}\\ \left(0.7,0.3\right)\text{,}\\ \left(0.4\right) \end{array}\right)\right)\text{,}\\ \left(\left(\begin{array}{c} \left(0.9\right),\\ \left(0.6\right),\\ \left(0.8\right) \end{array}\right)\right) \end{array}\right)$	$\left(\begin{array}{c} \left(\left(\begin{array}{c} \left(0.4\right)\text{,}\\ \left(0.4\right)\text{,}\\ \left(0.7\right) \end{array}\right)\right)\text{,}\\ \left(\left(\begin{array}{c} \left(0.4\right),\\ \left(0.7,0.4\right),\\ \left(0.5\right) \end{array}\right)\right) \end{array}\right)$

**Table 14:** Expert information updated.

	*P*_1_	*P*_2_
Ξ_3_	$\left(\begin{array}{c} \left(\left(\begin{array}{c} \left(0.9,0.3\right)\text{,}\\ \left(0.7,0.4\right)\text{,}\\ \left(0.7\right) \end{array}\right)\right)\text{,}\\ \left(\left(\begin{array}{c} \left(0.3,0.8\right),\\ \left(0.1\right),\\ \left(0.6,0.5\right) \end{array}\right)\right) \end{array}\right)$	$\left(\begin{array}{c} \left(\left(\begin{array}{c} \left(0.7,0.2,0.9\right)\text{,}\\ \left(0.6\right)\text{,}\\ \left(0.3,0.8\right) \end{array}\right)\right)\text{,}\\ \left(\left(\begin{array}{c} \left(0.6,0.4\right),\\ \left(0.5\right),\\ \left(0.6,0.5\right) \end{array}\right)\right) \end{array}\right)$

Ξ_4_	$\left(\begin{array}{c} \left(\left(\begin{array}{c} \left(0.3\right)\text{,}\\ \left(0.7\right)\text{,}\\ \left(0.3\right) \end{array}\right)\right)\text{,}\\ \left(\left(\begin{array}{c} \left(0.3\right),\\ \left(0.8,0.4\right),\\ \left(0.4,0.7\right) \end{array}\right)\right) \end{array}\right)$	$\left(\begin{array}{c} \left(\left(\begin{array}{c} \left(0.4\right)\text{,}\\ \left(0.4,0.6,0.2\right)\text{,}\\ \left(0.7\right) \end{array}\right)\right)\text{,}\\ \left(\left(\begin{array}{c} \left(0.8\right),\\ \left(0.4\right),\\ \left(0.7,0.8\right) \end{array}\right)\right) \end{array}\right)$

**Table 15:** Expert information updated.

	*P*_3_	*P*_4_
Ξ_3_	$\left(\begin{array}{c} \left(\left(\begin{array}{c} \left(0.2\right)\text{,}\\ \left(0.5\right)\text{,}\\ \left(0.6\right) \end{array}\right)\right)\text{,}\\ \left(\left(\begin{array}{c} \left(0.6\right),\\ \left(0.9,0.3\right),\\ \left(0.4\right) \end{array}\right)\right) \end{array}\right)$	$\left(\begin{array}{c} \left(\left(\begin{array}{c} \left(0.3\right)\text{,}\\ \left(0.4\right)\text{,}\\ \left(0.5\right) \end{array}\right)\right)\text{,}\\ \left(\left(\begin{array}{c} \left(0.6\right),\\ \left(0.8,0.4\right),\\ \left(0.7\right) \end{array}\right)\right) \end{array}\right)$

Ξ_4_	$\left(\begin{array}{c} \left(\left(\begin{array}{c} \left(0.3,0.4,0.5\right)\text{,}\\ \left(0.2\right)\text{,}\\ \left(0.3\right) \end{array}\right)\right)\text{,}\\ \left(\left(\begin{array}{c} \left(0.5\right),\\ \left(0.7,0.4\right),\\ \left(0.9\right) \end{array}\right)\right) \end{array}\right)$	$\left(\begin{array}{c} \left(\left(\begin{array}{c} \left(0.4,0.6,0.2\right)\text{,}\\ \left(0.7\right)\text{,}\\ \left(0.4\right) \end{array}\right)\right)\text{,}\\ \left(\left(\begin{array}{c} \left(0.6,0.5\right),\\ \left(0.7\right),\\ \left(0.3,0.8\right) \end{array}\right)\right) \end{array}\right)$

**Table 16:** Updated aggregated details using SV-NHFRWA.

Ξ_1_	$\left(\begin{array}{c} \left(\begin{array}{c} \left\{\begin{array}{c} 0.5790,0.5522,0.6272,0.6035,0.5974,0.5718,0.6435,\\ 0.6208,0.5634,0.5356,0.6134,0.5888 \end{array}\right\}\text{,}\\ \left\{0.7840,0.5639,0.8053,0.5792\right\}\text{,}\\ \left\{0.4573,0.3834\right\} \end{array}\right)\text{,}\\ \left(\begin{array}{c} \left\{0.7569,0.7208\right\},\\ \left\{0.6541,0.7693,0.5930,0.6974,0.6368,\right.\\ \left(0.7490,0.5773,0.6790\right\},\\ \left\{0.5135,0.6084\right\} \end{array}\right) \end{array}\right)$
Ξ_2_	$\left(\begin{array}{c} \left(\begin{array}{c} \left\{0.5265,0.6949\right\}\text{,}\\ \left\{0.5825,0.5352,0.5059,0.4648\right\}\text{,}\\ \left\{0.7331,0.6921\right\} \end{array}\right)\text{,}\\ \left(\begin{array}{c} \left\{0.5421,0.6072,0.6188,0.6730\right\},\\ \left\{0.4425,0.3538,0.3996,0.3194\right\},\\ \left\{0.6081,0.6013,0.6226,0.6520\right\} \end{array}\right) \end{array}\right)$

**Table 17:** Updated aggregated information using SV-NHFRWA.

Ξ_3_	$\left(\begin{array}{c} \left(\begin{array}{c} \left\{0.6272,0.4996,0.7319,0.4498,0.2616,0.6043\right\}\text{,}\\ \left\{0.5166,0.4619\right\}\text{,}\\ \left\{0.4673,0.6271\right\}\text{,} \end{array}\right)\text{,}\\ \left(\begin{array}{c} \left\{0.4002,0.3226,0.5332,0.4728\right\},\\ \left\{0.3314\right\},\\ \left\{0.5578,0.5218,0.5378,0.5092\right\} \end{array}\right) \end{array}\right)$
Ξ_4_	$\left(\begin{array}{c} \left(\begin{array}{c} \left\{\begin{array}{c} 0.3716,0.4657,0.2950,0.3812,0.4739,\\ 0.3057,0.3924,0.4834 \end{array}\right\}\text{,}\\ \left\{0.5221,0.5897,0.4241\right\}\text{,}\\ \left\{0.4340\right\} \end{array}\right)\text{,}\\ \left(\begin{array}{c} \left\{0.6284,0.5937\right\},\\ \left\{0.6078,0.5748,0.5292,0.5004\right\}\\ \left\{\begin{array}{c} 0.4573,0.6770,0.4760,0.7047,0.5115,\\ 0.7572,0.5324,0.7881 \end{array}\right\} \end{array}\right) \end{array}\right)$

**Table 18:** Updated aggregated details using SV-NHFRWG.

Ξ_1_	$\left(\begin{array}{c} \left(\begin{array}{c} \left\{\begin{array}{c} 0.5302,0.4726,0.5519,0.4919,0.5499,0.4901,0.5724,\\ 0.5102,0.5071,0.4520,0.5278,0.4704 \end{array}\right\}\text{,}\\ \left\{1.0000,0.9999,1.0000,0.9999\right\}\text{,}\\ \left\{1.0000,0.9998\right\} \end{array}\right)\text{,}\\ \left(\begin{array}{c} \left\{0.5656,0.5524\right\},\\ \left\{1.0000,1.0000,0.9999,1.0000,1.0000,\right.\\ \left(1.0000,0.9998,1.0000\right\},\\ \left\{0.9997,0.9999\right\} \end{array}\right) \end{array}\right)$
Ξ_2_	$\left(\begin{array}{c} \left(\begin{array}{c} \left\{0.3843,0.5964\right\}\text{,}\\ \left\{0.6495,0.6185,0.5386,0.4978\right\}\text{,}\\ \left\{0.7868,0.7551\right\} \end{array}\right)\text{,}\\ \left(\begin{array}{c} \left\{0.4850,0.5365,0.5328,0.5894\right\},\\ \left\{0.5410,0.3944,0.5091,0.3522\right\},\\ \left\{0.6448,0.6257,0.6908,0.7163\right\} \end{array}\right) \end{array}\right)$

**Table 19:** Updated aggregated information using SV-NHFRWG.

Ξ_3_	$\left(\begin{array}{c} \left(\begin{array}{c} \left\{0.4627,0.3178,0.4990,0.3715,0.2551,0.4005\right\}\text{,}\\ \left\{0.5458,0.4783\right\}\text{,}\\ \left\{0.4214,0.6027\right\}\text{,} \end{array}\right)\text{,}\\ \left(\begin{array}{c} \left\{0.3547,0.3140,0.4315,0.3821\right\},\\ \left\{0.3951\right\},\\ \left\{0.5627,0.5324,0.5427,0.5110\right\} \end{array}\right) \end{array}\right)$
Ξ_4_	$\left(\begin{array}{c} \left(\begin{array}{c} \left\{\begin{array}{c} 0.3669,0.4315,0.2781,0.3776,0.4441,\\ 0.2862,0.3862,0.4542 \end{array}\right\}\text{,}\\ \left\{0.5926,0.6393,0.5559\right\}\text{,}\\ \left\{0.4896\right\} \end{array}\right)\text{,}\\ \left(\begin{array}{c} \left\{0.5591,0.5198\right\},\\ \left\{0.6594,0.6350,0.5757,0.5453\right\}\\ \left\{\begin{array}{c} 0.5667,0.7375,0.6163,0.7675,0.6228,\\ 0.7715,0.6660,0.7976 \end{array}\right\} \end{array}\right) \end{array}\right)$

**Table 20:** Score values.

Operators	Δ(Ξ_1_)	Δ(Ξ_2_)	Δ(Ξ_3_)	Δ(Ξ_4_)
*SV*-NHFRWA (updated)	0.6429	0.6644	0.5950	0.6621
*SV*-NHFRWG (updated)	0.6330	0.6575	0.6105	0.5959

**Table 21:** Ranking of the alternatives.

Operators	Score	Best alternative
*SV*-NHFRWA (updated)	Δ(Ξ_2_) > Δ(Ξ_4_) > Δ(Ξ_1_) > Δ(Ξ_3_)	Ξ_1_
*SV*-NHFRWG (updated)	Δ(Ξ_2_) > Δ(Ξ_1_) > Δ(Ξ_4_) > Δ(Ξ_3_)	Ξ_1_

## Data Availability

The data supporting this study's findings are available from Muhammad Kamran upon reasonable request.
